# Supplementary material for: Overexpression of Igf2-derived Mir483 inhibits Igf1 expression and leads to developmental growth restriction and metabolic dysfunction in mice
Source: Cell Rep. Author manuscript; Available in PMC 2025 Jan 12. (PMC7617298; doi:10.1016/j.celrep.2024.114750)
Supplement: Supplementary Material [file EMS202134-supplement-Supplementary_Material.zip › 1-s2.0-S221112472401101X-mmc1.pdf]

Cell Reports, Volume *43*

Supplemental information

Overexpression of *Igf2*-derived *Mir483* inhibits *Igf1* expression and leads to developmental growth restriction and metabolic dysfunction in mice

Ionel Sandovici, Denise S. Fernandez-Twinn, Niamh Campbell, Wendy N. Cooper, Yoichi Sekita, Ilona Zvetkova, David Ferland-McCollough, Haydn M. Prosser, Lila M. Oyama, Lucas C. Pantaleão, Danilo Cimadomo, Karina Barbosa de Queiroz, Cecilia S.K. Cheuk, Nicola M. Smith, Richard G. Kay, Robin Antrobus, Katharina Hoelle, Marcella K.L. Ma, Noel H. Smith, Stefan H. Geyer, Lukas F. Reissig, Wolfgang J. Weninger, Kenneth Siddle, Anne E. Willis, Brian Y.H. Lam, Martin Bushell, Susan E. Ozanne, and Miguel Constância

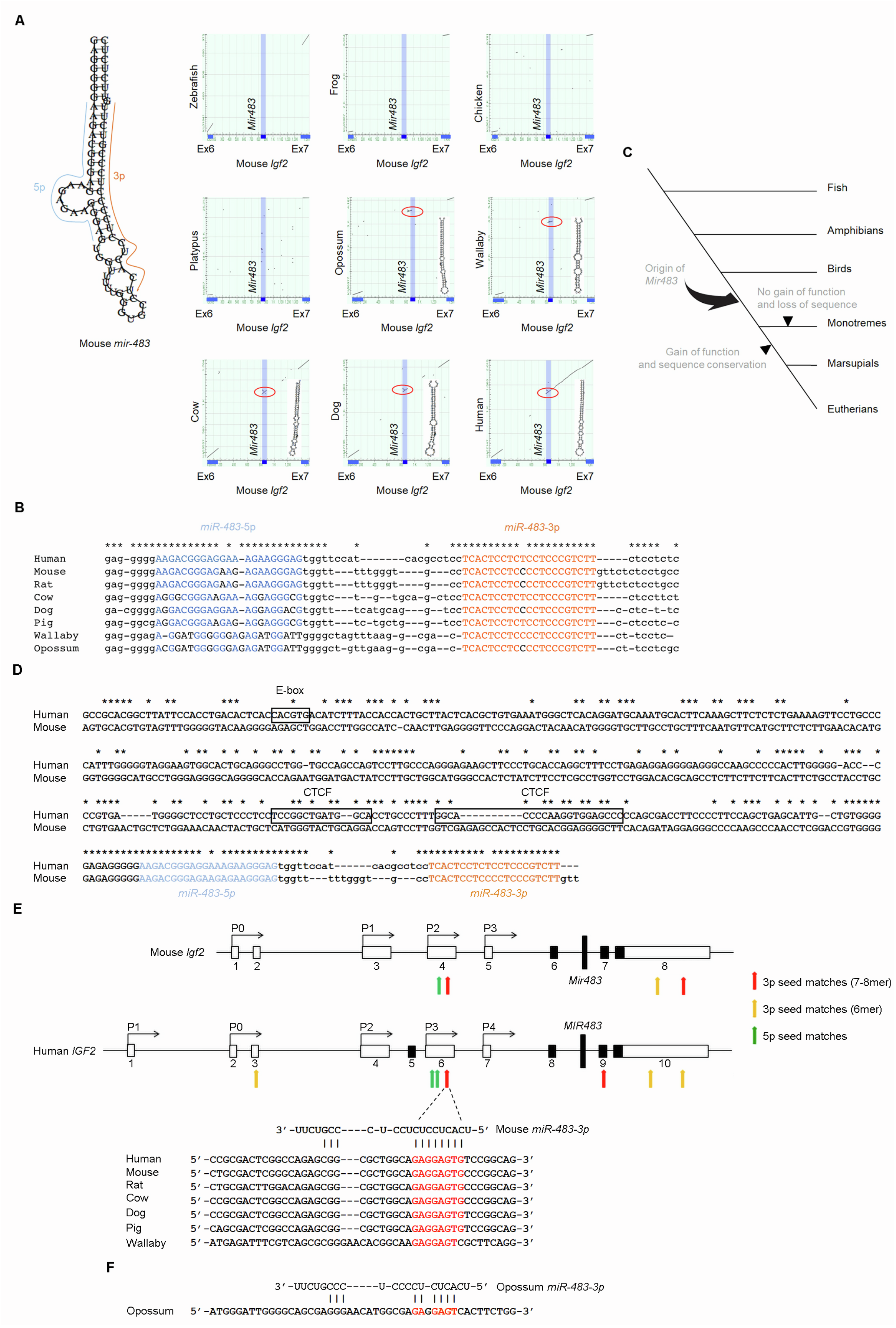
A
Mouse mir-483
5p
3p
Zebrafish
Frog
Chicken
Platypus
Opossum
Wallaby
Cow
Dog
Human
Mir483
Ex6
Ex7
Mouse Igf2
C
Fish
Amphibians
Birds
Origin of Mir483
No gain of function and loss of sequence
Monotremes
Gain of function and sequence conservation
Marsupials
Eutherians
B
miR-483-5p
miR-483-3p
Human
Mouse
Rat
Cow
Dog
Pig
Wallaby
Opossum
D
E-box
CTCF
CTCF
Human
Mouse
miR-483-5p
miR-483-3p
E
Mouse Igf2
P0
P1
P2
P3
Mir483
3p seed matches (7-8mer)
3p seed matches (6mer)
5p seed matches
Human IGF2
P1
P0
P2
P3
P4
MIR483
3′-UUCUGCC----C-U-CCUCUCCUCACU-5′ Mouse miR-483-3p
Human 5′-CCGCGACTCGGCCAGAGCGG---CGCTGGCAGAGGAGTGTCCGGCAG-3′
Mouse 5′-CTGCGACTCGGGCAGAGCGG---CGCTGGCAGAGGAGTGCCCGGCAG-3′
Rat 5′-CTGCGACTTGGACAGAGCGG---CGCTGGCAGAGGAGTGCCCGGCAG-3′
Cow 5′-CCGCGACTCGGCCAGAGCGG---CGCTGGCAGAGGAGTGCCCGGCAG-3′
Dog 5′-CCGCGACTCGGCCAGAGCGG---CGCTGGCAGAGGAGTGTCCGGCAG-3′
Pig 5′-CAGCGACTCGGCCAGAGCGG---CGCTGGCAGAGGAGTGTCCGGCAG-3′
Wallaby 5′-ATGAGATTTCGTCAGCGCGGGAACACGGCAAGAGGAGTCGCTTCAGG-3′
F
3′-UUCUGCCC-----U-CCCCU-CUCACU-5′ Opossum miR-483-3p
Opossum 5′-ATGGGATTGGGGCAGCGAGGGAACATGGCGAGAGGAGTCACTTCTGG-3′

Figure S1. Conservation between species of *Mir483* sequence and putative *Mir483* regulatory elements and target sequences at the *Igf2* locus. Related to Figure 1.

(A) *Mir483* is conserved among eutherian mammals and marsupials. The Blast2 program (http://www.ncbi.nlm.nih.gov/) was used to investigate the conservation of *Mir483* sequence among vertebrates. Genomic sequences including *Igf2*'s exons 6 and 7, and intron 6 of zebrafish, frog, chicken, platypus, opossum, wallaby, cow, dog, human, and mouse were extracted from public data bases. The mouse genome was aligned with other genomes. *Igf2* exons 6 and 7 of the mouse genome and *Mir483* are highlighted by dark blue on the X axes. Grey dots indicate sequence conservation and those corresponding to *Mir483* sequence are emphasized by red circles. The predicted secondary structures of *mir-483* in eutherian mammals and marsupials were drawn using ViennaRNA (http://www.tbi.univie.ac.at/~ivo/RNA/), with that of mouse being shown in the top left corner of this panel. (B) Sequences of eutherian mammals and marsupials corresponding to *Mir483* were aligned. *miR-483-5p* and *miR-483-3p* are highlighted in blue and orange, respectively, and nucleotides conserved between human and mouse are indicated with stars. In some of the species the annotations for the two arms of *miR-483* are based on *in silico* predictions rather than on experimental evidence. (C) Schematic illustration of the evolution of *Mir483* in vertebrates. *Mir483* emerged in the *Igf2* intron 6 region before the divergence of the therians and monotremes. In the therian lineage, *Mir483* acquired function and became conserved in the course of evolution, whereas in the monotreme lineage, *Mir483* did not gain any function or lost it, as its sequence was not conserved. (D) Sequence alignment of the human *MIR483* promoter with the equivalent sequence in the mouse shows poor conservation. The E-box and CTCF binding sites were previously identified as regulatory elements within the human *MIR483* promoter. (E) Top: schematic representation of conserved *Igf2* 5'UTR and 3'UTR sequences between mouse and human containing *miR-483*-3p and *miR-483*-5p seed sites. Genomic features are not drawn to scale and are for representation purposes only. Bottom: the sequence for a *miR-483*-3p seed site mapping to the untranslated mouse exon 4, driven by the *Igf2*-P2 promoter (equivalent to the untranslated human exon 6 driven by *IGF2*-P3 promoter) is conserved in the eutherian mammals and the marsupial wallaby. (F) In opossum, the putative *miR-483*-3p seed site within *Igf2* is interrupted by a mistmatch and for that reason it is unlikely to be functional.

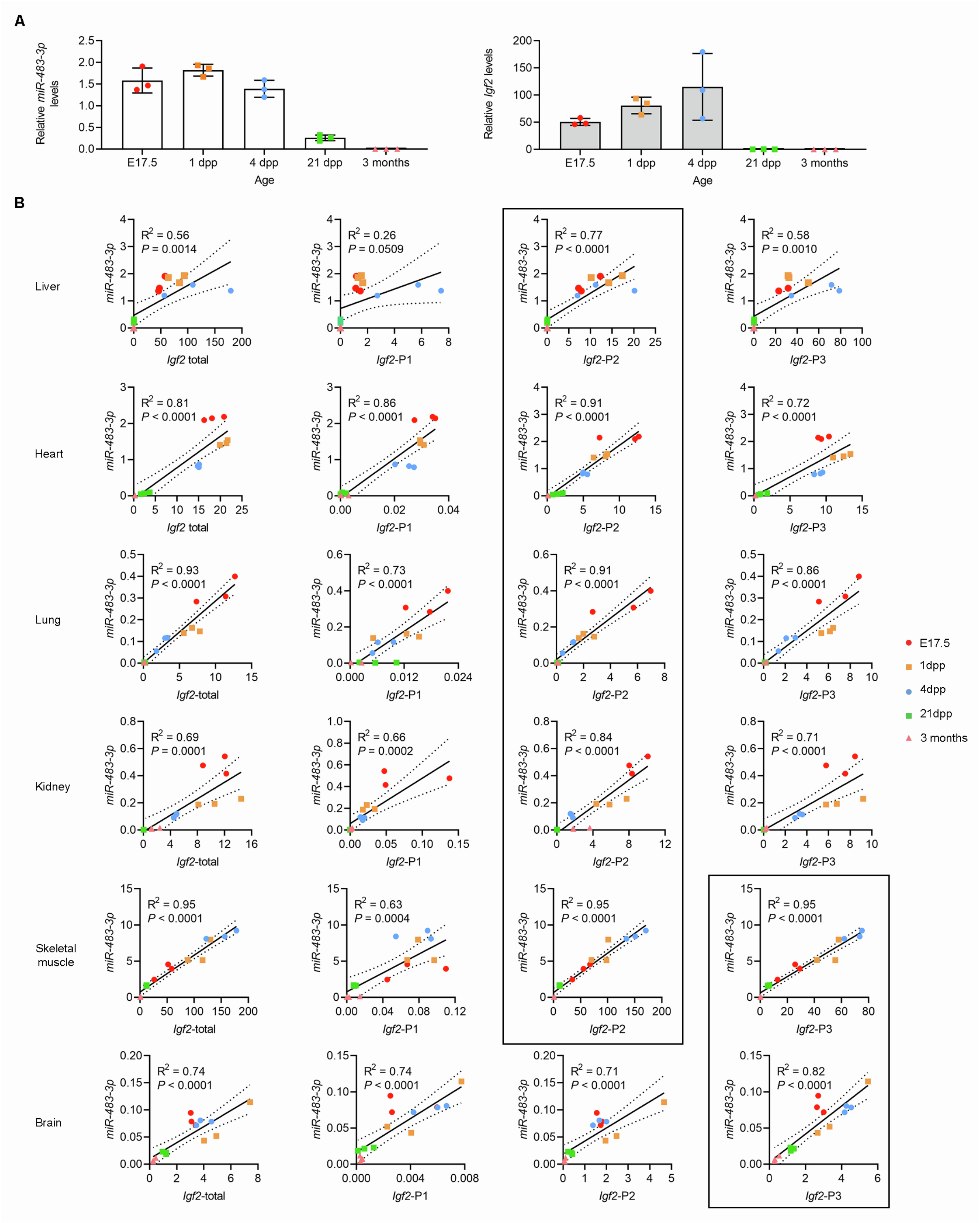

Figure S2. Developmental expression of *miR-483-3p* compared to *Igf2* in prenatal and postnatal organs. Related to Figure 1.

(A) Levels of both *miR-483-3p* and *Igf2* in the liver reach a peak in perinatal life and decrease rapidly around weaning, with very low expression levels found in adult life. (B) Linear correlation coefficients (R^2) between *miR-483-3p* and *Igf2* transcripts are strongest for the *Igf2*-P2 isoform in

liver, heart, lung and kidney, for *Igf2*-P2 and *Igf2*-P3 in the skeletal muscle and for *Igf2*-P3 in the brain (all highlighted by black contour). For all graphs, expression of *miR-483-3p* was normalized against the geometrical mean of *Snord70*/*snoRNA234*, *Snord68*/*snoRNA202*, and *miR-26b*, and expression of *Igf2* transcript isoforms was normalized against the geometrical means of *Ppia*, *Pmm1*, *Hprt*, *Sdha*, *Tbp* and *Gapdh*. Data are presented as individual values, with averages ± SD in (A) and individual values in (B) (n=3 samples for each developmental time point).

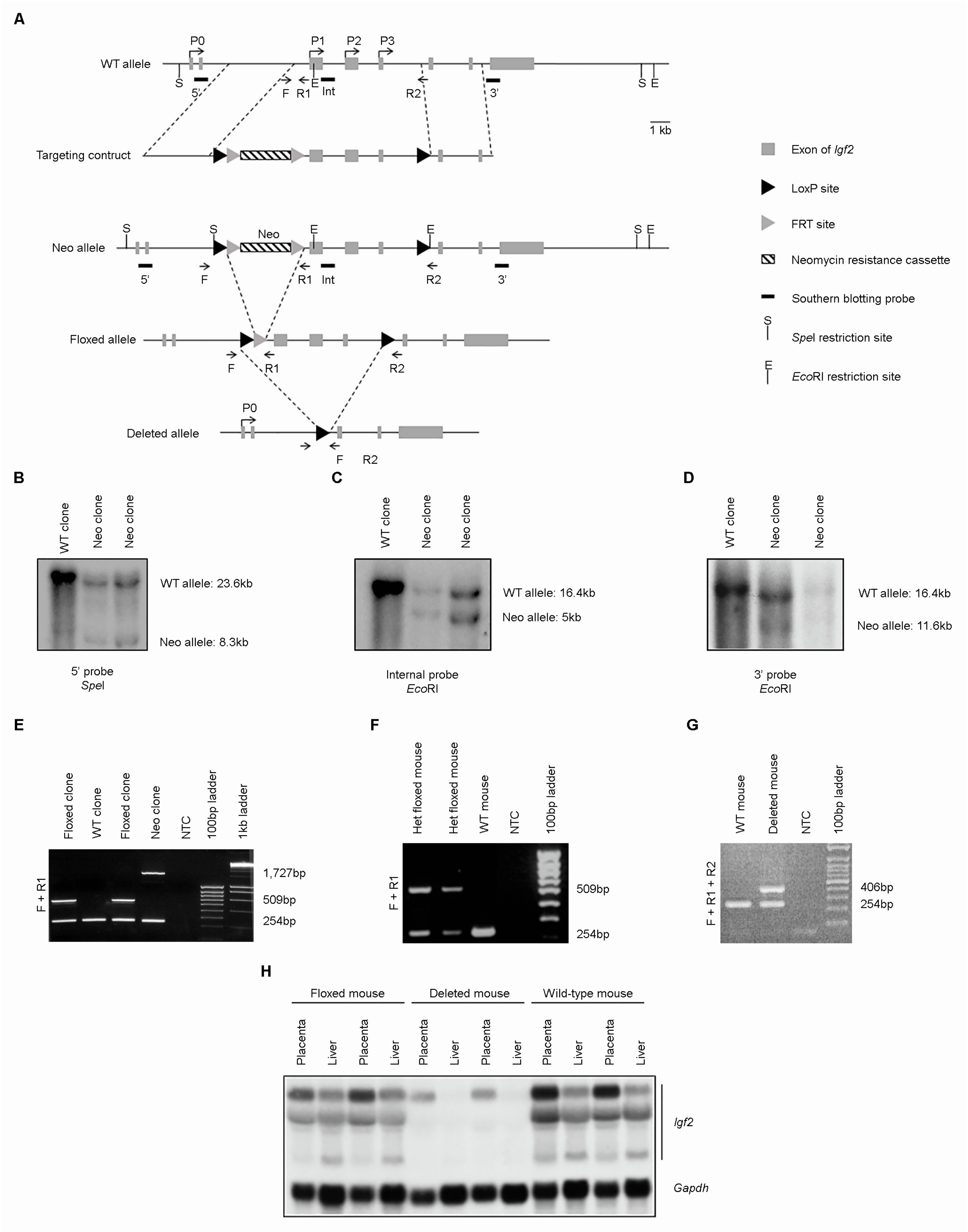

Figure S3. Generation of the *Igf2*$^{\Delta(P1\text{-}P3)}$ model. Related to Figure 1.

(A) Diagram showing the wild-type (WT) locus and the targeting construct, followed by the allele configurations after homologous recombination in ES cells, *i.e.* Neo allele, the floxed allele (after removal of the neo gene by Flpe recombinase) and the deleted allele after *Cre*-mediated recombination between LoxP sites. Probes used for Southern blot analysis (5', Int – internal, and 3')

are shown as short horizontal black lines, selected restriction sites are indicated by E (*Eco*RI) or S (*Spe*I) and genotyping primers (F, R1 and R2) are shown by arrows. Genomic features are not drawn to scale and are for representation purposes only. (B) Southern blotting of DNA extracted from ES cell clones and digested with *Spe*I was used to confirm correct 5' targeting. (C) Southern blotting of DNA extracted from ES cell clones and digested with *Eco*RI was used to confirm correct Int targeting. (D) Southern blotting of DNA extracted from ES cell clones and digested with *Eco*RI was used to confirm correct 3' targeting. (E) PCR confirmation of efficient removal of the neomycin cassette by FLPe-FRT recombination using primers F+R1 (NTC – no template control). (F) PCR genotyping of heterozygous floxed mice using primers F+R1. (G) PCR confirmation of efficient deletion of the floxed P1-P3 promoters, using primers F+R1+R2. (H) Northern blot analysis of *Igf2* transcripts and *Gapdh* internal control in placenta and liver at E18.5 (top band observed in placentae of deleted mice corresponds to *Igf2*-P0 transcript that remains expressed upon deletion of the P1-P3 fetal *Igf2* promoters).

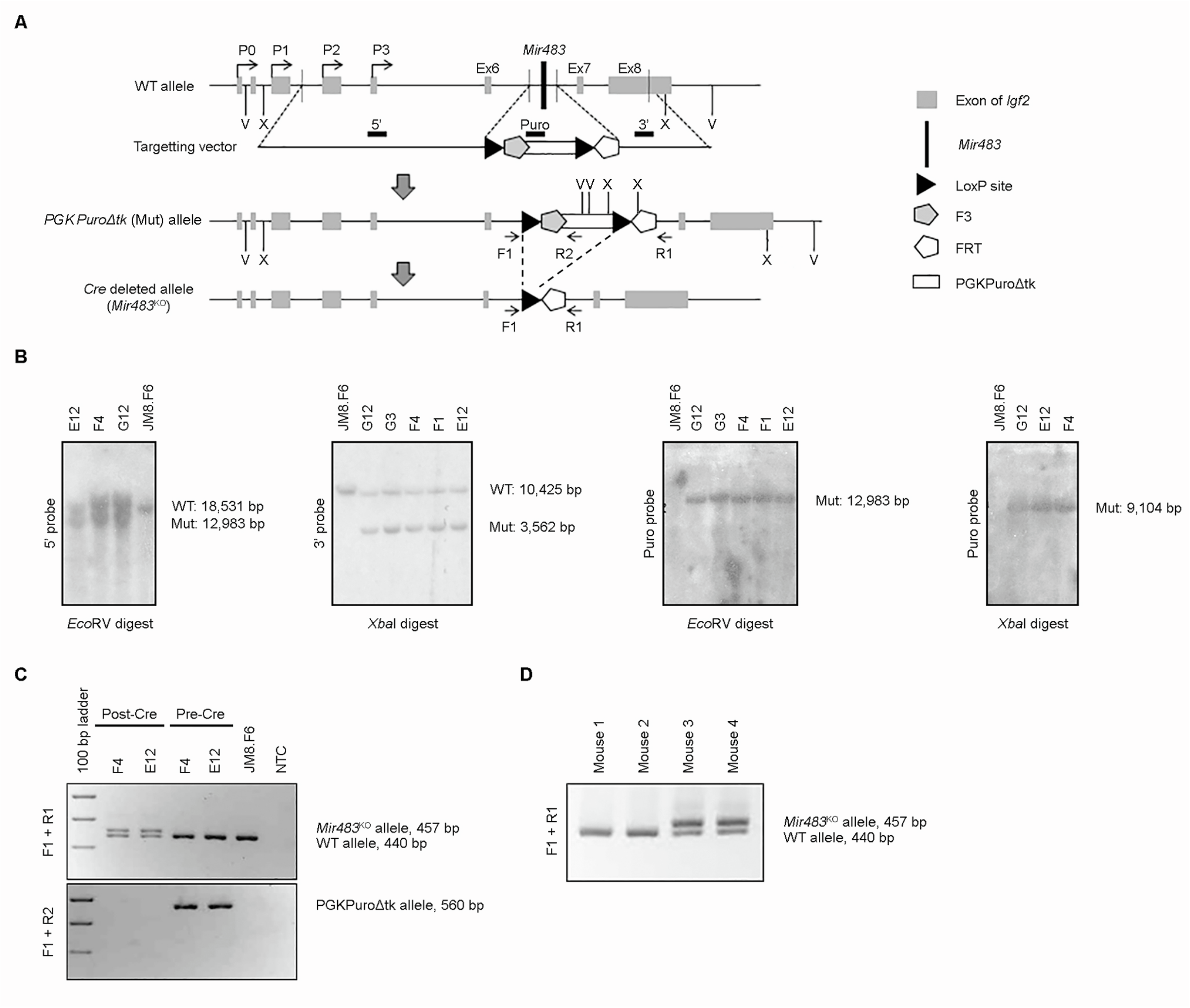

Figure S4. Generation of the *Mir483* specific knockout. Related to Figure 2.

(A) Diagram showing the wild-type (WT) locus and the targeting vector, the *PuroΔtk* allele after homologous recombination and the allele after *in vitro Cre* deletion (*Mir483*^{KO}). Probes used for Southern blot analysis (5', Puro and 3') are shown as short black lines above the targeting vector, selected restriction sites are indicated by V (*Eco*RV) or X (*Xba*I) and genotyping primers (F1, R1 and R2) are shown by arrows. Genomic features are not drawn to scale and are for representation purposes only. (B) Southern blotting of ES cell clones was used to confirm correct targeting. (C) *In vitro Cre* deletion in ES cell clones was confirmed by PCR (using the primers indicated in the figure) before (Pre-*Cre*) and after (Post-*Cre*) transfection with a plasmid encoding a *Cre* recombinase. Parental JM8.F6 ES cell DNA was also amplified, NTC – no template negative control. (D) PCR genotyping in tail DNA using primers F1 and R1.

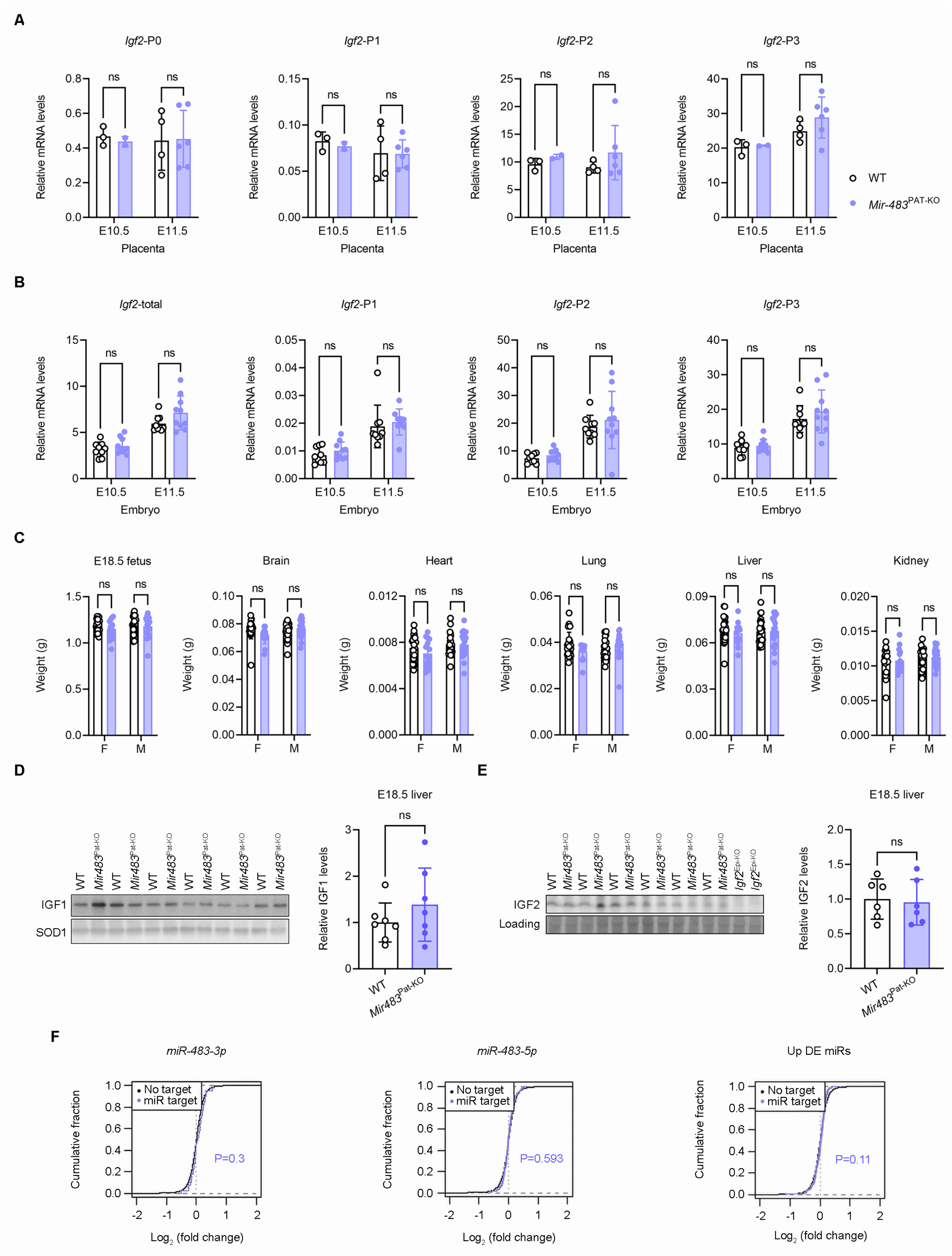

Figure S5. Expression of *Igf2* isoforms and organ weights and analyses of cumulative fractions mRNA fold changes for predicted targets of DEMs in the *Mir483*$^{Pat-KO}$ knockout. Related to Figure 2. (A) Relative mRNA expression of *Igf2* isoforms at E10.5 and E11.5 measured by RT-qPCR levels in placentae (n=2-6 samples per group). Levels of *Igf2* transcripts were normalized against the

geometrical mean of *Gapdh*, *Pmm1* and *Ppia*. (B) Relative expression of total *Igf2* and its isoforms at E10.5 and E11.5 measured by RT-qPCR levels in whole embryos (n=8-10 samples per group). Levels of *Igf2* transcripts were normalized against the geometrical mean of *Gapdh*, *Pmm1* and *Ppia*. (C) Fetus and organ weights at E18.5 (n=15-26 per group; F – females, M – males). (D) IGF1 levels normalized to SOD1 in E18.5 *Mir483*$^{\text{Pat-KO}}$ livers compared to WT littermate controls (left – western blotting, right – quantification; n=6 per group). (E) IGF2 levels normalized to protein loading (as assessed following Coomasie R-250 dye staining) in E18.5 *Mir483*$^{\text{Pat-KO}}$ livers compared to WT littermate controls (left – western blotting, right – quantification; n=6 per group). The E18.5 *Igf2*^{EpiKO} liver samples were collected from fetuses with epiblast-specific conditional deletion of *Igf2*[76]. (F) Cumulative fractions mRNA fold changes between E18.5 livers of *Mir483*$^{\text{Pat-KO}}$ mutants and WT littermates for conserved putative targets of *miR-483*-3p (left), *miR-483*-5p (middle) or the two up-regulated DEMs *miR-370*-3p and *miR-136*-5p (right). For panels (A) – (E) data are presented as individual values, with averages ± SD; ns – non-significant by two-way ANOVA followed by Šídák's multiple comparisons tests in (A) and (B), by Mann-Whitney tests in (D) and (E) and using two-sided Kolmogorov-Smirnov tests in (F).

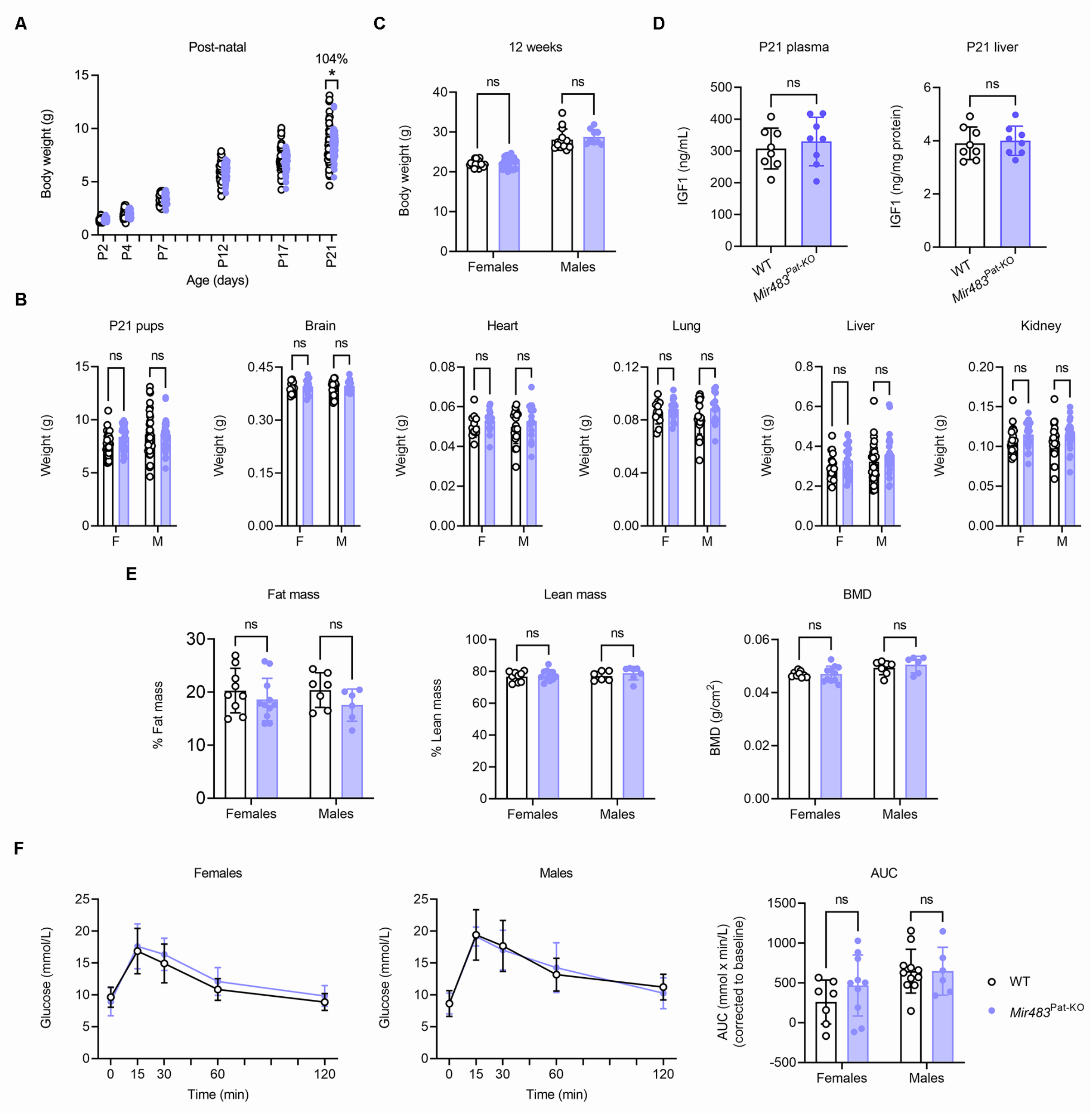

Figure S6. Postnatal phenotyping in the *Mir483*$^{Pat\text{-}KO}$ knockout model. Related to Figure 2.

(A) Post-natal growth kinetics from post-partum day 2 (P2), until weaning (P21) (n=11-20 litters at each developmental stage). (B) Total body (n=33-40 per group) and organ weights (n=12-29 per group) at post-natal day 21 (P21). (C) Body weights in 12 weeks-old knockouts and wild-type littermates (n=9-31 per group). (D) IGF1 protein levels measured at P21 by ELISA in plasma (left) or liver (right) of *Mir483*$^{Pat\text{-}KO}$ mutants and WT littermate controls (n=8 per group). For liver, IGF1 levels were normalized against the total protein content measured by a BCA protein assay. (E) Body composition (% fat mass, % lean mass and bone mineral density – BMD) measured by dual energy x-ray absorptiometry (DEXA) at the age of 12 weeks (n=6-11 per group). (F) Glucose tolerance tests with glucose administered by intra-peritoneal injections (ipGTTs) after overnight fasting in females

(n=7-10/genotype) and males (n=6-11/genotype). First two panels show changes in blood glucose concentrations (y-axis), from basal pre-treatment values, with time (x-axis), after glucose administration. The graph on the far right shows area under curve (AUC) calculated during ipGTTs using the trapezoid rule and normalised to basal glucose levels. Data are presented as individual values, with averages ± SD in (A – E) and (F) (far-right graph), and averages ± SD in (F) (first two graphs on the left) and % values indicate ratios *Mir483*$^{Pat\text{-}KO}$/WT; ns – non-significant, * P<0.05 by a mixed effects model in (A), two-way ANOVA followed by Šídák's multiple comparisons tests in (B), (C), (E) and (F) (far-right graph), and unpaired *t*-tests with Welch's correction in (D).

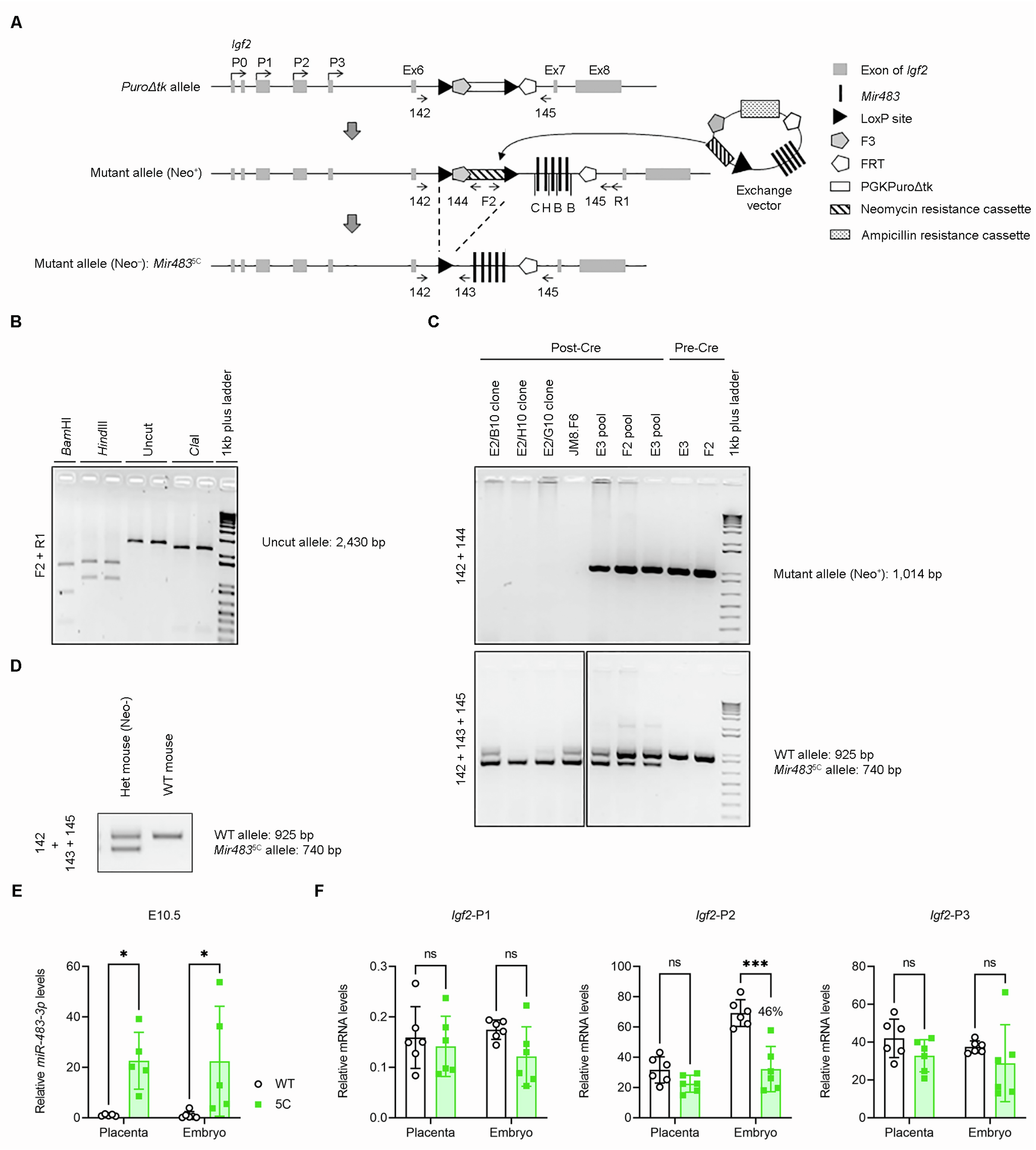

Figure S7. Generation of *Mir483*^{5C} (5C) mice and down-regulation of *Igf2*-P2 isoform in *Mir483*^{5C} embryos, but not placentae. Related to Figure 3.

(A) Mice expressing 5 copies of *Mir483* from the endogenous locus were generated by replacement of the PGKPuroΔtk allele of the *Mir483*^{KO} ES cell line (verified for correct and unique integration by Southern blotting as shown in Figure S6) with a neomycin resistance cassette and 5 copies of *Mir483* using RMCE (recombinase-mediated cassette exchange). The diagram shows the PuroΔtk allele and exchange vector, the (Neo^{+}) allele after RMCE, and the allele after *in vitro Cre* deletion (Neo^{-}). Genotyping primers are depicted by arrows. Selected restriction sites are indicated by C (*Cla*I), E

(*Eco*RI), H (*Hind*III) or B (*Bam*HI). Genomic features are not drawn to scale and are for representation purposes only. (B) PCR using primers F2 and R1 generated a 2,430 bp product indicating that RMCE had occurred and the five copies of *Mir483* were present. The identity of the PCR product was confirmed by digestion with *Bam*HI, *Hind*III or *Cla*I. (C) *In vitro Cre* deletion in ES cell clones was determined by PCR (using the primers indicated in the figure) before (Pre-Cre) and after (Post-Cre) transfection with a plasmid encoding a *Cre* recombinase. DNA pools of ESC clones and parental JM8.F6 ESC DNA were also amplified. (D) Mice were then routinely genotyped by PCR, using primers 142, 143 and 145. (E) Relative expression of *miR-483-3p* measured by RT-qPCR in whole embryo lysates at E10.5 (n=5-6 samples/group). Levels of *miR-483-3p* were normalized against the geometrical mean of *Snord70*/*snoRNA234*, *Snord68*/*snoRNA202*, and are presented relative to the wild-type (WT) levels, arbitrarily set to 1. (F) Relative expression of *Igf2* isoforms at E11.5 measured by RT-qPCR levels in placenta and embryo (n=6 samples per group). For panels (E) and (F) levels of *Igf2* transcripts were normalized against the geometrical mean of *Gapdh*, Sdha and *Pmm1*. Data are presented as individual values, with averages ± SD and % indicate ratios 5C/WT; ns – non-significant, * $P<0.05$ and *** $P<0.001$ by two-way ANOVA followed by Šídák's multiple comparisons tests.

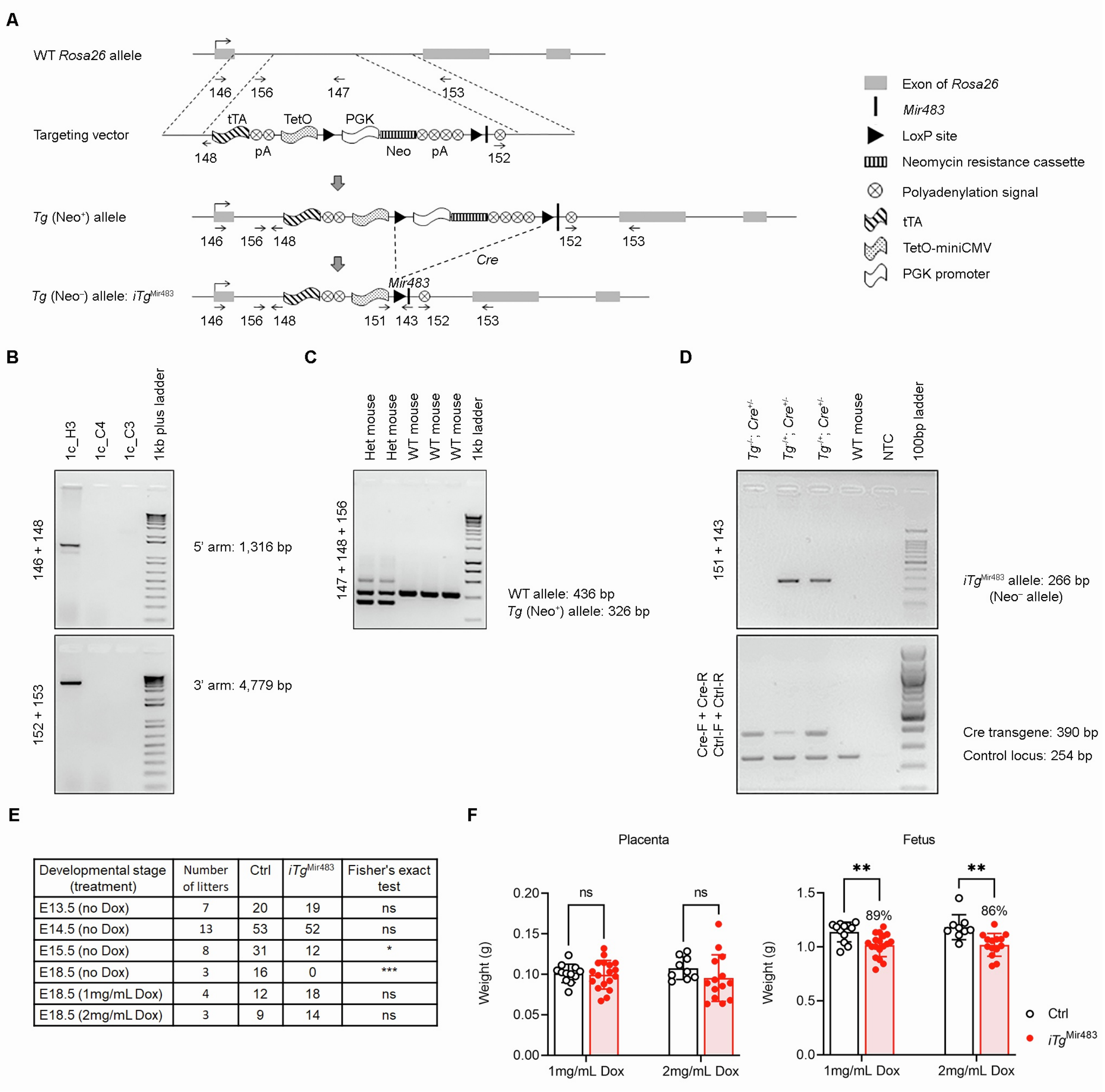

Figure S8. Generation of TET-OFF inducible transgenic mice with an additional copy of *Mir483* inserted at the *Rosa26* locus (*iTg*^{Mir483}), with total rescue of *iTg*^{Mir483} lethality and partial rescue of fetal growth restriction by doxycycline administration. Related to Figure 4.

(A) Diagram showing the wild-type *Rosa26* locus, the targeting vector containing a tetracycline-controlled transactivator (tTA), transgenic (Tg) (Neo^{+}) allele after homologous recombination in ES cells and the Tg allele after *Cre* deletion (Neo^{-}). Genotyping primers are shown by arrows. Genomic features are not drawn to scale and are for representation purposes only. (B) ES cell clones were screened by PCR; homologous recombination of the 5' arm and 3' arm was assayed using primers 146+148 and 152+153, respectively. The gel image depicts one targeted clone (1c_H3) and two non-targeted clones. (C) Mice were then routinely genotyped using primers 147, 148 and 156. (D) Males heterozygous for the Neo^{+} transgene were mated with female mice homozygous for a *CMV-Cre*

transgene, to generate Neo^{-} progeny (*iTg*^{Mir483}), which were genotyped using primers 151 and 143 (across the deleted region) and Cre-F, Cre-R, Ctrl-F and Ctrl-R (to detect the presence of *Cre*), NTC – no template negative control. (E) Maternal doxycycline administration in drinking water (Dox, 1mg/mL or 2 mg/mL) throughout gestation rescues the post-mid-gestation lethality of *iTg*^{Mir483} conceptuses. The table shows the distribution of live conceptuses per genotype identified at various developmental time-points during gestation. (F) Placenta and fetal weights at E18.5 upon maternal administration of Dox (n=9-18/group). Data in panel (F) are presented as individual values, with averages ± SD in and % indicate ratios *iTg*^{Mir483}/Ctrl; ns – non-significant, * P<0.05, ** P<0.01 and *** P<0.001 by Fisher's exact tests in (E) or two-way ANOVA followed by Šídák's multiple comparisons tests in (F).

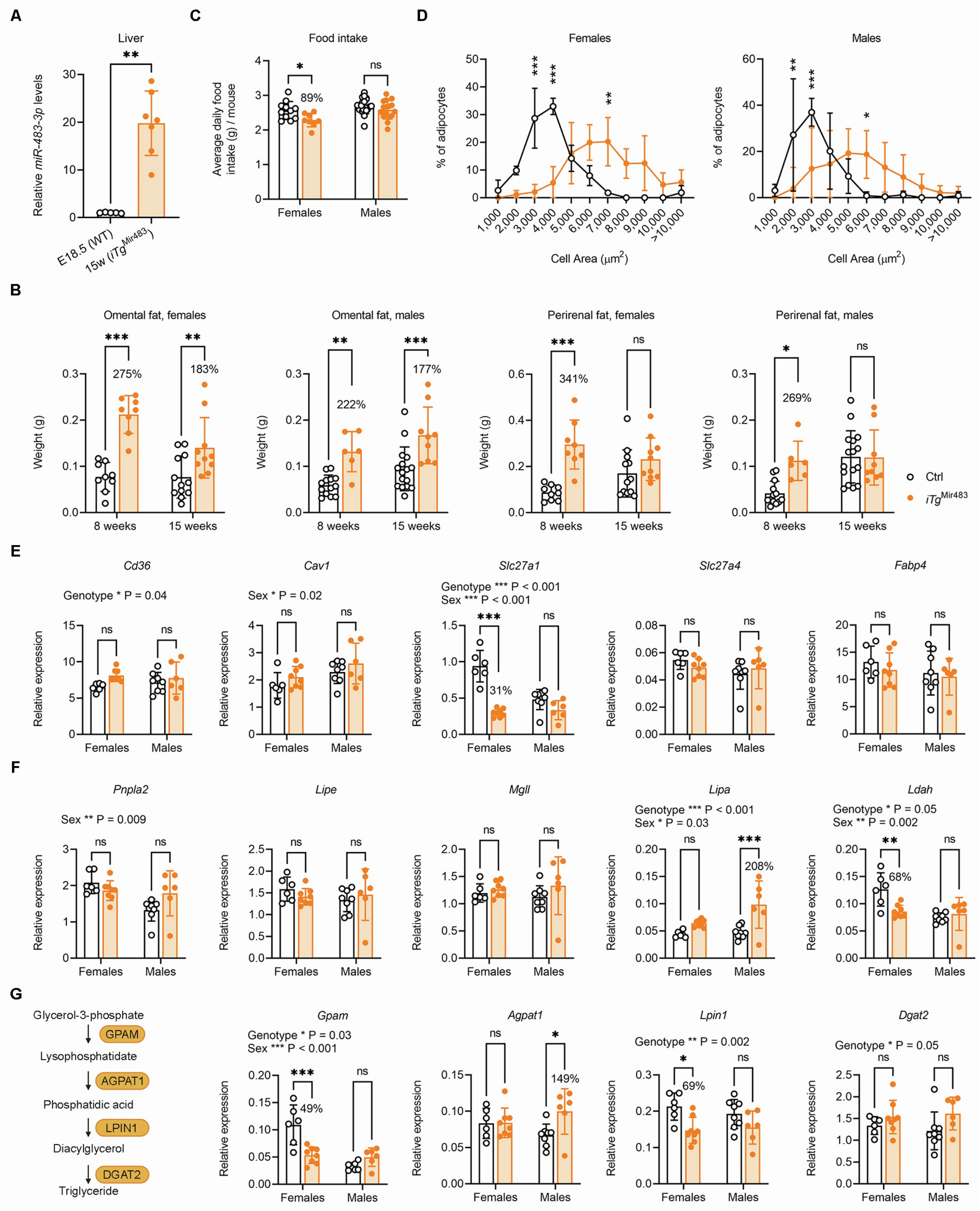

Figure S9. Postnatal phenotyping the *iTg*^{Mir483} mouse model. Related to Figure 5.

(A) Relative levels of *miR-483*-3p measured by RT-qPCR in liver of 15 week-old *iTG*^{Mir483} mutants compared to E18.5 wild-type controls. Levels of *miR-483-3p* were normalized against the geometrical mean of *Snord70*/*snoRNA234* and *Snord68*/*snoRNA202* (n=5-7 samples/group). (B) Omental fat pads are significantly heavier in week 8 (W8) and W15 in *iTG*^{Mir483} adults compared to

age-matched controls, while peri-renal fat pads are significantly heavier only at W8 (n=6-16 per group). (C) Average daily food intake, measured between W3 and W4 is significantly lower in *iTG*^{Mir483} females compared to age-matched controls, with no significant differences between the two genotypes in males (n=8-20 per group). (D) Distribution of adipocyte cell area isolated from the gonadal fat pad of W8 *iTG*^{Mir483} and age-matched controls indicates larger adipocytes in mutants of both sexes (n=2-10 per group). (E) Expression patterns of genes encoding lipid transporters are largely unchanged in adipocytes isolated from gonadal fat of W8 *iTG*^{Mir483} and age-matched controls, with the notable expression of *Slc27a1* (also known as *Fatp1*) that is significantly down-regulated in mutant females only (n=6-8 per group). (F) Expression patterns of genes encoding three major lipases (*Pnlpa2*, also known as *Atgl*; *Lipe*, also known as *Hsl*; *Mgll*, also known as *Mgl*) are unchanged in adipocytes isolated from gonadal fat of W8 *iTG*^{Mir483} and age-matched controls. Significant differences were observed for two minor lipases: *Lipa*, upregulated in males, and *Ldah*, down-regulated in females (n=6-8 per group). (G) Left: diagram depicting the steps involved in the conversion of glycerol-3-phosphate (G3P) into triglycerides (TG). Right: expression patterns of genes encoding enzymes implicated in the synthesis of TG in adipocytes isolated from gonadal fat of W8 *iTG*^{Mir483} and age-matched controls (n=6-8 per group). Data are presented as individual values, with averages ± SD in (A), (B), (C), (E) and (G), or averages ± SD in (D) and % indicate ratios *iTg*^{Mir483}/Ctrl; ns – non-significant, * *P*<0.05, ** *P*<0.01 and *** *P*<0.001 by a Mann-Whitney test in (A), two-way ANOVA followed by Šídák's multiple comparisons tests in panels (B) – (G). For panels (E), (F) and (G) the effects of genotype and sex identified by two-way ANOVA tests are indicated above the graphs. None of the genes shown in these panels are putative direct targets for *Mir483* regulation, with exception of *Lpin1.*

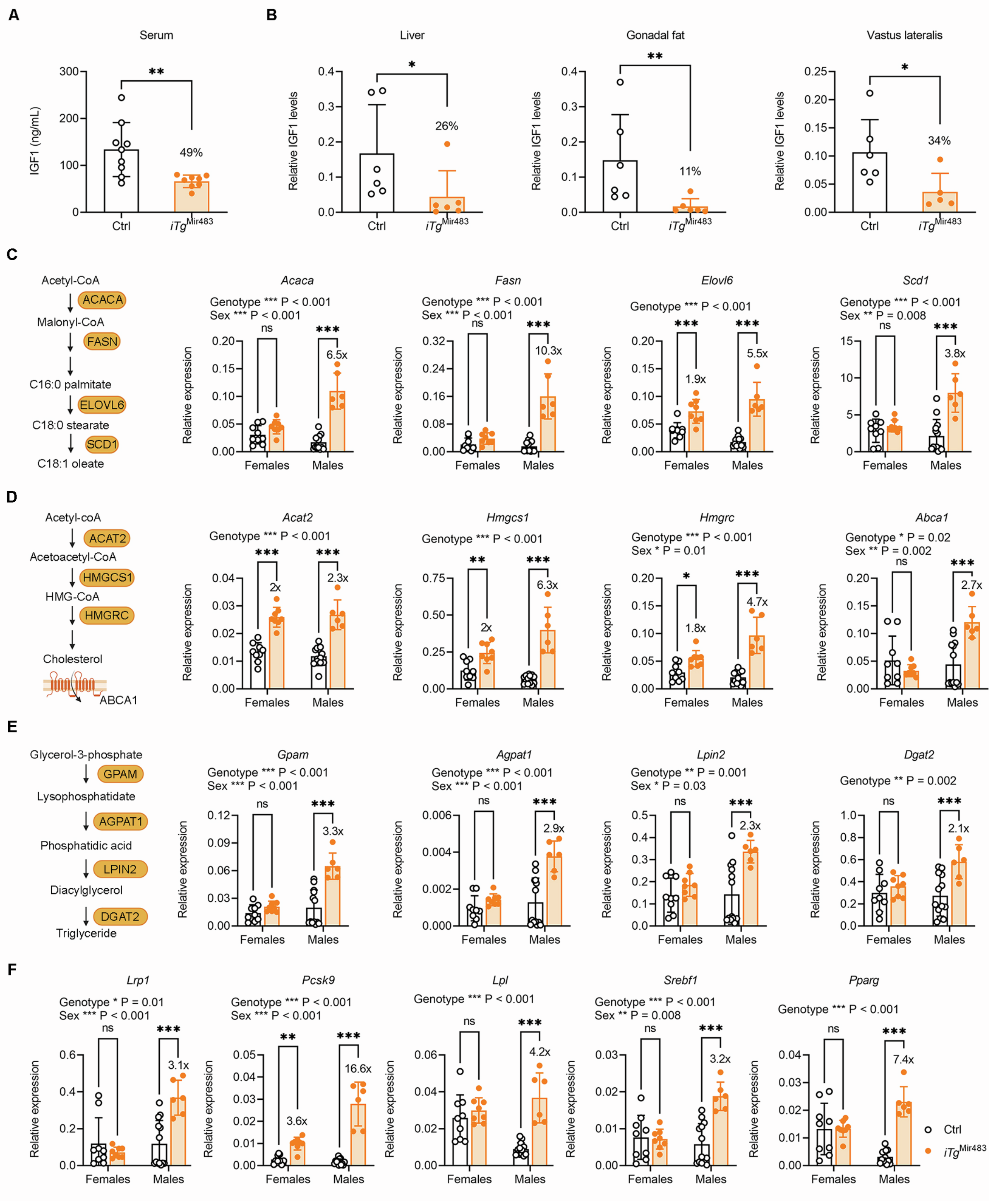

Figure S10. IGF1 protein levels and mRNA levels of genes that regulate lipid production or trafficking in the liver of the *iTg*^{Mir483} mouse model. Related to Figure 6.

(A) IGF1 protein levels measured by ELISA in serum of W3 *iTG*^{Mir483} and age-matched controls (n=8-9 per group). (B) Relative protein levels of mature IGF1 (7.5 kDa) measured by western blot analyses in protein lysates of three organs collected from W15 males of *iTG*^{Mir483} and age-matched controls (n=5-6 per group). Levels of IGF1 were normalized against Coomassie staining in the liver and

gonadal fat, and SOD1 in the *vastus lateralis*, used as internal controls for protein loading. (C) mRNA levels of genes encoding key enzymes involved in free fatty acid (FFA) synthesis in the livers of W8 iTG^{Mir483} adults compared to age-matched controls (n=6-13 per group). (D) Relative mRNA levels of genes encoding key enzymes involved in cholesterol synthesis in the livers of W8 iTG^{Mir483} adults compared to age-matched controls (n=6-13 per group). (E) Relative mRNA levels of genes encoding key enzymes involved in triglyceride synthesis in the livers of W8 iTG^{Mir483} adults compared to age-matched controls (n=6-13 per group). (F) Expression patterns of genes encoding proteins implicated in lipoprotein turnover (*Lrp1*, *Pcsk9* and *Lpl*) and key transcriptional factors that regulate lipid synthesis (*Sreb1*, *Pparg*) in livers of W8 iTG^{Mir483} and age-matched controls (n=6-13 per group). Data are presented as individual values, with averages ± SD and % or "x" indicate ratios/fold changes iTg^{Mir483}/Ctrl; ns – non-significant, * P<0.05, ** P<0.01 and *** P<0.001 by an unpaired *t*-test with Welch's correction in (A) Mann-Whitney tests in (B) and two-way ANOVA followed by Šídák's multiple comparisons tests in panel (C) – (F). For panels (C) – (F), the effects of genotype and sex identified by two-way ANOVA tests are indicated above the graphs. None of the genes shown in these panels are putative direct targets for *Mir483* regulation.

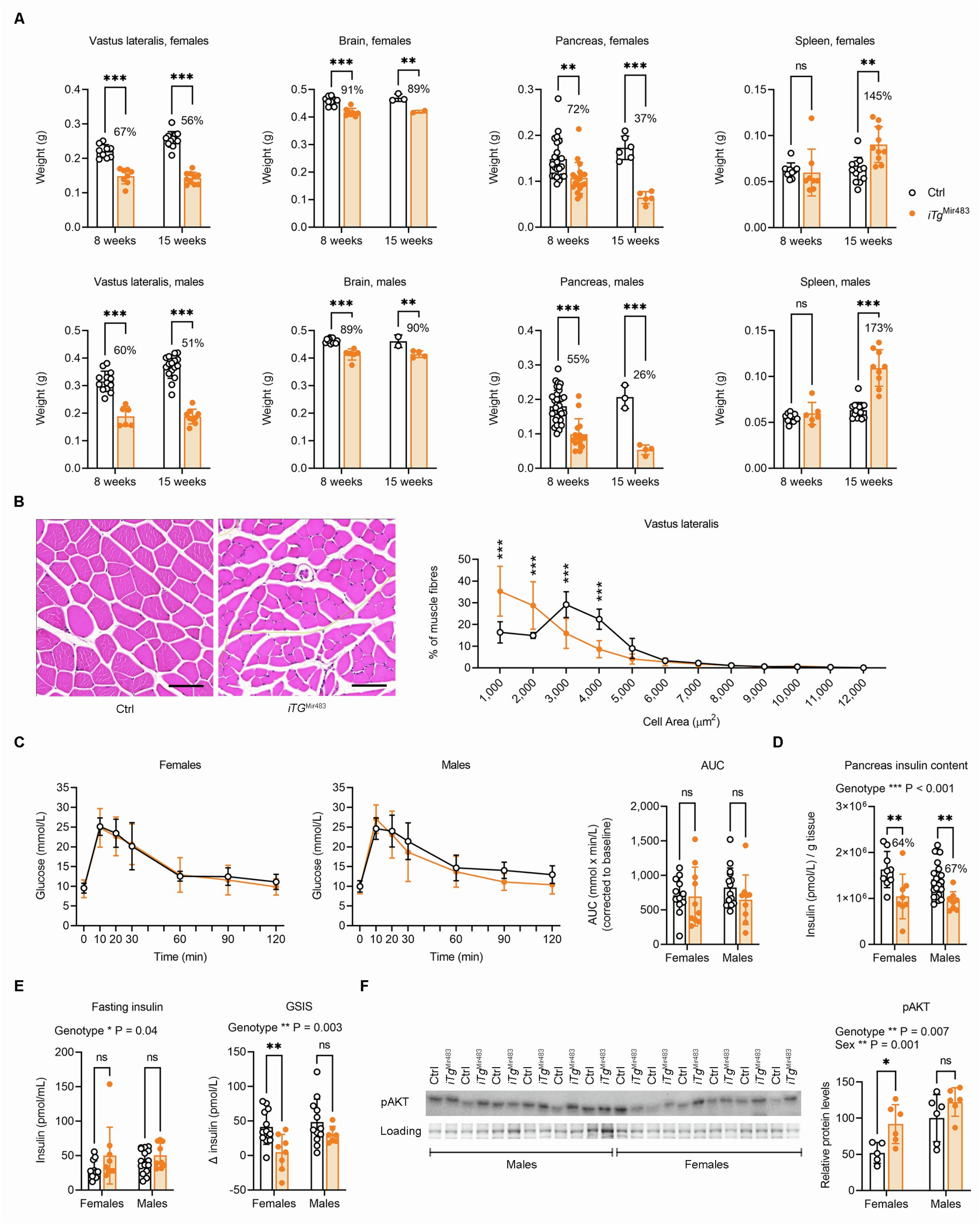

Figure S11. Organ size, muscle morphology and glucose metabolism assessment in the *iTg*Mir483 mouse model. Related to Figure 6.

(A) Organ weights in W8 and W15 *iTG*Mir483 adults compared to age-matched controls (n=2-16 per group). The *vastus lateralis* and pancreas are disproportionally smaller; the brain is proportionally smaller and the spleen is disproportionally bigger, but only at W15. (B) The skeletal muscle fibres are

smaller in the *vastus lateralis* of W15 *iTg*^{Mir483} mutant males compared to age-matched controls (representative H&E stained sections – left, and distribution of muscle fibre area – right; n=5 samples per group, scale bars are 100 µm). (C) Glucose tolerance tests in W13 *iTg*^{Mir483} mutants and age-matched controls, with glucose administered by oral gavage (OGTTs) after six hours fasting performed in females (n=10-12/genotype) and males (n=9-15/genotype). First two panels show changes in blood glucose concentrations (y-axis), from basal pre-treatment values, with time (x-axis), after glucose administration. The graph on the far right shows area under curve (AUC) calculated during OGTTs using the trapezoid rule and normalised to basal glucose levels. (D) Total pancreas insulin content in W18 *iTg*^{Mir483} mutants and age-matched controls after overnight fasting (n=8-22 per group). (E) Left: insulin levels measured in plasma in W13 *iTg*^{Mir483} mutants and age-matched controls, after six hours fasting and prior to the start of the OGTT (n=9-16 samples per group). Right: glucose-stimulated insulin secretion (GSIS) measured in plasma at minute 20 during OGTT (n=7-12 samples per group). (F) pAKT levels normalized to protein loading (as assessed following Coomasie R-250 dye staining) in the gonadal fat of W8 *iTG*^{Mir483} adults compared to age-matched controls (left – western blotting, right – quantification; n=6 per group). Data are presented as individual values, with averages ± SD in (A), (C) (far right), (D), (E) and (F) (right) or as averages ± SD in (B) and (C) (left side), and % indicate ratios *iTg*^{Mir483}/Ctrl; ns – non-significant, ** $P<0.01$ and *** $P<0.001$ by two-way ANOVA followed by Šídák's multiple comparisons tests in (A)-(F). For panels (D)-(F), the effects of genotype or sex identified by two-way ANOVA tests are indicated above the graphs.

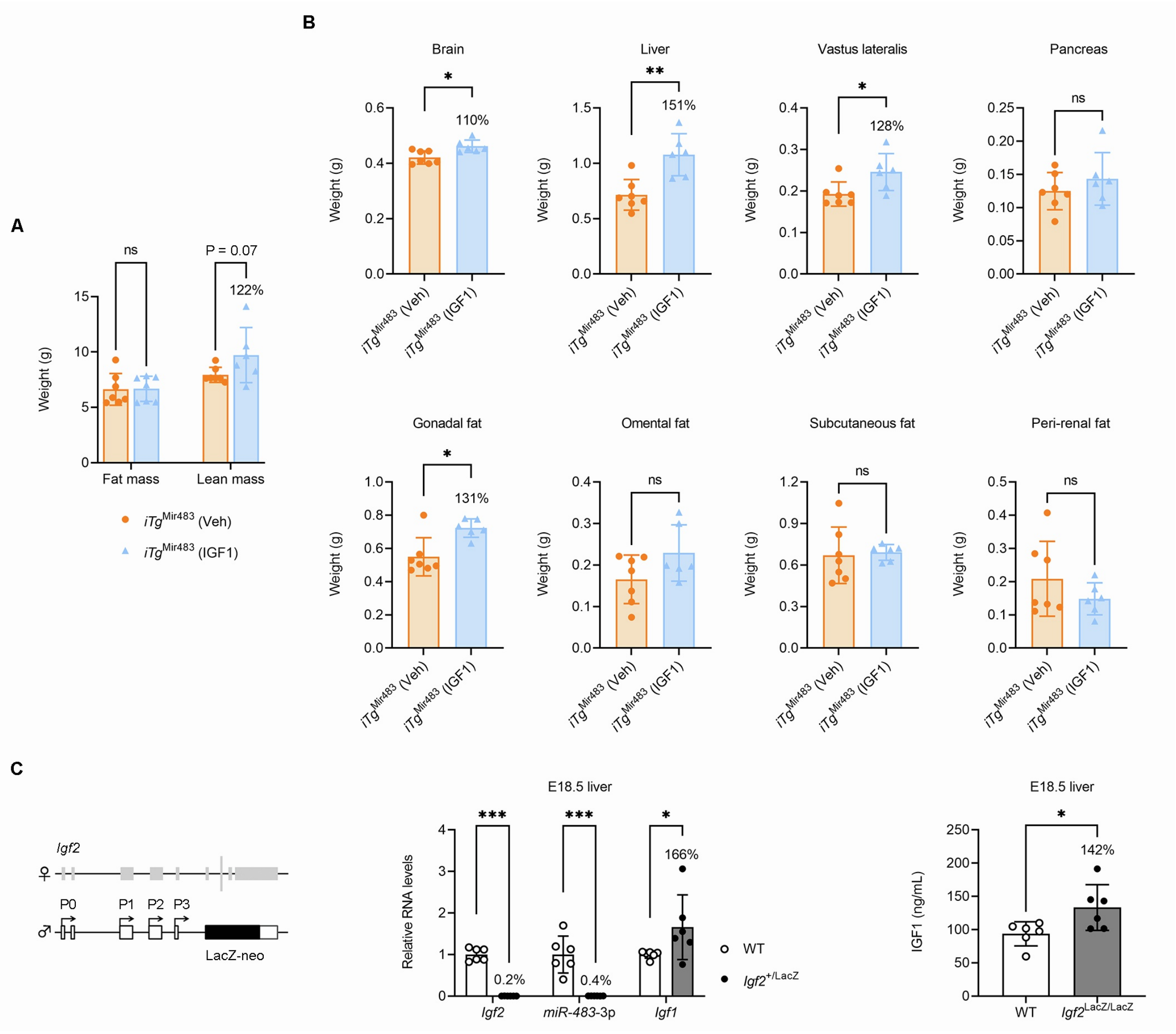

Figure S12. Impact of IGF1 infusion on body composition in the *iTg*$^{\text{Mir483}}$ mouse model and additional *in vivo* evidence for *Igf1* as target of *miR-483*. Related to Figure 7.

(A) Body composition measured by TD-NMR in W8 *iTg*$^{\text{Mir483}}$ male mice exposed to vehicle or IGF1 via minipumps (n=6-7 per group). (B) Organ weights in W8 *iTG*$^{\text{Mir483}}$ male mice exposed to vehicle or IGF1 via minipumps (n=6-7 per group). (C) Left: schematic representation of the *Igf2*$^{+/\text{LacZ}}$ model in which the coding exons 4-6 of *Igf2* and *Mir483* are replaced by a LacZ cassette. Genomic features are not drawn to scale and are for representation purposes only. Middle: relative RNA levels for *Igf2*, *miR-483-3p* and *Igf1* in the liver of E18.5 *Igf2*$^{+/\text{LacZ}}$ mutants and WT littermate controls (data was normalized against the geometrical means of *Ppia* and *Gapdh* for *Igf2* and *Igf1*, and against *Snord70*/*snoRNA234* for *miR-483*-3p; n=6 samples per group). Right: measurement of IGF1 protein by ELISA in the liver of E18.5 *Igf2*$^{\text{LacZ/LacZ}}$ mutants and WT littermate controls (n=6 per group). Data are presented as individual values with averages ± SD and % values indicate ratios *iTG*$^{\text{Mir483}}$ (IGF1)/ *iTG*$^{\text{Mir483}}$ (Veh) in (A) and (B) or *Igf2*$^{+/\text{LacZ}}$/WT or *Igf2*$^{\text{LacZ/LacZ}}$/WT in c; ns – non-significant, * P<0.05, **

P<0.01 and *** *P*<0.001 by two-way ANOVA followed by Šídák's multiple comparisons tests in (A) and (C) (middle), or Mann-Whitney tests in (B) and (C) (right).

Table S5. Overview of developmental defects observed by HREM (High-Resolution Episcopic Microscopy) in *iTg*^{Mir483} embryos at E14.5 (n=6). Related to Figure 4.

Organ/system	Specific defect	Frequency
Cardio-vascular	Atrium and AV junction defects	100%
	Double outlet right ventricle (DORV) with associated ventricle septum defect	
	Malformations of intrathoracic arteries (interruption of aortic arch, aortic coarctation, right sided aortic arch, left sided lusoria artery, connections between subclavian artery and pulmonary trunk)	
	Abnormalities of head arteries	
	Abnormalities of the ductus venosus and portal vein	
Urogenital tract	Abnormal remodeling of the metanephrotic tissue and its ducts	83%
	Abnormalities of kidneys or ureters (abnormal pelvis renalis to absent ureter and additional ureteral buds)	
	Malformations of the Wolff and Müller ducts	
Skeleton	Absent or abnormal acromion with abnormal acromioclavicular joints	83%
	Abnormal otic vesicles	
	Thoracoschisis	
	Abnormal nasal cavities and associated head bones	
	Abnormal tail morphology	
Thymus	Abnormal topology of the thymus	67%
Liver/bile ducts	Enlarged sinusoidal spaces	67%
	Additional ducts branching from the cystic duct to enter the liver	
Eye and eye muscle	Eyes with missing lenses	67%
	Eye muscle abnormalities	
	Retro-lental blood	
Nervous system	Brain defects	50%
	Holoprosencephaly	
	Smaller superior cervical ganglion	
Thyroid	Unilateral absence of the thyroid gland lobe	33%

Table S7. Primers and TaqMan probes used for genotyping mouse strains by PCR and RT-qPCR

Primers used for genotyping mouse strains by PCR					
Mouse strain	Primer	Sequence (5'-3')	Primer	Sequence (5'-3')	Amplicon (bp)
Igf2$^{\Delta(P1\text{-}P3)}$	F	ATGTCTCCAATCCTTGAACACTG	R1<br>R2	GCAGTGGGAGAAATCAGAACC<br>GCTTTTTTAGTGGTGGGAGGC	WT – 254<br>Floxed – 509<br>Del – 406
H19$^{\Delta13}$	F	TGCCACAGAGGAA-GAAACCAG	R1<br>R2	AGTCATAGCCGAATAGCC<br>TTCAGTCACTTCCCTCAGCCTC	Δ13 – 895<br>WT – 494
Mir483^{KO}	F1	TACCTGCCTGTGAACTGCTCTG	R1	ATCTGGTGCCTCCTGTCTGGTA	WT – 440<br>KO – 457
Mir483^{5C}	142	CACGCTTCAGTTTGTCTGTTCG	143<br>145	AAGAATCGATACCGTCGACCTC<br>CTGGAGTGGTTTGGAAAACAGG	WT – 925<br>5C – 740
iTg^{Mir483}	156<br><br>151	TCCCAAAGTCGCTCTGAGTT<br><br>AGGGAGTGGTAAACTCGACC	147<br>148<br>143	GGCGGATCACAAGCAATAAT<br>GAAAGACCGCGAAGAGTTTG<br>AAGAATCGATACCGTCGACCTC	WT – 436<br>Neo^{+} – 326<br>Neo^{-} – 266
CMV-Cre	Cre-F<br>Ctrl-F	CGAGTGATGAGGTTCGCAAG<br>ATGTCTCCAATCCTTGAACACTG	Cre-R<br>Ctrl-R	TGAGTGAACGAACCTGGTCG<br>GCAGTGGGAGAAATCAGAACC	Cre – 340<br>WT – 254
Igf2^{KO}	F	TTACAGTTCAAAGCCACCACG	R1<br>R2	GCCAAAGAGATGAGAAGCACC<br>GCCAAACACAGTAAAAAGAAAT GC	WT – 324<br>Floxed – 449<br>Del – 384
Igf2^{LacZ}	F	TCCTCAAGGGTTTCTTACAGTTC	R1<br>R2	CCTCGACTAAACACATGTAAAGC<br>GACAAACTGAAGCGTGTCAAC	WT – 420<br>LacZ – 807
Primers and TaqMan probes used for RT-qPCR					
Gene	Primer	Sequence (5'-3')	Primer	Sequence (5'-3')	Amplicon (bp)
miR-483-3p	mmu481853_mir (ThermoFisher Scientific #A25576)				
miR-483-5p	mmu481180_mir (ThermoFisher Scientific #A25576)				
Snord70/ snoRNA234	001234 (ThermoFisher Scientific #4427975)				
Snord68/ snoRNA202	001232 (ThermoFisher Scientific #4427975)				
miR-26b	mmu481662_mir (ThermoFisher Scientific # A25576)				
Igf2	F (Ex7)	AGTCCGAGAGGGACGTGTCTA	R (Ex8)	CGGACTGTCTCCAGGTGTCAT	102

Igf2-P0	F (Ex2)	GAGGAAGCTCTGCTGTTTGG	R (Ex6)	CAAAGAGATGAGAAGCACCAAC	92
Igf2-P1	F (Ex3)	GACAAGGGTCTGACTTGGGA	R (Ex3)	CGTAGGAGAAGTGACGAGGC	113
Igf2-P2	F (Ex4)	GTCGACCCTAACCGAGCTG	R (Ex4)	AAGCAGAGGAGAGGATGCAA	103
Igf2-P3	F (Ex5)	TGGACATTAGCTTCTCCTGTGA	R (Ex5)	GCTGGAAGAGGATGAAGACAG	61
Igf1	F	GCTGGTGGATGCTCTTCAGTT	R	CTCATCCACAATGCCTGTCTG	112
Ppia	F	AAGGGTTCCTCCTTTCACAGAA	R	GATGCCAGGACCTGTATGCTT	146
Pmm1	F	ATCCGGGAGAAGTTTGTGGAA	R	GCTGTCTTCATCCAGGCTGTC	144
Hprt	F	CATTATGCCGAGGATTTGGAA	R	CCTTCATGACATCTCGAGCAA	88
Tbp	F	AACAACAGCCTTCCACCTTATG	R	TGTTCTGAATAGGCTGTGGAGT	127
Gapdh	F	ACAACTCACTCAAGATTGTCAGCA	R	ATGGCATGGACTGTGGTCAT	121
Actb	F	GATCAAGATCATTGCTCCTCCTG	R	AGGGTGTAAAACGCAGCTCA	183
Acaca	F	ACGTGCAATCCGATTTGTTGT	R	CCAGCCCACACTGCTTGTA	180
Fasn	F	TGCACCTCACAGGCATCAAT	R	GTCCCACTTGATGTGAGGGG	104
Elovl6	F	ACCCGAACTAGGTGACACGA	R	AGTCATGAACCAACCACCCC	142
Scd1	F	TGGTGAACAGTGCCGCGCAT	R	CGGCACCCAGGGAAACCAGG	90
Acat2	F	TACCTCAGTCGCAGACAGGA	R	TGAAGGAGCCTATAGCGGTG	119
Hmgcs1	F	CTGCTATTCTGTCTACCGCAAA	R	GGAACATCCGAGCTAGAGATTTC	146
Hmgrc	F	GGAATGCCTTGTGATTGGAGTT	R	CTCTAGGACCAGCGACACAC	142
Abca1	F	AGGGCATGTGGGAAGAACTC	R	TGTTCCCAAAACTGGTCATTGC	115
Gpam	F	TGCAACACTGAAATGGAAGGAG	R	ATAACATTCCGCAAACCCAGAG	138
Agpat1	F	AGCTCCAGTGCCAAGTATTTCT	R	GTATTTGACGTGGAGCAGCAG	150
Lpin1	F	GGAGACAACGGAGAAGCATTTT	R	GTTCCTCTTCAGCTGGCTTTC	129
Lpin2	F	CCTGAGGTCCAAGGAGAAAGT	R	GCTTCCCCATTATCACCCAATT	87
Dgat2	F	GCTGCAGGTCATCTCAGTACTA	R	TGCAGAAGGTGTACATGAGGAT	89
Cd36	F	TGCTGGAGCTGTTATTGGTG	R	GGTGCCTGTTTTAACCCAGTT	148
Cav1	F	ATACGTAGACTCCGAGGGACA	R	ACGTCGTCGTTGAGATGCTT	174
Slc27a1	F	TTCTCGTGGGCCAGATCAAC	R	AGCACGTCACCTGAGAGGTA	136
Slc27a4	F	CCAAAGCTGCCATTGTGGTG	R	ATCCCCACGATGTTTCCTGC	136
Fabp4	F	AGCTGGTGGTGGAATGTGTTAT	R	CCTCTTCCTTTGGCTCATGC	76
Pnpla2	F	GGAGGAATGGCCTACTGAACC	R	ATCCTCTTCCTGGGGGACAA	71
Lipe	F	CAAGCCCCAAAAGACCACATC	R	CTTCTTCAAGGTATCTGTGCCC	127
Mgll	F	AGACGGACAGTACCTCTTTTGT	R	ATGTCCAGCCCCTTCAACATAT	135
Lipa	F	AGATAATCATGCGCTGGGGATA	R	AAGATACACAACTGGTCTGGGA	137
Ldah	F	GTAGGCACCTATATGACCCTTC	R	CGATAGTTGGGAAGAGCAGAAA	85
Lrp1	F	GCCCACGTGCTACTGTAACA	R	ATGTGAAGGAGCCATCTGTGTT	128

Pcsk9	F	CTTGGTGAAGATGAGCAGTGAC	R	GGATAATTCGCTCCAGGTTCC	122
Lpl	F	TGTGAAATGCCATGACAAGTCT	R	CACTTTCAAACACCCAAACAAGG	150
Srebf1	F	ACTTTTCCTTAACGTGGGCCT	R	AGCTGGAGCATGTCTTCGAT	153
Pparg	F	TTTAAAAACAAGACTACCCTTTA CTGAAATT	R	AGAGGTCCACAGAGCTGATTCC	95
Ttc36	F	CCTTTGGAGATGTTGTTGGATT	R	CAACTGTGCTTGAGGGAAAACT	80
Arhgdig	F	AGATTGTCAGTGGCCTCAAATG	R	AATTCATACTCCTGGGCTCTGG	112
Lep	F	CCCAAAATGTGCTGCAGATAG	R	CCAGCAGATGGAGGAGGTC	63

Table S8. Primary and secondary antibodies used in Western blotting experiments

Antibody	Dilution	Supplier
Rabbit anti-phospho-AKT (Ser 473)	1:1,000	Cell Signaling Technology (#9271)
Polyclonal Goat anti-Mouse IGF-II	1:2,000	R&D Systems (#AF792)
Polyclonal Goat anti-Mouse IGF-I	1:2,000	R&D Systems (#AF791)
Rabbit polyclonal anti-SOD1	1:1,000	Abcam (#ab183881)
Goat anti-Rabbit IgG (HRP)	1:20,000	Abcam (#ab6721)
Rabbit anti-Goat IgG (HRP)	1:10,000	ThermoFisher Scientific (#31433)
