# Supplementary material for: Overexpression of Igf2-derived Mir483 inhibits Igf1 expression and leads to developmental growth restriction and metabolic dysfunction in mice
Source: Cell Rep. Author manuscript; Available in PMC 2025 Jan 12. (PMC7617298; doi:10.1016/j.celrep.2024.114750)
Supplement: Supplementary Material [file EMS202134-supplement-Supplementary_Material.zip › 1-s2.0-S221112472401101X-mmc7.pdf]

Cell Reports

Article

# Overexpression of *Igf2*-derived *Mir483* inhibits *Igf1* expression and leads to developmental growth restriction and metabolic dysfunction in mice

## Graphical abstract

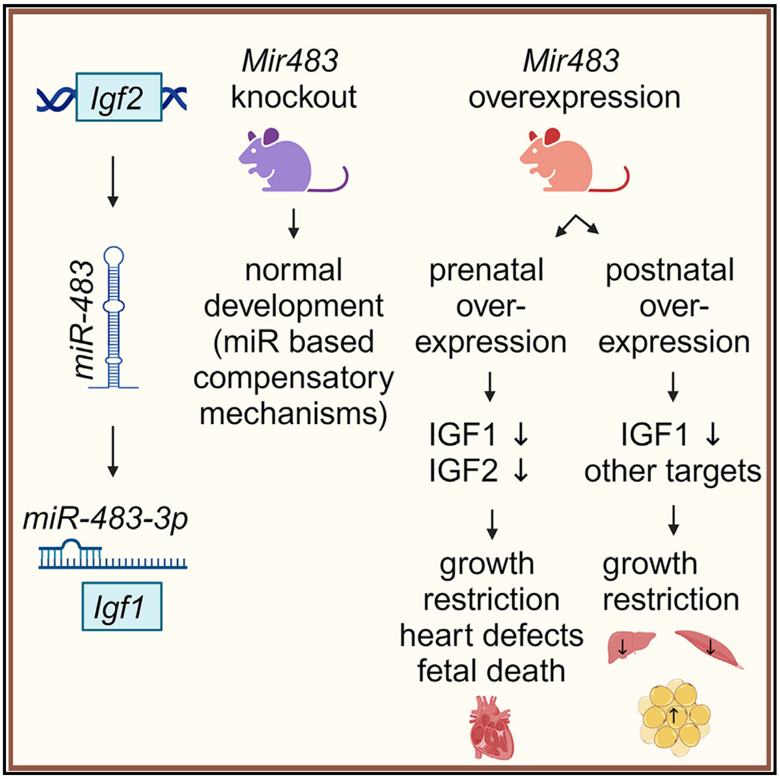


## Highlights

- In the mouse, expression of imprinted *Mir483* is dependent on *Igf2* transcription
- *Mir483* is a developmental growth suppressor and *Igf1* is a major target gene
- Transgenic *Mir483* overexpression *in utero* leads to fetal demise
- Post-natal *Mir483* overexpression induces growth restriction and excessive adiposity

## Authors

Ionel Sandovici, Denise S. Fernandez-Twinn, Niamh Campbell, ..., Martin Bushell, Susan E. Ozanne, Miguel Constância

## Correspondence

jmasmc2@cam.ac.uk

## In brief

Sandovici et al., using loss- and gain-of-function mouse models, dissect the mechanisms that regulate *Mir483* expression and characterize its role in developmental growth and metabolism. They uncover that *Mir483* actions repress IGF signaling, in particular *Igf1*, and function in a manner opposite the growth-promoting host gene *Igf2*.

Sandovici et al., 2024, Cell Reports *43*, 114750
September 24, 2024 © 2024 The Author(s). Published by Elsevier Inc.
https://doi.org/10.1016/j.celrep.2024.114750

CellPress

Cell Reports

CellPress
OPEN ACCESS

Article

# Overexpression of *Igf2*-derived *Mir483* inhibits *Igf1* expression and leads to developmental growth restriction and metabolic dysfunction in mice

Ionel Sandovici,[1,2,3,22] Denise S. Fernandez-Twinn,[1,22] Niamh Campbell,[1,2,22] Wendy N. Cooper,[1,2,12,22] Yoichi Sekita,[1,2,13,22] Ilona Zvetkova,[1,22] David Ferland-McCollough,[4,14] Haydn M. Prosser,[5,15] Lila M. Oyama,[1,6] Lucas C. Pantaleão,[1] Danilo Cimadomo,[2,7,16] Karina Barbosa de Queiroz,[2,17] Cecilia S.K. Cheuk,[2,8] Nicola M. Smith,[1,18] Richard G. Kay,[1] Robin Antrobus,[9,10] Katharina Hoelle,[2] Marcella K.L. Ma,[1] Noel H. Smith,[1,19] Stefan H. Geyer,[11] Lukas F. Reissig,[11] Wolfgang J. Weninger,[11] Kenneth Siddle,[1] Anne E. Willis,[4,20] Brian Y.H. Lam,[1] Martin Bushell,[4,21] Susan E. Ozanne,[1,3] and Miguel Constância[1,2,3,23,*]

[1]Medical Research Council Metabolic Diseases Unit, Institute of Metabolic Science-Metabolic Research Laboratories, University of Cambridge, Cambridge, UK
[2]Department of Obstetrics and Gynaecology and National Institute for Health Research Cambridge Biomedical Research Centre, Cambridge, UK
[3]Centre for Trophoblast Research, Department of Physiology, Development and Neuroscience, University of Cambridge, Cambridge, UK
[4]Medical Research Council Toxicology Unit, University of Leicester, Leicester, UK
[5]The Wellcome Trust Sanger Institute, Genome Campus, Hinxton, UK
[6]Departmento de Fisiologia, Universidade Federal de São Paulo, Escola Paulista de Medicina, São Paulo, Brazil
[7]Laboratory of Developmental Biology, Department of Biology and Biotechnology "Lazzaro Spallanzani," University of Pavia, Pavia, Italy
[8]Nuffield Department of Women's & Reproductive Health, University of Oxford, Oxford, UK
[9]Cambridge Institute for Medical Research, University of Cambridge, Cambridge, UK
[10]Department of Medicine, University of Cambridge, Cambridge, UK
[11]Center for Anatomy and Cell Biology, Division of Anatomy, Medical University of Vienna, Vienna, Austria
[12]Present address: Cancer Research UK Cambridge Centre, Cancer Research UK Cambridge Institute, Li Ka Shing Centre, Cambridge, UK
[13]Present address: Laboratory of Stem Cell Biology, Department of Biosciences, Kitasato University School of Science, Kanagawa, Japan
[14]Present address: Institut de Recherches Cliniques de Montréal, Montreal, QC, Canada
[15]Present address: Cambridge Institute of Therapeutic Immunology and Infectious Disease and Department of Medicine, Jeffrey Cheah Biomedical Centre, University of Cambridge, Cambridge, UK
[16]Present address: IVIRMA Global Research Alliance, Genera, Clinica Valle Giulia, Rome, Italy
[17]Present address: Departamento de Alimentos, Programa de Pós-Graduação em Saúde e Nutrição, Escola de Nutrição, Universidade Federal de Ouro Preto, Ouro Preto, Brazil
[18]Present address: Norwegian Institute of Public Health, Oslo, Norway
[19]Present address: Lonza Biologics, Chesterford Research Park, Saffron Walden, UK
[20]Present address: Medical Research Council Toxicology Unit, University of Cambridge, Cambridge, UK
[21]Present address: Cancer Research UK Beatson Institute, Glasgow, UK, and Institute of Cancer Sciences, University of Glasgow, Glasgow, UK
[22]These authors contributed equally
[23]Lead contact
*Correspondence: jmasmc2@cam.ac.uk
https://doi.org/10.1016/j.celrep.2024.114750

## SUMMARY

*Mir483* is a conserved and highly expressed microRNA in placental mammals, embedded within the *Igf2* gene. Its expression is dysregulated in a number of human diseases, including metabolic disorders and certain cancers. Here, we investigate the developmental regulation and function of *Mir483 in vivo*. We find that *Mir483* expression is dependent on *Igf2* transcription and the regulation of the *Igf2/H19* imprinting control region. Transgenic *Mir483* overexpression *in utero* causes fetal, but not placental, growth restriction through insulin-like growth factor 1 (IGF1) and IGF2 and also causes cardiovascular defects leading to fetal death. Overexpression of *Mir483* post-natally results in growth stunting through IGF1 repression, increased hepatic lipid production, and excessive adiposity. IGF1 infusion rescues the post-natal growth restriction. Our findings provide insights into the function of *Mir483* as a growth suppressor and metabolic regulator and suggest that it evolved within the *INS-IGF2-H19* transcriptional region to limit excessive tissue growth through repression of IGF signaling.


This is an open access article under the CC BY license (http://creativecommons.org/licenses/by/4.0/).

## INTRODUCTION

MicroRNAs (miRs) are endogenous non-coding small RNAs that modulate gene expression at the post-transcriptional level and are critically involved in many cellular processes.[1–3] Aberrant expression of miRs is associated with a number of diseases, in particular various cancers. For this reason, miRs are increasingly used as potential biomarkers of disease[4,5] and therapeutic agents.[6]

Imprinted domains, which are chromosomal regions containing clusters of genes expressed preferentially from one parental allele, transcribe hundreds of small non-coding RNAs, including small nucleolar RNAs (snoRNAs) and miRs.[7] It is estimated that ~7% of known human miRs are encoded by imprinted domains.[8] Most imprinted miRs are generated from three evolutionarily different chromosomal domains as large repetitive arrays.[9,10] They show tissue-specific expression, with marked or exclusive expression in placenta[11] and with multiple placental roles. However, there are a few imprinted miRs produced by a single gene locus,[9,11] including the imprinted *IGF2/H19* domain. This domain is part of the so-called "imprinting growth" chromosomal region on human 11p15 and distal mouse chromosome 7. Imprinted genes in this cluster have key functions in placental development and fetal growth. De-regulation of imprinted expression of a subset of these genes is causative of two human growth syndromes (BWS [Beckwith-Wiedemann syndrome] and SRS [Silver-Russell syndrome][12]), and a variety of cancers also show altered expression linked to tumor growth.[13]

The *Igf2/H19* domain contains two isolated miRs that are highly expressed in placental and fetal tissues: *Mir483* and *Mir675* (mouse nomenclature[14]) (Figure 1A). The paternal expression of *Igf2* and the maternal expression of *H19* are mainly under the control of a differentially DNA methylated region located upstream of *H19*, called imprinting control region 1 (ICR1).[7,15] Work in mice suggests that the controlled release of *Mir675* from the *H19* gene is important to limit the growth of the placenta specifically in late gestation.[16] The physiological roles of *Mir483* in a developmental context are unknown.

Most of the published studies regarding *Mir483* are related to cancer, with the majority of the reports classifying *MIR483* (human nomenclature[14]) as an onco-miR,[17,18] though some studies provide evidence for an oncosuppressor action in certain contexts.[19,20] *Mir483* has been associated with a number of biological processes, such as cellular differentiation,[21] proliferation,[22] survival,[23] melatonin synthesis,[24] insulin production,[25,26] and vascular homeostasis.[27] Importantly, *Mir483* has been shown to be regulated by environmental cues such as diet[28] and temperature.[29]

In this study, we investigated the mechanisms that regulate the expression and imprinting of *Mir483* and addressed functional roles during *in utero* and post-natal development using loss-of-function and gain-of-function *Mir483* transgenic mouse models. Our data suggest that a main role for the *Igf2*-encoded *Mir483* is growth control, mediated by the regulation of insulin-like growth factor 1 (IGF1) levels.

## RESULTS

### The mouse *Mir483* is under the regulatory control of *Igf2* and ICR1 and is not a self-regulating miR

*Mir483* is embedded within intron 6 of the *Igf2* gene (Figure 1A). The primary sequence and predicted *mir-483* (nomenclature for stem-loop[14]) secondary structure are highly conserved in eutherian mammals and marsupials, but not in monotremes (Figures S1A–S1C). The expression of mouse *miR-483-3p* (nomenclature for mature miR[14]) strongly positively correlated with that of the host *Igf2* gene across a range of fetal tissues (Figure 1B). Both *Igf2* and *miR-483-3p* levels decreased in post-natal life, with low expression observed in adult liver (Figure S2A) and other adult organs (Figure S2B). Promoter-specific transcript analysis showed that promoter 2 (P2) was the *Igf2* promoter whose activity had the strongest correlation with *miR-483-3p* levels in a range of tissues and across developmental time points (Figure S2B).

The expression associations between the *Igf2* and the *Mir483* transcripts suggested that *Mir483* shares regulatory elements with the host gene. To test this hypothesis, we first generated and analyzed mice carrying a deletion of the *Igf2* upstream transcriptional unit, i.e., main promoters P1, P2, and P3 and the associated 5′ UTR exons (referred to as $Igf2^{+/\Delta(P1\text{-}P3)}$) (Figure S3). We observed that *miR-483-3p* transcription was abolished in mice carrying this deletion on the paternal allele, in tandem with the loss of *Igf2* expression (Figure 1C). Next, we investigated if ICR1—located upstream of the *H19* gene (Figure 1A)—also controlled the expression of *Mir483*. A maternally inherited deletion of the ICR1/*H19* gene (referred to as $H19^{\Delta13/+}$), an *in vivo* model of *Igf2* loss of imprinting in offspring,[30] led to the reactivation of the maternally silent *Igf2* promoters (Figure 1D). *MiR-483* levels were increased in parallel at relative levels similar to those observed for *Igf2* (Figure 1D).

Expression of the human *MIR483* has been reported to be driven by an upstream miR-specific promoter.[31] Analysis of the human *MIR483* promoter sequence against the mouse showed little evidence of similarity, thus arguing against *Mir483* functioning as self-regulating in the mouse (Figure S1D). We next tested for conservation in mice of the previously reported *miR-483-3p* seed matches in the human *IGF2* locus.[18] *In silico* analysis revealed putative sites for *miR-483-3p* regulation at the 3′ and 5′ mouse *Igf2* UTRs, with a predicted binding site mapping to the mouse P2 promoter region (exon 4 5′ UTR), equivalent to the human P3 promoter region (exon 6 5′ UTR), which was conserved in eutherian mammals and the marsupial wallaby (Figure S1E), but not in the opossum (Figure S1F).

### Constitutive *Mir483* knockout has no obvious phenotypic consequences but shows altered miR and gene expression profiles in fetal liver

Embryonic stem cells carrying a *Cre*-mediated deletion of *Mir483* were used to generate a *Mir483*-knockout mouse (referred to as $Mir483^{Pat\text{-}KO}$; see Figures 2A and S4). Paternal transmission of the deletion did not alter *Igf2* levels (Figures 2B, S5A, and S5B) but caused >95% reduction in levels of *miR-483-3p* (Figure 2B), demonstrating that *Mir483* is regulated by genomic imprinting. There was no impact observed on the

CellPress
OPEN ACCESS

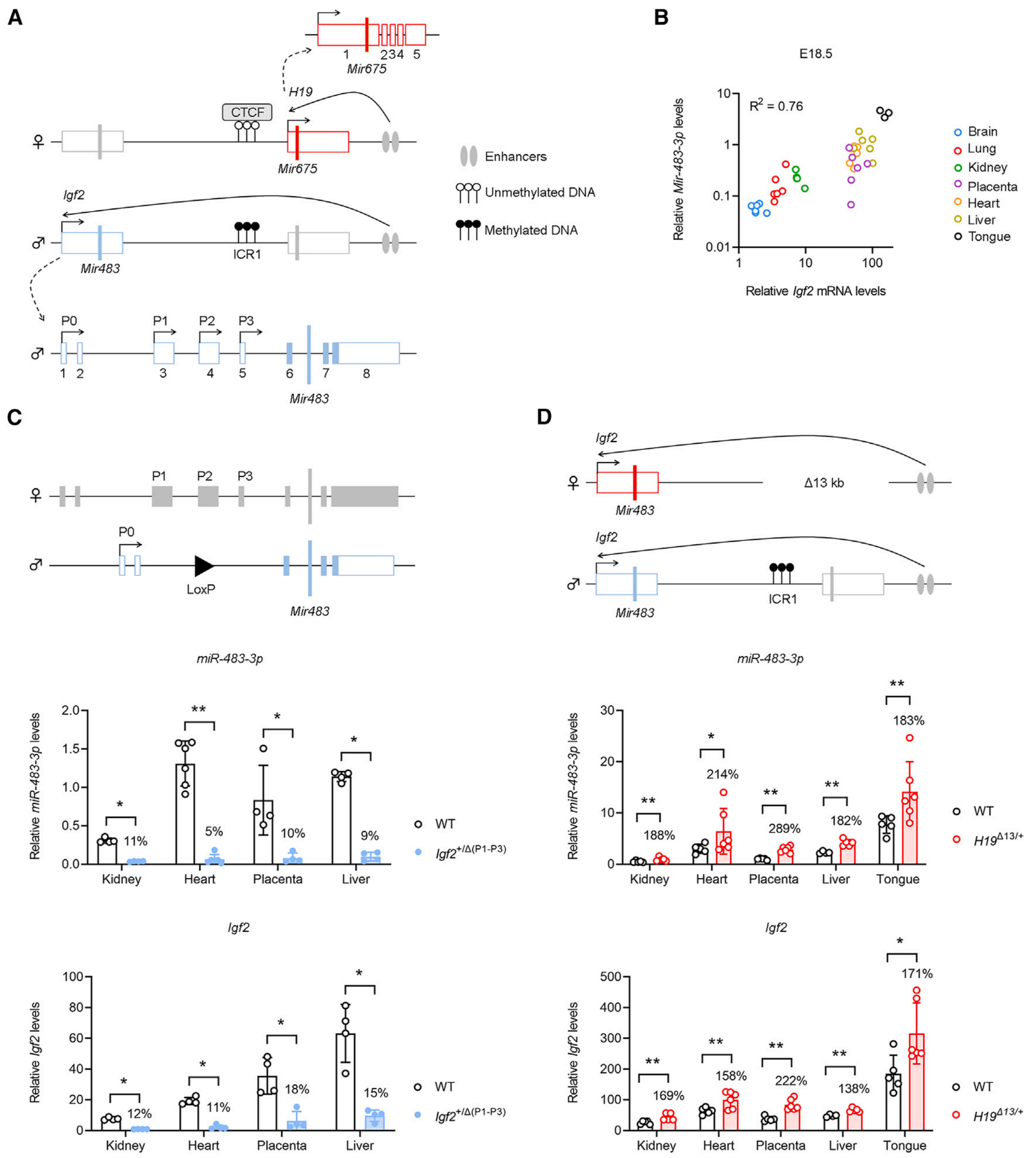

**Figure 1. Developmental regulation of the mouse *Mir483* expression**

(A) Location of *Mir483* and *Mir675* within the *Igf2*/*H19* imprinted domain and regulation by the methylation-sensitive CCCTC-binding factor (CTCF) boundary at ICR1. P0–P3, alternative *Igf2* promoters; numbers indicate exons.

(B) Expression of *miR-483-3p* shows a positive correlation with *Igf2* expression in fetal organs ($p < 0.001$; $n = 38$ pairs with $n = 3$–6 replicates per organ).

(C) Deletion of fetal *Igf2* promoters P1–P3 from the paternal allele ($Igf2^{+/\Delta(P1-P3)}$ model, top) abolishes *miR-483-3p* expression (middle) and *Igf2* expression (bottom) in multiple fetal organs at E18.5 ($n = 4$–6 samples per group).

(D) *Igf2* loss of imprinting (achieved by deletion of ICR1 and *H19* on the maternal allele—$H19^{\Delta13/+}$ model—top) results in increased relative levels of *miR-483-3p* expression (middle) and *Igf2* expression (bottom) in multiple fetal organs at E18.5 ($n = 4$–6 samples per group). Levels of *miR-483-3p* were normalized against *Snord70/snoRNA234* and *Igf2* against the geometrical mean of *Ppia*, *Pmm1*, and *Hprt*.

Genomic features shown in (A), (C), and (D) are not drawn to scale and are for representation purposes only. For (C) and (D), data are individual values with averages ± standard deviation (SD); percentage values above the mutant columns indicate ratios of mutant/wild type (WT); $n = 4$–6 samples per group; *$p < 0.05$ and **$p < 0.01$ by Mann-Whitney tests followed by two-stage step-up (Benjamini, Krieger, and Yekutieli) FDR < 5%. See also Figures S1, S2, and S3.

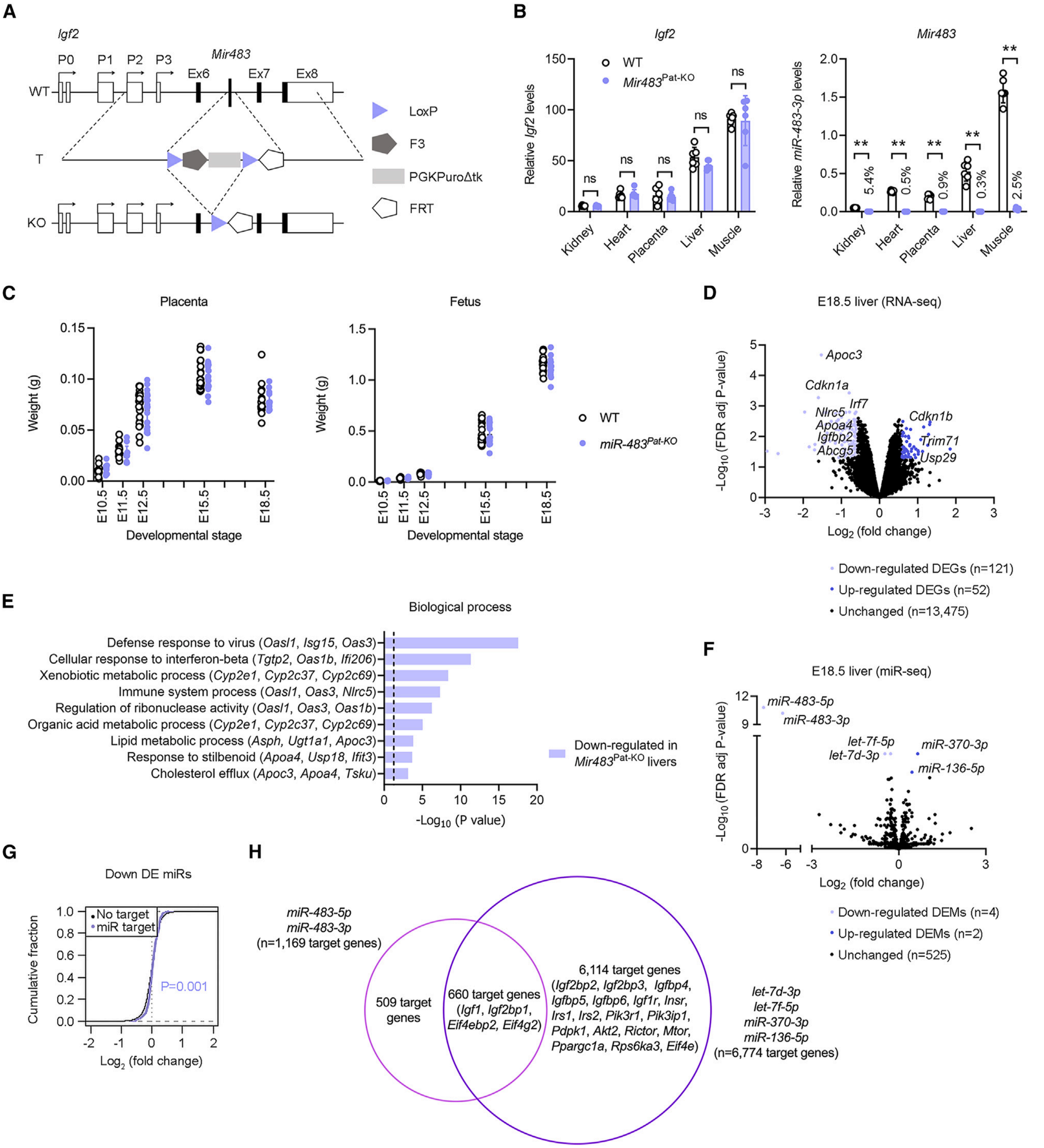


**Figure 2. Developmental phenotyping of the *Mir483* knockout**

(A) Schematic representation of the *Igf2* wild-type allele (WT), targeting vector (T), and knockout allele (KO) obtained upon *Cre*-mediated deletion of the selection cassette. Diagram representation is not at scale. See also Figure S4.

(B) RT-qPCR levels of *Igf2* and *miR-483-3p* in E18.5 fetal organs upon paternal transmission of the deletion (*Mir483*$^{Pat-KO}$) and WT littermate controls ($n$ = 6 samples per group). Levels of *Igf2* were normalized against the geometrical mean of *Ppia*, *Pmm1*, and *Hprt* and *miR-483-3p* levels against the geometrical mean of *Snord70/snoRNA234* and *Snord68/snoRNA202*.

(C) Fetal and placental growth kinetics (n = 4–6 litters at each developmental stage).

(D) Volcano plot representation of differentially expressed genes (DEGs) identified by RNA-seq in E18.5 livers (*Mir483*$^{Pat-KO}$ versus WT). Downregulated and upregulated DEGs (FDR < 0.05, fold change >1.5) are shown with light and dark purple dots, respectively. See also Table S1.

(legend continued on next page)

Mendelian distribution ($n = 21$ WT and $n = 16$ *Mir483*$^{Pat\text{-}KO}$ in four mixed litters at E18.5, Fisher's exact test, $p = 0.65$) or fetal and placental growth kinetics (Figures 2C and S5C).

To characterize, at the molecular level, the potential impact of constitutive *Mir483* loss, we performed unbiased transcriptome analyses in E18.5 livers of *Mir483*$^{Pat\text{-}KO}$ and WT littermates, using both RNA sequencing (RNA-seq) and miR sequencing (miR-seq). Liver was chosen because it is the major producing organ of IGF1 and IGF2. RNA-seq analysis identified 173 differentially expressed genes (DEGs; 121 and 52 downregulated and upregulated in *Mir483*$^{Pat\text{-}KO}$, respectively) (Figure 2D and Table S1). Consistent with the normal growth observed in the *Mir483*$^{Pat\text{-}KO}$ mutants, expression of *Igf2* and *Igf1* was unaltered (Table S1). Expression of IGF1 and IGF2 was also normal at the protein level in E18.5 livers (Figures S5D and S5E). However, DAVID analysis identified several biological processes enriched in DEGs downregulated in *Mir483*$^{Pat\text{-}KO}$ livers, related to lipid metabolism and immune processes (Figure 2E). miR-seq analysis confirmed the expected lack of *miR-483-3p* and *miR-483-5p*. In addition, there were four other differentially expressed miRs (DEMs; *miR-370-3p* and *miR-136-5p* upregulated and *let-7f-5p* and *let-7d-3p* downregulated) in the E18.5 liver (Figure 2F and Table S2). To assess the contribution of DEMs to mRNA levels of predicted targets, a cumulative fraction analysis was employed (see STAR Methods). Interestingly, this analysis revealed a propensity for increased expression of mRNAs with conserved sites for the four downregulated DEMs when considered collectively (Figure 2G). However, no significant effect on mRNA targets of *miR-483-3p* and *miR-483-5p* was observed, when analyzed individually, or on the targets of the two upregulated DEMs analyzed together (Figure S5F). Overall, this analysis suggests the existence of a regulatory buffering network involving miRs, which may contribute to minimizing the impact of *Mir483* deletion. In further support of this hypothesis, we note that the predicted gene targets of the four DEMs include family members of the INS-IGF pathway and downstream signaling genes (Figure 2H).

Post-natal growth of *Mir483*$^{PAT\text{-}KO}$ mutants was indistinguishable from that of WT littermates (Figures S6A–S6C), as were the levels of IGF1 in plasma and liver at post-natal day 21 (Figure S6D). In addition, fat mass, lean mass, bone mass density (Figure S6E), and glucose tolerance (Figure S6F) were similar between young adult *Mir483*$^{PAT\text{-}KO}$ mutants and WT littermates.

## Overexpressing *Mir483* causes fetal growth restriction and midgestation lethality

To fully establish the function of *Mir483*, gain-of-function *in vivo* models were generated. In our initial approach, using homologous recombination in ES cells, we inserted one copy, three tandem extra copies, and five tandem extra copies of *Mir483* at the endogenous *Igf2* locus (Figures 3A and S7A–S7D), but achieved germline transmission of only the five-tandem-copy transgene (referred to as *Mir483*$^{5C}$). Chimeric males transmitted the transgene to offspring, but the elevated levels of *Mir483*$^{5C}$ (observed for both *miR-483-3p* and *miR-483-5p*, see Figure 3B) caused all the embryos to arrest in development, with complete reabsorptions by E13.5. At E11.5, fetuses, but not placentae, showed severe growth restriction (~58% of normal) (Figure 3C) despite similar levels of *miR-483* overexpression in both the embryo and the placenta (Figure S7E). Transcriptome analysis of E10.5 *Mir483*$^{5C}$ embryos by RNA-seq identified 85 DEGs (80 and 5 downregulated and upregulated in *Mir483*$^{5C}$ embryos, respectively) (Figure 3D and Table S3). DAVID analysis uncovered several biological processes enriched in DEGs downregulated in *Mir483*$^{5C}$ mutants, related to lipid metabolism, blood coagulation, and transport (Figure 3E). Importantly, *Igf2* was one of the downregulated DEGs in *Mir483*$^{5C}$ embryos (Figure 3D). DEGs upregulated in *Mir483*$^{5C}$ mutants included two granzymes (*Gzmd* and *Gzmg*) but did not highlight any specific biological process (Figure 3D).

Mass-spectrometry-based protein quantification of ~4,000 proteins revealed a small number of differentially expressed proteins (DEPs; fold change >1.5, with false discovery rate [FDR] < 0.05) in whole E10.5 *Mir483*$^{5C}$ embryos (Figure 3F). Upregulated DEPs were related to induction of cytolysis (e.g., granzymes), and downregulated DEPs were implicated in epigenetic processes (e.g., histones) (Figure 3G and Table S4). The list of DEPs downregulated in *Mir483*$^{5C}$ mutants also included IGF2 (Figure 3G and Table S4). Downregulation of *Igf2*/IGF2 in *Mir483*$^{5C}$ embryos, but not placentae, was validated by RT-qPCR and western blots in independent samples at E11.5 (Figures 3H and 3I). Notably, the greatest mRNA reduction was observed for the *Igf2*-P2 transcript (Figure S7F), which contains, as mentioned before, a 7–8mer seed sequence in the 5′ UTR exon 4 (Figure S1E).

In a second approach, we engineered an ectopic inducible Tet-off transgene (referred to as *iTg*$^{Mir483}$) (Figures 4A and S8A–S8D), the developmental expression of which can be inhibited by the administration of doxycycline (Dox) during pregnancy and beyond (Figure 4B). Similar to the *Mir483*$^{5C}$ endogenous transgenic model, we found that high levels of *miR-483* expression during intrauterine development (Figure 4B) caused fetal, but not placental, growth restriction (Figure 4C) and lethality (Figure S8E). The livers of *iTg*$^{Mir483}$ mutants were disproportionally smaller than in controls (Figure 4C). The growth

(E) Top-scoring biological processes enriched in DEGs by DAVID analysis. Three DEGs with highest fold changes are listed in parentheses. The dotted line corresponds to an FDR-corrected $p$ value of 0.05. See also Table S1.

(F) Volcano plot of differentially expressed microRNAs (DEMs) identified by miR-seq in E18.5 livers (*Mir483*$^{Pat\text{-}KO}$ versus WT). Downregulated and upregulated DEMs are shown with light and dark purple dots, respectively. See also Table S2.

(G) Cumulative fractions of mRNA fold changes between E18.5 livers of *Mir483*$^{Pat\text{-}KO}$ mutants and WT littermates for putative targets of the four downregulated DEMs shown in (F). Statistical differences between distributions were calculated using two-sided Kolmogorov-Smirnov tests.

(H) Venn diagram depicting the overlap between predicted target genes of *miR-483-5p* and *miR-483-3p* and the four DEMs identified by miR-seq in E18.5 livers of *Mir483*$^{Pat\text{-}KO}$ versus WT (the same predicted target genes by multiple miRs were counted only once). Highlighted genes are involved in the INS-IGF signaling pathways and are targets of at least one of the indicated miRs.

Data are individual values, with averages ± SD in (B) and (C), and percentage values indicate ratios of *Mir483*$^{Pat\text{-}KO}$/WT; ns, non-significant; $^{**}p < 0.01$ by multiple Mann-Whitney tests with FDR (<5%) correction in (B). See also Figures S4, S5, and S6.

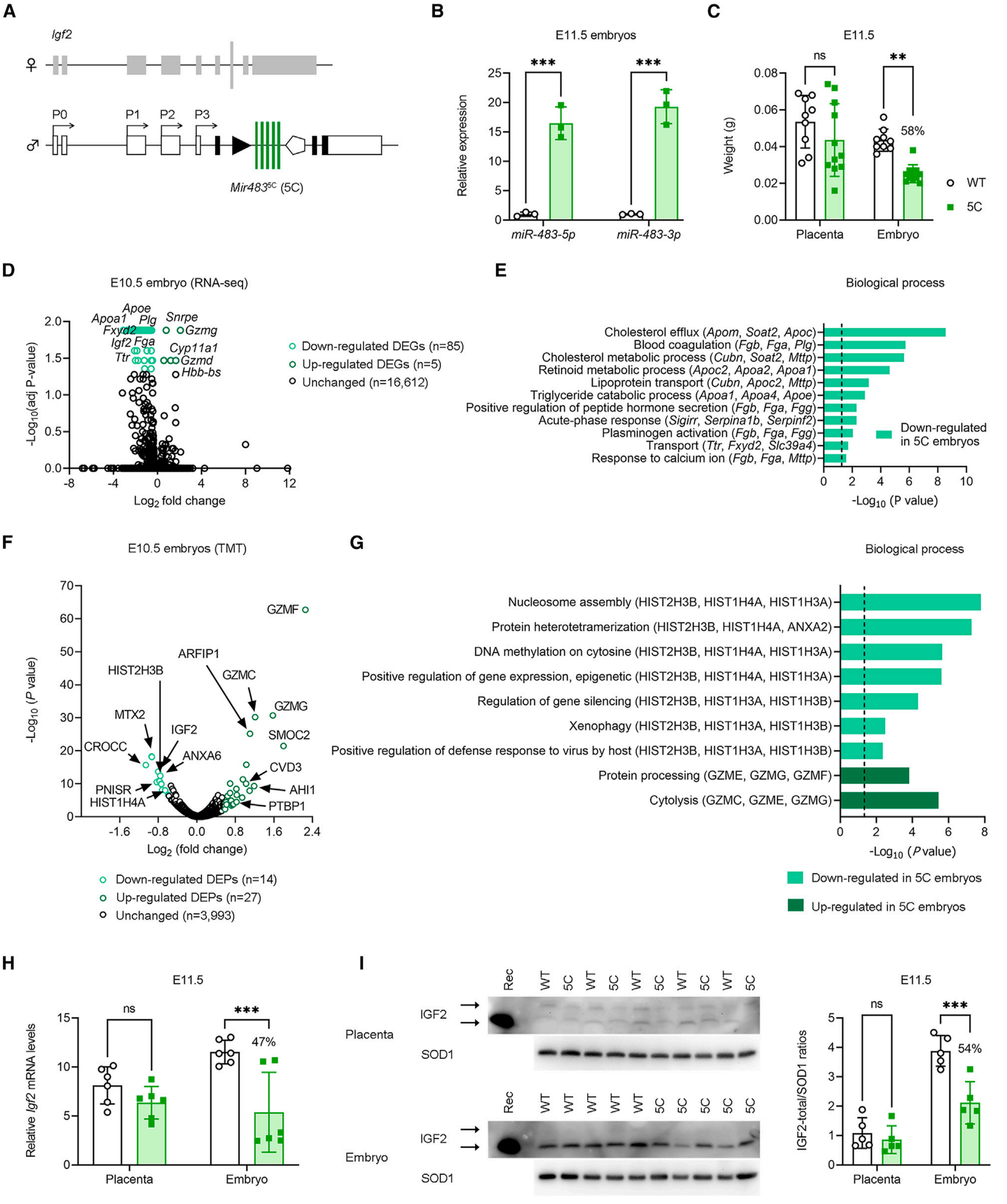


**Figure 3. Overexpression of *Mir483* (*Mir483*$^{5C}$–5C) from the endogenous *Igf2*/*Mir483* locus**

(A) Gene targeting of the five-copy (5C) tandem array inserted at the endogenous locus. Genomic features are not drawn to scale and are for representation purposes only. See also Figure S7.

*(legend continued on next page)*

restriction of E13.5 fetuses was associated with reduction of IGF2 and IGF1 proteins (Figure 4D). Fetal lethality in $iTg^{Mir483}$ was of later onset compared to $Mir483^{5C}$ mutants, i.e., from E15.5, and was caused by a range of severe defects, in particular, malformations of the heart and the great intrathoracic arteries (Figure 4E; see Table S5 for the list of developmental abnormalities). Importantly, the lethality and growth phenotypes were rescued by exposure to doxycycline during pregnancy (Figures 4F, S8E, and S8F).

## Growth and metabolic defects in mice with continuous *Mir483* overexpression after birth

In humans, in contrast to mice, both *IGF2* and *MIR483* continue to be expressed in the post-weaning period and throughout adulthood. We exploited the temporal inducible versatility of $iTg^{Mir483}$ by extending the post-natal expression of *Mir483* independent of *Igf2* continuously from birth to adulthood, up to 15 weeks of age. We then assessed the consequences for growth, body composition, and glucose homeostasis (Figure 5A). Both male and female $iTg^{Mir483}$ mice displayed post-weaning growth restriction (males 70% N, females 86% N at 15 weeks of age, Figure 5B), which resulted from the overexpression of *Mir483* (Figures 5C and S9A). The growth-restricted $iTg^{Mir483}$ female and male mice had a lower lean mass (Figure 5D), but showed increased adiposity (Figure 5D), compared to controls (Figure 5E). Fat mass gain in gonadal and subcutaneous depots was particularly extensive at 8 weeks (ranging from 250% to 477% of control fat depot weights) compared to 15 weeks (Figures 5F and S9B), suggesting limited capacity for further adipocyte expansion at the later stage. The increased adiposity was not related to food intake, which was not different in $iTg^{Mir483}$ males but, in fact, significantly reduced in $iTg^{Mir483}$ females (Figure S9C). Adipocytes of $iTg^{Mir483}$ were larger compared to controls, as shown *in situ* for the gonadal fat in males (Figure 5G), and *ex vivo* in both sexes (Figure 5H), with a notable reduction in the percentage of smaller cells (Figures 5G and S9D) and the total number of adipocytes per fat pad (Figure 5G). The increased percentage of larger adipocytes in the gonadal fat was accompanied by downregulation of *Ttc36* and upregulation of *Arhgdig* (Figure 5I), genes recently identified as markers of visceral fat adipocyte hypertrophy in human.[32] However, the increased adipocyte size could not be explained by an intrinsic increase in expression of lipid transporters (Figure S9E), a decreased expression of lipases (which mediate lipolysis; Figure S9F), or an increased expression of enzymes implicated in triglyceride synthesis (Figure S9G). In addition, protein levels of GDF3, a target of *miR-483-3p*,[21] were similar in mature adipocytes of $iTg^{Mir483}$ and control mice (D.S.F.-T., unpublished data). Furthermore, expression of leptin, known to inhibit the expression of adipogenic genes in the white adipose tissue,[33] was upregulated in adipocytes of $iTg^{Mir483}$ mice (Figure 5J).

We next profiled circulating lipids and observed evidence for a modest dyslipidemia in both sexes (Figure 6A). Importantly, we detected drastic reductions in IGF1 levels in both the circulation and the organs of $iTg^{Mir483}$ mice (Figures 6B, S10A, and S10B) and increased circulating growth hormone (GH) levels (~7-fold) (Figure 6C). Given that the increased lipid accumulation in the fat depots and serum dyslipidemia did not relate to major molecular changes in the adipocytes, we analyzed the morphology and function of the liver as a key organ for lipid production. The $iTg^{Mir483}$ livers were smaller compared to controls (Figure 6D), but extensively vacuolated (Figure 6E), suggestive of liver steatosis. Transcriptome analysis by RNA-seq in livers of 15-week-old $iTg^{Mir483}$ and control mice identified 1,373 DEGs (324 and 1,049 downregulated and upregulated in $iTg^{Mir483}$ livers, respectively) (Figure 6F and Table S6). Consistent with the morphological changes identified at the histological level, DAVID analysis confirmed a molecular signature of altered lipid metabolism. Accordingly, biological processes enriched in downregulated DEGs highlighted a negative regulation of lipid storage, while biological processes enriched in upregulated DEGs were dominated by genes implicated in cholesterol biosynthesis, lipid transport, and other lipid metabolic processes, in addition to inflammatory response, extracellular matrix organization, pyroptosis, and angiogenesis (Figure 6G and Table S6). The transcriptional upregulation of genes implicated in the production of major types of lipids, such as fatty acids, cholesterol, and triglycerides, was further confirmed by RT-qPCR, with evidence for sex-dependent effects for some specific genes (Figures S10C–S10E). In addition, we found upregulation of several genes implicated in lipoprotein turnover, as well as key transcriptional regulators of hepatic liver metabolism (Figure S10F). Therefore,

(B) Relative expression of *miR-483-5p* and *miR-483-3p* measured by RT-qPCR in whole-embryo lysates at E11.5 ($n$ = 3 samples/group). Levels of *miR-483-5p* and *miR-483-3p* were normalized against *Snord68/miRNA234* and are presented relative to the wild-type (WT) levels, arbitrarily set to 1.

(C) Placenta and embryo weights at E11.5 ($n$ = 9–11/group).

(D) Volcano plot of DEGs identified by RNA-seq in E10.5 embryos ($Mir483^{5C}$ versus WT). Downregulated and upregulated DEGs (FDR < 0.05, fold change >1.5) are shown with light and dark green circles, respectively. See also Table S3.

(E) Top-scoring biological processes enriched in DEGs by DAVID analysis. Three DEGs with highest fold changes are listed in parentheses. See also Table S3.

(F) Volcano plot representation of differentially expressed proteins (DEPs) identified by TMT (tandem mass tag) proteomics in surviving embryos at E10.5 ($n$ = 3 per genotype). Proteins downregulated or upregulated (fold change >1.5, Benjamini-corrected $p < 0.00144$) are presented in light green and dark green, respectively. See also Table S4.

(G) Top-scoring biological processes enriched in DEPs by DAVID analysis. See also Table S4.

(H) *Igf2* is downregulated in 5C embryos, but not placentas at E11.5 by RT-qPCR ($n$ = 6 samples per group). Levels of *Igf2* were normalized against the geometrical mean of *Gapdh*, Sdha, and *Pmm1*.

(I) Left: IGF2 protein levels by western blot in whole placenta and embryo lysates at E11.5 ($n$ = 5 per group. Rec, recombinant mouse IGF2 protein; upper and lower arrows indicate the 18 kDa pro-IGF2 and 7.4 kDa mature IGF2, respectively; SOD1 [19 kDa], internal control for loading). Right: quantification of IGF2-total/SOD1 ratios by western blot ($n$ = 5 samples/group) shown relative to WT placenta, arbitrarily set to 1.

The dotted line in (E) and (G) corresponds to FDR-corrected $p$ value of 0.05. Data are individual values, with averages ± SD in (B), (C), (H), and (I) and percentages indicate ratios of 5C/WT; ns, non-significant; **$p < 0.01$ and ***$p < 0.001$ by two-way ANOVA followed by Šídák's multiple comparisons tests in (B), (C), (H), and (I). See also Figure S7.

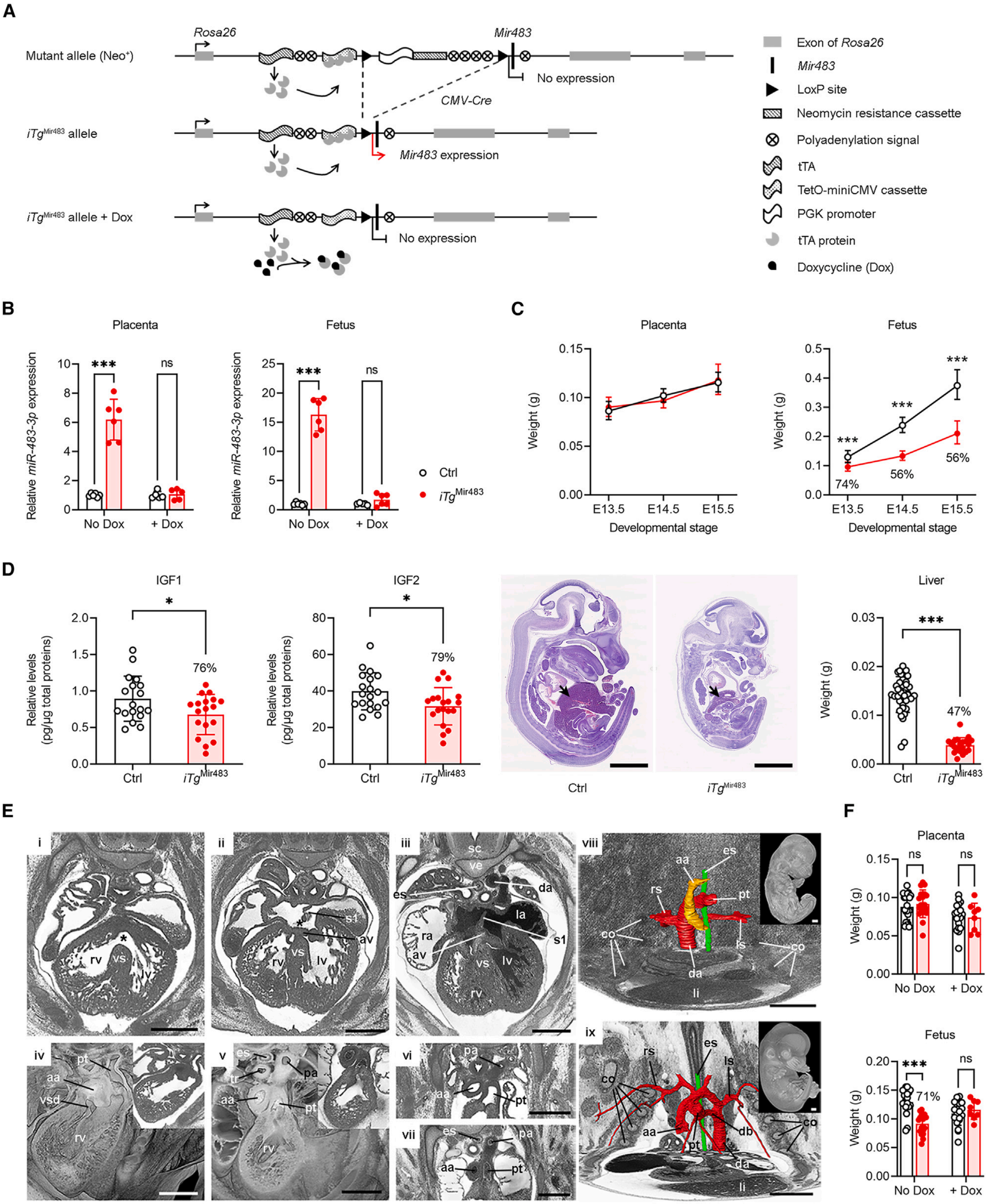


**Figure 4. Pre-natal developmental phenotyping of a Tet-off inducible overexpressor *Mir483* transgenic model (*iTg*[Mir483])**

(A) Targeting of an inducible *Mir483* transgene by homologous recombination at the *Rosa26* locus. Expression of the *Mir483* transgene can be controlled by the administration of doxycycline (Dox). Genomic features are not drawn to scale and are for representation purposes only. See also Figure S8.

(legend continued on next page)

our findings suggest that the main site for lipid overproduction in the *iTg*$^{Mir483}$ mutants was the liver.

Since the *iTg*$^{Mir483}$ model shows pronounced overexpression of *Mir483*, we were interested to assess the specificity of the effects observed at the transcriptome level caused by miR overexpression. Our analyses of distribution of gene expression changes demonstrated repression of mRNAs with conserved putative targets of *miR-483-3p*, but not *miR-483-5p*, when analyzed individually (Figure 6H). These findings suggest that the phenotypic changes observed in *iTg*$^{Mir483}$ livers are mediated, at least in part, by direct effects of *Mir483* on its target genes. In addition, we found 32 DEGs that were common in livers of E18.5 *Mir483*$^{Pat\text{-}KO}$ and adult *iTg*$^{Mir483}$ mutants (Figure 6I and Table S6).

The *iTg*$^{Mir483}$ mice showed pronounced multi-organ dysmorphic growth, with disproportional reduction of skeletal muscle and pancreas mass, proportionally smaller brain, and splenomegaly (Figure S11A). Muscle fiber cell areas were smaller in the *vastus lateralis* of *iTg*$^{Mir483}$ male mice (Figure S11B). Despite these changes, glucose homeostasis, assessed by oral glucose tolerance tests (OGTTs), in young mice (13 weeks) of both sexes remained normal (Figure S11C). Although mildly hyperinsulinemic upon fasting (Figure S11E), insulin content per gram pancreatic tissue was reduced in both sexes (Figure S11D). This was consistent with an impairment in glucose-induced insulin-secretion (GSIS), assessed during OGTT, particularly in females (Figure S11E). These data suggest that *iTg*$^{Mir483}$ mice are insulin sensitive, despite the increased adiposity, findings that are consistent with the observed normal OGTT profiles. Further evidence in support of an improved insulin sensitivity in *iTg*$^{Mir483}$ mice was provided by the finding of a genotype-dependent increase in pAKT levels in the adipose tissue (Figure S11F).

### Growth defects in *iTg*$^{Mir483}$ mice are rescued by systemic IGF1 infusion

Treating male *iTg*$^{Mir483}$ mice with a constant infusion of human IGF1 for 4 weeks (Figure 7A) rescued whole body growth restriction by the end of treatment (Figure 7B). However, IGF1 treatment of *iTg*$^{Mir483}$ did not rescue the increased adiposity phenotype (Figure S12A); instead, the gonadal fat pads increased even further in weight (Figure S12B). The brain, liver, and *vastus lateralis* also increased in size upon treatment (Figure S12C), with a trend for improved total lean mass (Figure S12A). Further evidence for an *in vivo* link between *miR-483* and IGF1 regulation in the mouse is observed in *Igf2*$^{Pat\text{-}KO}$ and *Igf2*$^{+/LacZ}$ knockouts.[34,35] In both of these models, which lack both *Igf2* and *Mir483* (Figures 7C and S12C), *Igf1* mRNA and IGF1 protein levels were increased in the fetal liver.

To establish a direct link between *miR-483* and *Igf1*, as a predicted target gene in the mouse (Figure 7D), we performed Ago2 immunoprecipitation (IP) experiments in undifferentiated 3T3-L1 cells that showed immunoprecipitation of IGF1 with the miR processing machinery (Figure 7E). We then cloned the mouse 3′ UTR of *Igf1* with either mutated *miR-483* binding sites or wild-type (i.e., non-mutated binding site) controls into luciferase reporter vectors and transfected those into low-expressing *MIR483* human HEK-293 cells, in the presence of increasing concentrations of *miR-483* mimic. This led to a decrease in luciferase expression in wild-type 3′ UTR-transfected cells, which was not observed in 3′ UTR-mutated-transfected cells (Figure 7F). Conversely, *miR-483* antagonist luciferase-reporter-based experiments conducted in human HepG2 *MIR483*-expressing cells led to a dose-dependent release of *miR-483* silencing (Figure 7G).

## DISCUSSION

The IGF signaling system is a major regulator of growth in all vertebrate species.[36,37] *IGF2*, but not *IGF1*, is regulated by genomic imprinting in placental mammals.[38] Mouse *Igf1* and *Igf2* genes contain conserved target sequences for potential *Mir483* regulation,[17–19,39–41] which are located at the 3′ UTR of *Igf1* and 3′ and 5′ UTRs (P2 isoform) of *Igf2*. Here, we provide strong evidence that *Mir483* regulates levels of IGF1 and IGF2 *in vivo* through the analysis of two transgenic mouse models of overexpression: *Mir483*$^{5C}$, which contains five *Mir483* copies inserted at the endogenous locus, and *iTg*$^{Mir483}$, a one-copy Tet-off inducible *Mir483* transgene inserted at the ubiquitous

(B) Relative levels of *miR-483-3p* expression (normalized against the geometrical mean of *Snord70/snoRNA234* and *Snord68/snoRNA202*) in whole placentae and fetuses at E13.5 without or with Dox exposure (1 mg/mL in the drinking water; *n* = 5–6 samples/group).

(C) Without Dox administration, placental growth is normal, while fetal growth is compromised, including that of liver, shown at E14.5. The images show representative midsagittal sections of E14.5 fetuses (Ctrl, control; arrows point to the liver; scale bars, 2.5 mm).

(D) IGF1 and IGF2 protein levels by ELISA in E13.5 fetuses and normalized against total protein content measured by a BCA protein assay (*n* = 18–19 samples/group).

(E) Representative high-resolution episcopic microscopy (HREM) scans (*n* = 4 wild-type [WT] and *n* = 6 *iTg*$^{miR483}$ E14.5 fetuses) identify severe malformations of the heart and the great intrathoracic arteries, leading to lethality from E15.5 onward. (i–iii) Axial HREM sections showing mutants with septum defects: ventricular septal defect (i, asterisk), atrial septal defect (ii, asterisk), and a WT control (iii); (iv and v) axially sectioned volume-rendered models showing a mutant with a double outlet right ventricle (iv) and a WT control (v); (vi and vii) the central segment of axial HREM sections showing a mutant with coarctation of the aorta (vi) and a WT control (vii); (viii and ix) surface models of intrathoracic arteries and esophagus, illustrating a complex malformation of the great intrathoracic arteries, which combines a right-sided aortic arch, a type B aortic arch interruption, and a left sided retroesophageal subclavian artery (viii) and a WT control (ix). The insets show entire embryos viewed from the right (note the signs of autolysis in viii). Scale bars, 500 μm; aa, ascending aorta; av, atrioventricular cushion; co, costa; da, descending aorta; db, ductus Botalli; es, esophagus; la, left atrium; li, liver; ls, left subclavian artery; lv, left ventricle; pa, preductal aorta; pt, pulmonary trunk; ra, right atrium; rs, right subclavian artery; rv, right ventricle; sc, spinal cord; s1, septum primum; tr, trachea; ve, vertebra; vs, ventricle septum; vsd, ventricle septum defect. See also Table S5.

(F) Placental and fetal weights are normal at E13.5 upon Dox administration in the drinking water (1 mg/mL) from the beginning of pregnancy (n = 8–20 per group). Data are individual values, with averages ± SD in (B), (C, bottom), (D), and (F) or averages ± 95% CI in (C, top) and percentage values indicate ratios of *iTg*$^{Mir483}$/Ctrl; ns, non-significant; *$p < 0.05$ and ***$p < 0.001$ by two-way ANOVA followed by Šídák's multiple comparisons tests in (B) and (F), a mixed-effects model in (C, top), a Mann-Whitney test in (C, bottom), and unpaired *t* tests with Welch's correction in (D). See also Figure S8.

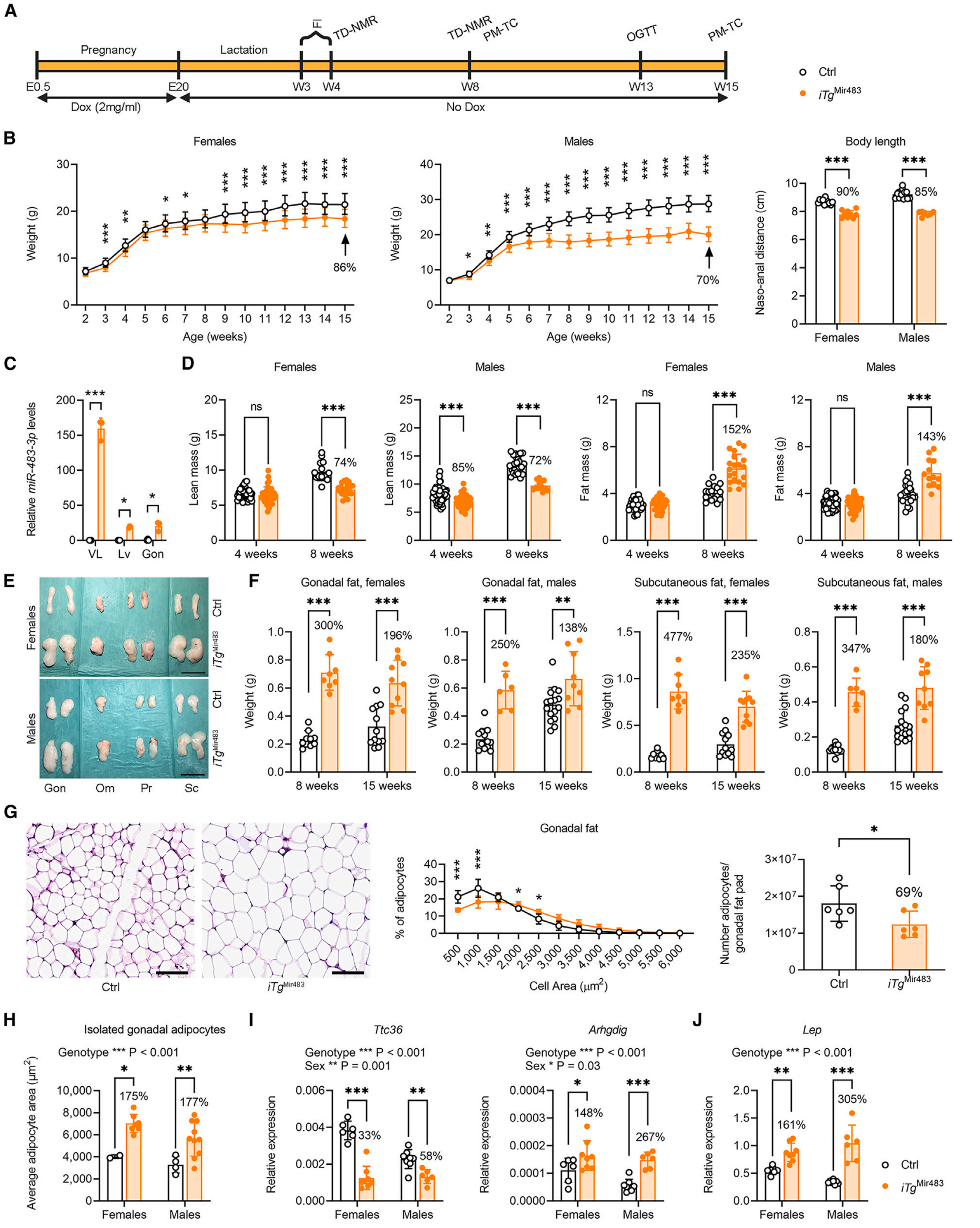


*(legend on next page)*

*Rosa26* locus. Inducible high levels of overexpression in *iTg*$^{Mir483}$ mutants during intrauterine life led to reductions in the expression of IGF1 (by ~24% at E13.5) and IGF2 (by 21% at E13.5) and severe fetal growth restriction by E15.5 (56% N). Both *Mir483*$^{5C}$ and *iTg*$^{Mir483}$ mutants led to fetal, but not placental, growth restriction, and midgestation lethality. The lack of a placental growth phenotype is consistent with normal levels of both IGF1 and IGF2 in placenta, despite the degree of overexpression of *Mir483* being similar in the placenta and fetus. Possible explanations for the normal IGF levels and lack of a growth phenotype are: (1) *Igf1* is expressed at low levels in the mouse placenta, and consistently IGF1-deficient mice lack a placental growth phenotype,[42,43] and (2) the major placenta-specific *Igf2* transcript P0 lacks target sites for *Mir483* regulation, and the 5′ UTR isoform (P2), which is likely to be regulated by *Mir483*, is expressed at lower levels in the placenta compared to the fetus.

Midgestation lethality timing differs between *Mir483*$^{5C}$ and *iTg*$^{Mir483}$ (E13.5 versus E15.5, respectively). Phenotypic analyses of the E14.5 *iTg*$^{Mir483}$ embryos revealed that cardiovascular defects (100% prevalence) are likely the cause of the lethality. The *Mir483* target genes leading to cardiovascular dysfunction remain to be elucidated. The fetal lethality in *iTg*$^{Mir483}$ mice can be rescued by switching off the ectopic *Mir483* expression through the administration of doxycycline during pregnancy, thus showing that it is the overexpression of *Mir483* that causes the lethality phenotype. The *iTg*$^{Mir483}$ system also offers the possibility of overexpressing *Mir483* specifically in the post-natal period by removing doxycycline and causing transgenic activity.

In the current study, we aimed to explore the effects of uncoupled expression of *Mir483* from *Igf2* in the post-natal period, using *iTg*$^{Mir483}$. This model of post-natal continued expression is relevant to human physiology, since *IGF2* and *MIR483* are expressed throughout adulthood in a wide variety of tissues, although levels are thought to be higher pre-natally. By contrast, in wild-type mice, *Igf2* and its cotranscribed *Mir483* are largely silenced after weaning (except for skeletal muscle, brain, and adult stem cells). Activation of *Mir483* independent of *Igf2*, from birth to adulthood, caused post-natal growth stunting, starting before weaning, thus demonstrating a likely role in pre-weaning growth. This growth phenotype was more pronounced in males compared to females. An adiposity and fatty liver phenotype was associated with the growth impairment of key metabolic organs, suggesting that *miR-483* is a metabolic regulator through yet to be identified target genes, in addition to *Igf1*, which was severely downregulated (~50%–90%) in most organs and in the circulation. Presumably, as a result of the low levels of IGF1, there was a sharp increase in circulating GH levels, consistent with the well-established negative feedback loop between liver IGF1 and pituitary GH (GH is not a direct target for *Mir483* regulation). Based on experiments performed on *Igf1*-deficient mouse models, a phenotype of insulin resistance might be expected[44] in the *iTg*$^{Mir483}$ overexpressor. However, despite the increased adiposity, there was no evidence of insulin resistance—these mice also did not develop glucose intolerance and had only low-level dyslipidemia. The absence of hyperinsulinemia and the finding of increased pAKT levels in the adipose tissue suggest that these mice have improved adipose tissue insulin sensitivity. The contribution of IGF1 deficiency to some of the *Mir483* phenotypes is clear in terms of whole body and local organ growth, including the effect on lean mass, as IGF1 is a known key regulator of muscle mass.[45,46] Infusion of systemic IGF1 in the growth-impaired *iTg*$^{Mir483}$ restored normal growth patterns, thus providing evidence for a causal effect of IGF1 on the growth phenotype. Moreover, reduced levels of IGF1 in *iTg*$^{Mir483}$ may explain the reduced number of mature adipocytes per fat depot, given the role of IGF1 in stimulating pre-adipocyte differentiation.[47,48] How *Mir483* overexpression leads to

**Figure 5. Post-natal *Mir483* overexpression leads to growth retardation, altered body composition, and increased adiposity**

(A) Timeline of the experimental setup: Dox (2 mg/mL) was administered in the drinking water throughout pregnancy, after which Dox was withdrawn, allowing expression of *Mir483* from the *iTG*$^{Mir483}$ transgene. Food intake (FI) was measured over a period of 1 week (weeks 3–4, W3–W4). TD-NMR (time-domain nuclear magnetic resonance) was performed at W4 and W8, OGTT (oral glucose tolerance tests) at W13, and post-mortem tissue collection (PM-TC) at W8 and W15.
(B) *iTG*$^{Mir483}$ mice show post-natal growth restriction in both females ($n$ = 10–12) and males ($n$ = 9–16) and reduced total body length at W15 ($n$ = 7–14 mice per sex and genotype).
(C) Relative levels of *miR-483-3p* measured by RT-qPCR in W15 organs (VL, *vastus lateralis*; Lv, liver; and Gon, gonadal fat) from *iTG*$^{Mir483}$ mutants and littermate controls (Ctrl). Levels of *miR-483-3p* were normalized against the geometrical mean of *Snord70/snoRNA234* and *Snord68/snoRNA202* ($n$ = 2–3 samples/group).
(D) TD-NMR analysis shows reduced lean mass and excessive fat accumulation in *iTG*$^{Mir483}$ mutants compared to littermate controls at W4 and W8 ($n$ = 13–51 mice per group).
(E) Individual fat pads are larger in W8 *iTG*$^{Mir483}$ mutants compared to controls: Gon, gonadal fat; Om, omental fat; Pr, peri-renal fat; and Sc, subinguinal subcutaneous fat (scale bars, 2 cm).
(F) Gonadal fat pads and subinguinal subcutaneous fat pads are significantly heavier in W8 and W15 *iTG*$^{Mir483}$ adults compared to age-matched controls ($n$ = 6–16 per group).
(G) Adipocytes are larger in the gonadal fat pads of W15 *iTg*$^{Mir483}$ mutant males compared to age-matched controls (left, representative H&E-stained sections, and middle, distribution of adipocyte cell area), with an estimated lower number of mature adipocytes/fat pad (right; $n$ = 6 samples per group; scale bars, 100 μm).
(H) Average cell area from gonadal fat of W8 *iTg*$^{Mir483}$ mutants compared to age-matched controls ($n$ = 2–10 samples per group).
(I) Expression patterns of genes that correlate with visceral adipocyte area (i.e., reduced *Ttc36* and increased *Arhgdig* mRNA levels) support the adipocyte hypertrophy observed in the gonadal fat of W8 *iTg*$^{Mir483}$ mutants compared to age-matched controls ($n$ = 6–8 per group).
(J) Increased expression of *Lep* gene, encoding leptin in the gonadal fat of W8 *iTg*$^{Mir483}$ mutants compared to age-matched controls ($n$ = 6–8 per group).
Data are presented as averages ± 95% CI in (B) (first two graphs on the left); individual values with averages ± SD in (B) (graph on the right), (C), (D), (F), (G) (graph on the right), (H), (I), and (J); or averages ± SD in (G) (middle), and values indicate ratios of *iTG*$^{Mir483}$/Ctrl and percentage values indicate ratios of *iTG*$^{Mir483}$/Ctrl. ns, non-significant; *$p < 0.05$, **$p < 0.01$, and ***$p < 0.001$ by a mixed-effects model in (B) (first two graphs on the left); two-way ANOVA followed by Šídák's multiple comparisons tests in (B) (graph on the right), (C), (D), (F), (G) (graph in the middle), (H), (I), and (J); and an unpaired $t$ test with Welch's correction in (G) (graph on the right). See also Figure S9.

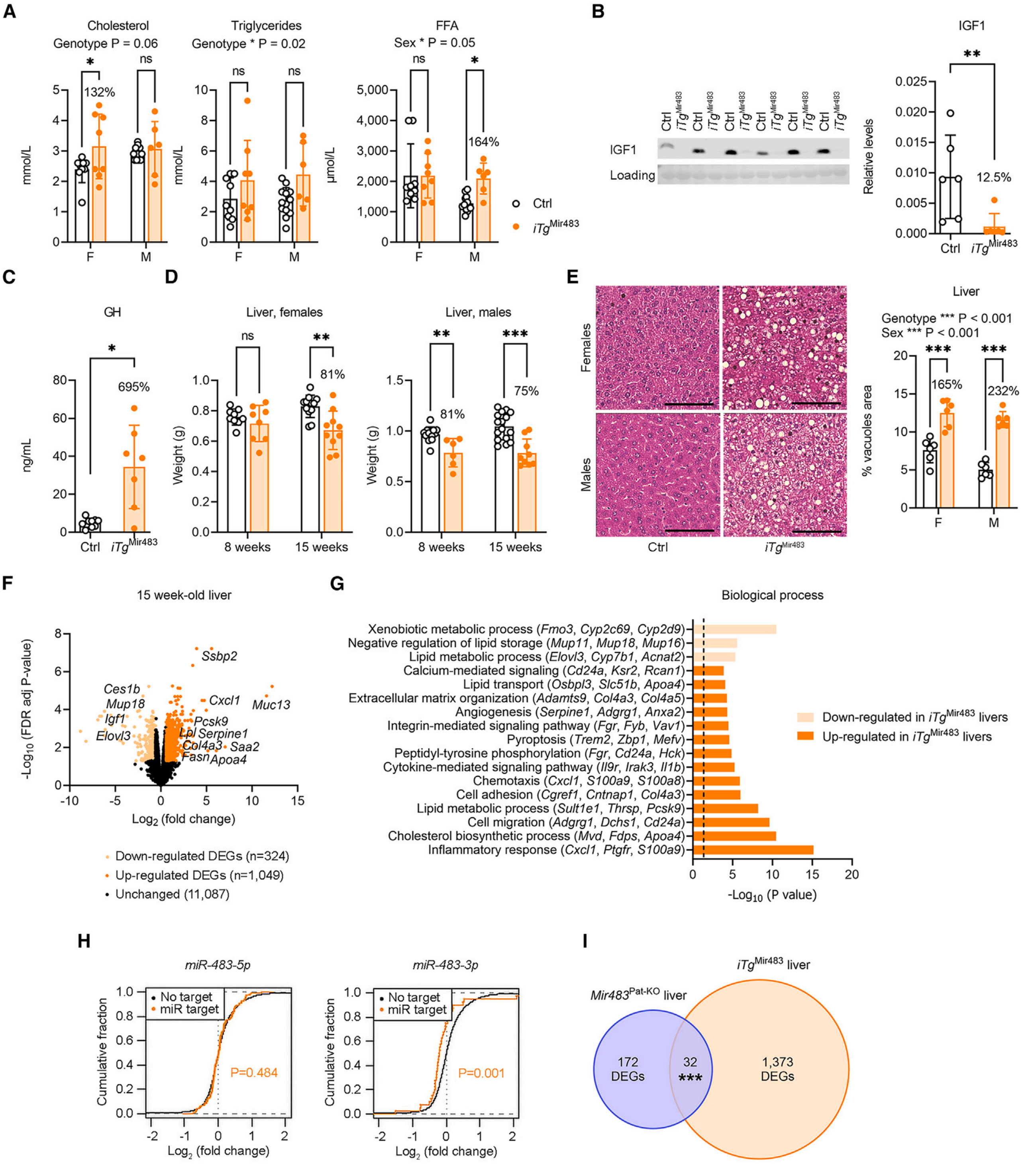

**Figure 6. *iTg*$^{Mir483}$ mice show mild dyslipidemia, altered IGF1 and GH levels, and increased lipid production in the liver**

(A) Serum lipid biochemistry showing moderate dyslipidemia in W8 *iTG*$^{Mir483}$ adults compared to age-matched controls (FFA, free fatty acids; *n* = 6–13 per group).

(B) Circulating plasma IGF1 levels are severely reduced in W15 *iTG*$^{Mir483}$ adults compared to age-matched controls (left, western blotting; right, quantification; *n* = 5 per group).

(C) Circulating plasma GH levels are increased in W15 *iTG*$^{Mir483}$ adults compared to age-matched controls (measurements done by ELISA in *n* = 7–9 per group).

(D) Livers are significantly lighter in W8 and W15 *iTG*$^{Mir483}$ adults compared to age-matched controls (*n* = 6–16 per group).

*(legend continued on next page)*

increased lipid deposition in adipocytes and liver steatosis is currently unknown. Our data suggest that the uptake of lipids in adipocytes may be secondary to the release of excess lipids produced by the liver into the circulation. This is supported by molecular markers of increased lipid production and lipolysis in the liver, but not in the adipose tissue, and the identification of a strong signature of altered lipid metabolism in adult livers of *iTg*$^{Mir483}$ by transcriptomics. Enrichment in functional pathways related to lipid metabolism were also observed in the transcriptome analyses of fetal liver from *Mir483*$^{Pat\text{-}KO}$ and E10.5 overexpressing *Mir483*$^{5C}$ mice, further suggesting that *Mir483* has a modulator role in metabolic function at the whole organism level.

IGF1 is unlikely to play a role in mature adipocyte metabolism, as when pre-adipocytes differentiate they stop expressing IGF1R[47] (thus, mature adipocytes are not a direct target for IGF1 actions, but they secrete IGF1). The differences observed in adipocyte size were independent of GDF3, a direct target of *miR-483-3p*.[21] Identification of additional targets of *miR-483* in the adipose tissue warrants future studies. In humans, *miR-483-5p* is positively correlated with body mass index (BMI), waist circumference, and triglyceride levels and negatively correlated with HDL cholesterol and is a predictive factor for the development of type 2 diabetes mellitus and atherosclerosis.[49] *miR-483-3p* is significantly upregulated in type 2 diabetes mellitus and cardiovascular diseases and has been shown to induce apoptosis and lipotoxicity across various cell types.[50]

The effect of *Mir483* overexpression in inhibiting both IGF2 in pre-natal development and IGF1 in pre-natal and post-natal life, leading to growth suppression, would predict that loss of function of *Mir483* might have the opposite effect. However, the knockout of *Mir483* was viable and showed normal growth, body composition, and glucose homeostasis, as well as normal levels of IGF1 and IGF2 pre-natally and post-natally. The explanation for the lack of *Igf1* and *Igf2* effects when *Mir483* is absent, and more generally why there are no obvious phenotypic consequences, may lie in redundant functions of other related miRs and/or complex regulatory network buffering that enable maintenance of homeostasis despite a defective node in the network. Accordingly, functional redundancy among miRs has been shown in several studies, ranging from mice to worms.[51–53] Our miR-seq analysis in the E18.5 liver of the *Mir483* knockout identified a small number of DEMs, with a significant impact on the mRNA changes measured by RNA-seq. These miRs include the imprinted miR at the DLK/GTL2 locus (*miR-136-5p*), two *let-7* miRs (*let-7f-5p* and *let-7d-3p*) that are potential targets of the non-coding imprinted *H19* gene,[54] and *miR-370-3p*. Notably, INS-IGF signaling pathway genes are potential targets of these four DEMs. Whether their action is responsible for the absence of elevated levels of IGF1 and IGF2 in the *Mir483* knockout requires further examination. Alternative explanations for the absence of a phenotype in the *Mir483* knockout include *Mir483* being sequestered by miR sponges during normal development, e.g., RNA binding proteins such as IGF2BPs or mRNA transcripts within the *H19*/*Igf2* locus, or that *Mir483* competes for binding to *Igf2* mRNA (IGF2BP binding sites in the 5′ and 3′ UTRs overlap with *Mir483* seed targets). In support of this hypothesis, we note that it is only when fetuses lack both *Igf2* and *Mir483* that IGF1 is elevated (as shown for the fetal liver).

Our results contradict previous observations made *in vitro* in human Ewing sarcoma cells[18] and immortalized mouse myoblast cells,[55] which suggested a positive feedback loop between *Mir483* and *Igf2* transcription. These results imply either cell-type-specific effects of *Mir483* on *Igf2* regulation or the involvement of additional regulatory layers *in vivo* (unique to our study, the -3p and -5p levels were altered throughout the entire development). More generally, conflicting data have been reported in relation to the *Mir483* roles in the context of cancer (i.e., oncomir versus oncosuppressor). A recent study[56] uncovered a potential mechanism by which an miR can produce opposite effects based on differential gene target expression levels, which provides new insights on the complexity of miR actions when trying to establish physiological roles.

Despite the highlighted differences between mouse and human *Igf2*/*Mir483* regulation, our work has potential relevance to human imprinting syndromes, cancer, and genomic imprinting in general. In our study, we provide mechanistic insights into *Mir483* regulation *in vivo* in mice: *Mir483* expression is entirely dependent on *Igf2* transcriptional units and is under the hierarchical control of the gametic ICR1, as shown by using the *Igf2*$^{\Delta(P1\text{-}P3)}$ and *H19*$^{\Delta 13/+}$ deletion models, respectively. Children with the intrauterine and post-natal growth retardation SRS that carry DNA hypomethylation epimutations of the ICR1 have decreased levels of IGF2 and increased levels of IGF1 and IGFBP3.[57] The increase in *Igf1*/IGF1 might be caused by the downregulation of *miR-483* and *IGF2*, as observed in our SRS mouse models, *Igf2*$^{Pat\text{-}KO}$ and *Igf2*$^{+/LacZ}$, with elevated IGF1 in fetal liver. Conversely, the overgrowth observed in BWS[58] is modeled in this study by the *H19*$^{\Delta 13/+}$ mice, which also associate elevated *Mir483* and *Igf2* levels. Overall, the roles played by

(E) Livers of W8 *iTG*$^{Mir483}$ adults show significant accumulation of vacuoles, suggestive of liver steatosis (left, representative H&E-stained sections, and right, quantification of percentage vacuoles area; $n = 6$ samples per group; scale bars, 100 μm).

(F) Volcano plot representation of DEGs identified by RNA-seq in 15-week-old livers (*iTG*$^{Mir483}$ versus Ctrl). Significant downregulated and upregulated DEGs (FDR < 0.05, fold change >1.5) are shown with light and dark orange dots, respectively. See also Table S6.

(G) Top-scoring biological processes enriched in DEGs identified by DAVID analysis. Three DEGs with highest fold changes are listed in parentheses. The dotted line corresponds to FDR-corrected *p* value of 0.05. See also Table S6.

(H) Cumulative fractions mRNA fold changes between *iTG*$^{Mir483}$ and Ctrl livers for putative targets of *miR-483-5p* (left) and *miR-483-3p* (right). Statistical differences between distributions were calculated using two-sided Kolmogorov-Smirnov tests.

(I) Venn diagram depicting DEGs identified as common in the livers of loss-of-function (*Mir483*$^{Pat\text{-}KO}$) and gain-of-function (*iTG*$^{Mir483}$) mouse models using RNA-seq analyses. See also Table S6.

Data are presented as individual values with averages ± SD in (A), (B), (C), (D), and (E), and percentage or fold (x) values indicate ratios of *iTG*$^{Mir483}$/Ctrl; ns, non-significant; *$p < 0.05$, **$p < 0.01$, and ***$p < 0.001$ by two-way ANOVA followed by Šídák's multiple comparisons tests in (A), (D), and (E); Mann-Whitney tests in (B) and (C); and chi-squared with Yates' correction in (I). See also Figures S10 and S11.

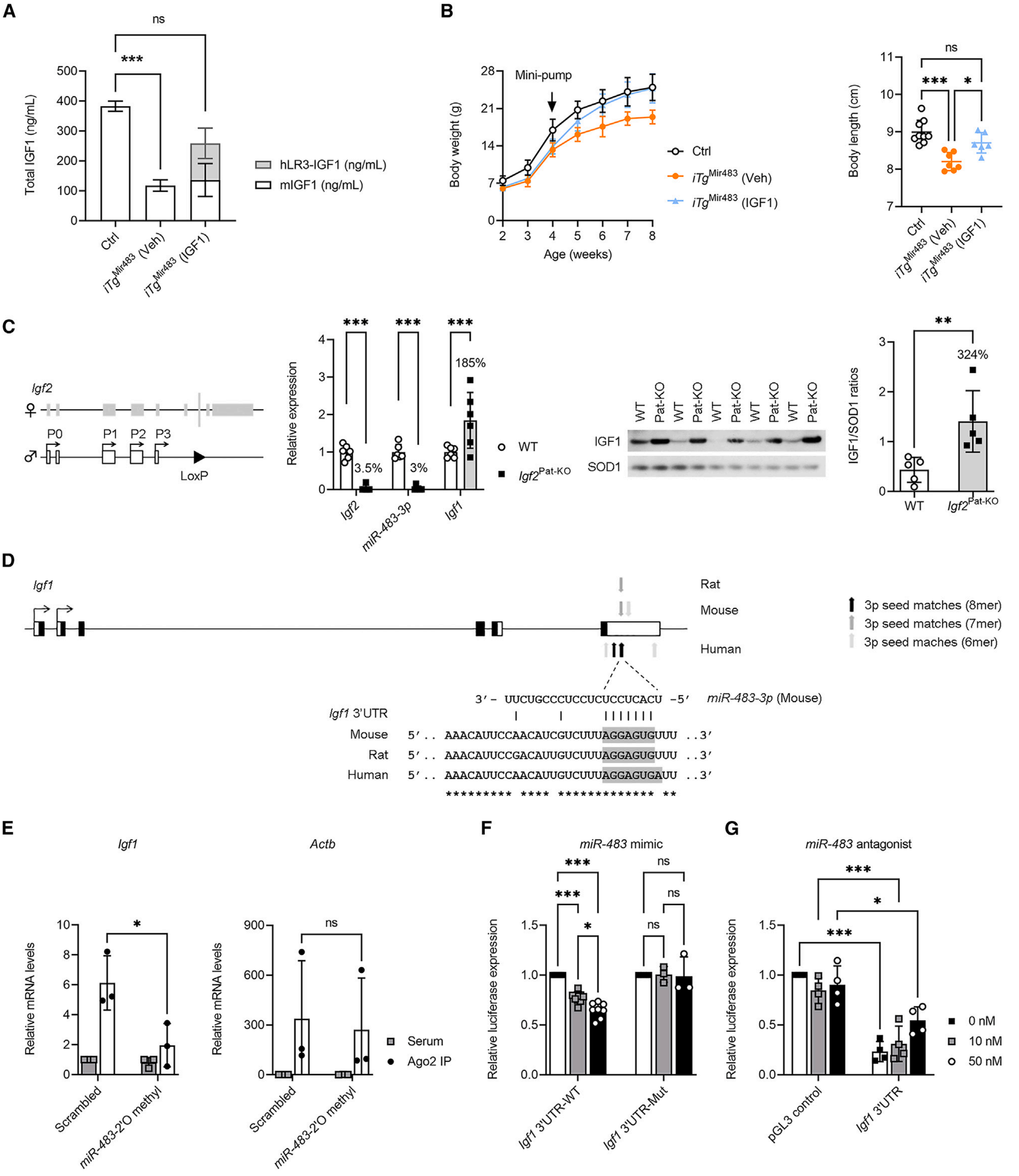


**Figure 7. Infusing IGF1 rescues growth phenotypes in *iTg*$^{\mathrm{Mir483}}$ mice, and IGF1 is a target of *miR-483-3p* both *in vitro* and *in vivo***

(A) IGF1 infusion via minipump restores the low levels of IGF1 observed in *iTg*$^{\mathrm{Mir483}}$ mice. Measurements were performed in plasma of W6 male mice 2 weeks after the surgery; *n* = 6–7 mice per group.

(B) IGF1 infusion normalizes body weight (left) and body length (right) (*n* = 6–9 male mice per group).

(C) Left: schematic representation of the *Igf2*$^{\mathrm{Pat\text{-}KO}}$ model in which the coding exons 4–6 of *Igf2* and *Mir483* are deleted using *Cre*-recombinase. Middle: relative RNA levels for *Igf2*, *miR-483-3p*, and *Igf1* in the liver of E18.5 *Igf2*$^{\mathrm{Pat\text{-}KO}}$ mutants and WT littermate controls (data were normalized against the geometrical means

(legend continued on next page)

*MIR483* in these two human imprinting syndromes remain unclear.

Several imprinted miRs play important roles in placenta, including *Mir675*, which also maps to the *Igf2*/*H19* domain. Mouse studies indicate that processing of *miR-675* from the non-coding *H19* RNA acts to limit placental growth through IGF1R repression.[16] Intriguingly, as discussed previously, *Mir483* does not seem to play a role in controlling placental growth. It is interesting, however, that the two miRs, *Mir483* and *Mir675*, in this imprinted domain converge their actions on the IGF signaling system to regulate growth—*miR-483* on IGF2 and IGF1 and *miR-675* on IGF1R—the receptor for IGF1 and IGF2. In both cases, they act developmentally as growth suppressors. Uncoupling of *MIR483* from *IGF2* expression seems to have particular importance in human pathology and can be observed in response to environmental cues.[59] For example, *miR-483-3p* is upregulated in the adipose tissue of low-birth-weight adult humans and pre-diabetic adult rats exposed to suboptimal nutrition in early life.[21] In addition, *miR-483-5p* upregulation in the amygdala of male mice exposed to stress promoted a reduction in anxiety-like behavior.[60] Therefore, in future studies, the exposure of *Mir483*$^{\text{Pat-KO}}$ mice to environmental or physiological stressors that lead to uncoupling its expression from that of *Igf2* may uncover novel cellular, molecular, and metabolic phenotypes that are *Mir483* dependent.

Our findings appear to contradict the conflict theory of the evolution of imprinting in the sense that *Mir483* is a paternally expressed miR acting as growth suppressor. It is, however, possible that imprinting of *Mir483* occurred as a "bystander" product of the evolution of the ICR1. Under this hypothesis, the silencing of the maternal *Igf2* allele via the ICR1 had the effect of restricting *Mir483* growth-suppressing activity on the paternal allele only. In support of this hypothesis, we show that expression of *Mir483* is dependent on ICR1 and note that *Mir483* sequences are not conserved in monotremes, suggesting that its appearance coincided with that of imprinting regulation.

In summary, the combination of loss-of-function and gain-of-function mouse models reported in this study demonstrates that *Mir483* is an imprinted miR, coexpressed from the paternal allele with its host *Igf2* gene during mouse development. We suggest that *Mir483* was coopted as a growth and metabolic regulator in a region containing *INS-IGF2* genes, generating a new use for an existing transcriptional unit by changing patterns of gene regulation (e.g., acting in *cis* on *IGF2* and in *trans* on *IGF1*). The discovery that *Mir483* may buffer the levels of two major growth factors, IGF2 and IGF1, supports the concept that this miR was evolutionarily selected to prevent excessive deviations from normal patterns of growth. Moreover, based on our findings in mice, it is possible that in certain cancer contexts, *Mir483* could be used as a therapeutic agent to delay or prevent tumor growth.

## Limitations of the study

We have not performed a comprehensive analysis of the *Mir483* imprinting status in cells and tissues across the life course. Therefore, we cannot exclude that *Mir483* is expressed biallelically in certain cell types and that deletion of both alleles is required for complete reduction in levels, as suggested by others in pancreatic beta cells.[26] The levels of *Mir483* in *iTg*$^{\text{Mir483}}$ and *Mir483*$^{\text{5C}}$ are supraphysiological, as is commonly the case in most studies overexpressing miRs in cell lines. High levels of *Mir483* are needed to achieve robust uncoupling of *Mir483* from *Igf2* transcription and are required to unmask target genes and elicit physiological responses *in vivo* in the absence of environmental challenges. Indeed, CAG-driven transgenics with lower levels of overexpression than those reported here did not cause phenotypes unless challenged with a toxin.[61] Furthermore, our findings that similar levels of overexpression in placenta and fetus only cause growth phenotypes in the fetus speak against unspecific phenotype effects. The post-natal expression of *Mir483* in the *iTg*$^{\text{Mir483}}$ transgenic mice is driven by the *Rosa26* promoter, which is aimed at mimicking the ubiquitous expression of the *MIR483* observed in human adults, but levels and sites of expression are likely to differ from the endogenous locus. We did not determine the extent to which known *miR-483* targets previously reported in adipocytes,[21] myoblasts,[41] beta cells,[25,26] hepatocytes,[62] and basolateral amygdala[60] are altered in our inducible model. Like others,[18] we were unable to validate the IGF2 target sequences for *Mir483* regulation using traditional luciferase reporter experiments. However, experimental data from others[18,55] using non-reporter-based methods, and the *in vivo* data herein reported, demonstrate that IGF2 is highly likely to be a target of *Mir483*. Comparative

of *Ppia*, *Pmm1*, and *Hprt*, for *Igf2* and *Igf1*, and *Snord70*/*snoRNA234* and *Snord68*/*snoRNA202* for *miR-483-3p*; *n* = 6 samples per group). Right: western blot of IGF1 protein in the liver of E18.5 *Igf2*$^{\text{Pat-KO}}$ mutants and WT littermate controls, normalized to SOD1 (*n* = 5 per group).

(D) Diagram of the mouse *Igf1* locus showing location of putative *miR-483-3p* seed sequences and their corresponding locations in the rat and human. The sequence alignment corresponds to the *miR-483-3p* seed-containing sequence in the *Igf1* 3′ UTR that is conserved across all three species (*miR-483-3p* target sequences are highlighted in gray).

(E) Undifferentiated 3T3-L1 cells were transfected with a *miR-483-3p* 2′-O-methyl antagonist. Ago2 protein immunoprecipitation was performed, and total RNA was collected. RT-qPCR was used to assay the levels of *Igf1* and *Actb* mRNA present in the immunoprecipitated RNA-induced silencing complex (RISC) (*n* = 3 independent experiments).

(F) HEK-293 cells (expressing low levels of endogenous *MIR483*) were cotransfected with luciferase reporter constructs containing a portion of the 3′ UTR of mouse *Igf1* mRNA spanning the *miR-483-3p* seed target region or mutated seed sequence, together with 0, 10, or 50 nM mouse *miR-483-3p* mimic (*n* = 3–8 samples per group).

(G) HepG2 cells (expressing high endogenous levels of *MIR483*) were cotransfected with luciferase reporter constructs containing a portion of the 3′ UTR of mouse *Igf1* mRNA spanning the *miR-483-3p* seed target region, together with 0, 50, or 100 nM *miR-483-3p* 2′-O-methyl miR antagonist (*n* = 4 samples per group). Genomic features shown in (C) and (D) are not drawn to scale and are for representation purposes only. Data are averages ± SD in (A) and (B) (left) and individual values with averages ± SD in (B) (right), (C), (E), (F), and (G), and percentage values indicate ratios of *Igf2*$^{\text{Pat-KO}}$/WT in (C); ns, non-significant; **p* < 0.05, ***p* < 0.01, and ****p* < 0.001 by Kruskal-Wallis test with Dunn's multiple comparisons tests in (A); one-way ANOVA with Tukey's multiple comparisons test in (B); two-way ANOVA followed by Šídák's multiple comparisons tests in (C) (middle), (E), (F), and (G); or Mann-Whitney test in (C) (right). See also Figure S12.

transcriptomic and proteomic studies in our knockout and overexpression models, collected at the same developmental embryonic and post-natal time points, would be required to understand *Mir483*-centered developmental networks and the mechanisms of functional redundancy/buffering networks.

## RESOURCE AVAILABILITY

### Lead contact

Requests for further information and resources and reagents should be directed to and will be fulfilled by the lead contact, Miguel Constância (jmasmc2@cam.ac.uk).

### Materials availability

Novel mouse models generated in this study will be made available upon reasonable request.

### Data and code availability

- All data are provided with the article. The RNA-seq data were deposited in the Gene Expression Omnibus (GEO) repository under accession nos. GSE256302, GSE256304, GSE256305, and GSE256306 (https://www.ncbi.nlm.nih.gov/gds).
- The TMT-mass spectrometry proteomics data were deposited to the ProteomeXchange Consortium (http://proteomecentral.proteomexchange.org), via the PRIDE partner repository, with the dataset identifier PXD051516.
- The custom codes used in this study are available in Zenodo for RNA-seq analyses and for cumulative fraction analyses. DOIs are listed in the key resources table.
- Any additional information required to reanalyze the data reported in this work is available from the lead contact upon request.

## ACKNOWLEDGMENTS

We thank Laura Hunter and Claire Custance (West Forvie Phenomics Center) for help with mouse husbandry; Debbie Drage, Martin George, and, in particular, Ted Saunders (The Babraham Institute Gene Targeting Facility) for help with generating the *Igf2*$^{\Delta(P1\text{-}P3)}$ mice; James Warner and Katherine Vickers (Histopathology Core) for help with preparing tissue samples for histology; Gregory Strachan (Imaging Core) for help with confocal microscopy and cell counting/measurements using HALO; Keith Burling and Peter Barker (Core Biochemistry Assay Lab) for performing blood biochemistry measurements; Amy Warner and Sarah Grocott (Disease Model Core) for osmotic minipump implantation through surgery; Dr. Paúl Cordero for help with mRNA expression analyses; Dr. Tim Mohun (Crick Institute, London) for help with tissue collection for HREM; Prof. Abigail Fowden for help with mouse licensing approval for part of the work on the *iTg*$^{Mir483}$ model; and Dr. Allan Bradley for providing *Mir483*$^{KO}$ ES cells. This work was supported by the Medical Research Council (MR/J001562/1 to M.C.; MRC_MC_UU_12014/4 to M.C. and S.E.O.; MRC_MC_UU_12012/5 to the MRC Metabolic Diseases Unit; MR/M009041/1 – "Enhancing UK Clinical Research" grant supporting the TQ-XS work), the Biotechnology and Biological Sciences Research Council (BBSRC BB/F014279/2 to A.E.W. and M.B.), and Cancer Research UK (CRUK Institute award A29252 to M.B.); N.C. was funded by the Frank Edward Elmore Fund, the Association of Physicians of Great Britain & Ireland and the Anatomical Society; Y.S. was funded by post-doctoral fellowships from the Uehara Memorial Foundation and the Japan Society for the Promotion of Science; D.C. was funded by the Erasmus Placement Programme; and K.B.d.Q. was funded by Coordenação de Aperfeiçoamento de Pessoal de Nível Superior, CAPES, Brazil. Schematic representations shown in Figures S9G and S10C–S10E and the graphical abstract were generated using BioRender (Biorender.com).

## AUTHOR CONTRIBUTIONS

Conceptualization, K.S., A.E.W., M.B., S.E.O., and M.C.; methodology, I.S., D.S.F.-T., Y.S., N.C., W.N.C., I.Z., L.C.P., R.G.K., S.H.G., L.F.R., W.J.W., D.F.-M., and B.Y.H.L.; investigation, *Mir483*$^{KO}$, I.S., D.S.F.-T., Y.S., D.C., N.C., I.Z., H.M.P., L.C.P., K.B.d.Q., M.K.L.M., and B.Y.H.L.; *H19*$^{\Delta 13KO}$, Y.S. and D.S.F.-T.; *Igf2*$^{KOs}$, I.S., D.S.F.-T., K.H., and N.H.S.; *Mir483*$^{5C}$, W.N.C., I.Z., C.S.K.C., N.M.S., and R.A.; *iTg*$^{Mir483}$, I.S., D.S.F.-T., N.C., W.N.C., I.Z., K.B.d.Q., L.M.O., L.C.P., R.G.K., M.K.L.M., S.H.G., L.F.R., W.J.W., and B.Y.H.L.; cellular *Mir483* assays, D.F.-M. and M.B.; writing – original draft, M.C.; writing – review & editing, I.S., D.S.F.-T., N.C., W.N.C., Y.S., I.Z., D.F.-M, S.G., M.B., and M.C.; resources, S.E.O. and M.C.; supervision, S.E.O. and M.C.

## DECLARATION OF INTERESTS

The authors declare no competing interests.

## STAR★METHODS

Detailed methods are provided in the online version of this paper and include the following:

- KEY RESOURCES TABLE
- EXPERIMENTAL MODEL AND STUDY PARTICIPANT DETAILS
  - Mice
  - Generation of the *Igf2*$^{\Delta(P1\text{-}P3)}$ mouse model
  - Generation of a *Mir483* specific knockout mouse
  - Generation of *Mir483*$^{5C}$ mice with five copies of *Mir483* at the endogenous locus
  - Generation of *iTg*$^{Mir483}$ mice with an additional copy of *Mir483* inserted at the *Rosa26* locus
  - Additional mouse strains and mouse husbandry
- METHOD DETAILS
  - Nomenclature
  - Southern blotting
  - Northern blotting
  - Sequence alignment
  - RNA extraction and RT-qPCR
  - RNA sequencing and bioinformatic analyses
  - Food intake
  - Body composition
  - Histology and stereology analyses
  - Glucose tolerance tests
  - Tandem Mass Tag (TMT) analysis
  - Protein extraction and western blotting
  - IGF1, IGF2 and GH measurements by ELISA
  - Plasma insulin and total-pancreas insulin measurements
  - Blood biochemistry
  - High-resolution episcopic microscopy (HREM)
  - Ago2 immunoprecipitation
  - *In vitro* luciferase assays
  - Continuous IGF1 administration via minipumps
- QUANTIFICATION AND STATISTICAL ANALYSIS

## SUPPLEMENTAL INFORMATION

Supplemental information can be found online at https://doi.org/10.1016/j.celrep.2024.114750.

Received: January 10, 2023
Revised: June 4, 2024
Accepted: August 28, 2024
Published: September 15, 2024

## REFERENCES

1. Bartel, D.P. (2004). MicroRNAs: genomics, biogenesis, mechanism, and function. Cell *116*, 281–297. https://doi.org/10.1016/s0092-8674(04)00045-5.

2. Treiber, T., Treiber, N., and Meister, G. (2019). Regulation of microRNA biogenesis and its crosstalk with other cellular pathways. Nat. Rev. Mol. Cell Biol. *20*, 5–20. https://doi.org/10.1038/s41580-018-0059-1.

3. O'Brien, J., Hayder, H., Zayed, Y., and Peng, C. (2018). Overview of MicroRNA Biogenesis, Mechanisms of Actions, and Circulation. Front. Endocrinol. *9*, 402. https://doi.org/10.3389/fendo.2018.00402.

4. Pritchard, C.C., Cheng, H.H., and Tewari, M. (2012). MicroRNA profiling: approaches and considerations. Nat. Rev. Genet. *13*, 358–369. https://doi.org/10.1038/nrg3198.

5. Condrat, C.E., Thompson, D.C., Barbu, M.G., Bugnar, O.L., Boboc, A., Cretoiu, D., Suciu, N., Cretoiu, S.M., and Voinea, S.C. (2020). miRNAs as Biomarkers in Disease: Latest Findings Regarding Their Role in Diagnosis and Prognosis. Cells *9*, 276. https://doi.org/10.3390/cells9020276.

6. Matsui, M., and Corey, D.R. (2017). Non-coding RNAs as drug targets. Nat. Rev. Drug Discov. *16*, 167–179. https://doi.org/10.1038/nrd.2016.117.

7. Tucci, V., Isles, A.R., Kelsey, G., and Ferguson-Smith, A.C.; Erice Imprinting Group (2019). Genomic Imprinting and Physiological Processes in Mammals. Cell *176*, 952–965. https://doi.org/10.1016/j.cell.2019.01.043.

8. Labialle, S., and Cavaillé, J. (2011). Do repeated arrays of regulatory small-RNA genes elicit genomic imprinting?: Concurrent emergence of large clusters of small non-coding RNAs and genomic imprinting at four evolutionarily distinct eutherian chromosomal loci. Bioessays *33*, 565–573. https://doi.org/10.1002/bies.201100032.

9. Girardot, M., Cavaillé, J., and Feil, R. (2012). Small regulatory RNAs controlled by genomic imprinting and their contribution to human disease. Epigenetics *7*, 1341–1348. https://doi.org/10.4161/epi.22884.

10. Haig, D., and Mainieri, A. (2020). The Evolution of Imprinted microRNAs and Their RNA Targets. Genes *11*, 1038. https://doi.org/10.4161/epi.22884.

11. Malnou, E.C., Umlauf, D., Mouysset, M., and Cavaillé, J. (2018). Imprinted MicroRNA Gene Clusters in the Evolution, Development, and Functions of Mammalian Placenta. Front. Genet. *9*, 706. https://doi.org/10.3389/fgene.2018.00706.

12. Wesseler, K., Kraft, F., and Eggermann, T. (2019). Molecular and Clinical Opposite Findings in 11p15.5 Associated Imprinting Disorders: Characterization of Basic Mechanisms to Improve Clinical Management. Int. J. Mol. Sci. *20*, 4219. https://doi.org/10.3390/ijms20174219.

13. Bergman, D., Halje, M., Nordin, M., and Engström, W. (2013). Insulin-like growth factor 2 in development and disease: a mini-review. Gerontology *59*, 240–249. https://doi.org/10.1159/000343995.

14. Seal, R.L., Chen, L.L., Griffiths-Jones, S., Lowe, T.M., Mathews, M.B., O'Reilly, D., Pierce, A.J., Stadler, P.F., Ulitsky, I., Wolin, S.L., and Bruford, E.A. (2020). A guide to naming human non-coding RNA genes. EMBO J. *39*, e103777. https://doi.org/10.15252/embj.2019103777.

15. Bartolomei, M.S., and Ferguson-Smith, A.C. (2011). Mammalian genomic imprinting. Cold Spring Harb. Perspect. Biol. *3*, a002592. https://doi.org/10.1101/cshperspect.a002592.

16. Keniry, A., Oxley, D., Monnier, P., Kyba, M., Dandolo, L., Smits, G., and Reik, W. (2012). The H19 lincRNA is a developmental reservoir of miR-675 that suppresses growth and Igf1r. Nat. Cell Biol. *14*, 659–665. https://doi.org/10.1038/ncb2521.

17. Veronese, A., Lupini, L., Consiglio, J., Visone, R., Ferracin, M., Fornari, F., Zanesi, N., Alder, H., D'Elia, G., Gramantieri, L., et al. (2010). Oncogenic role of miR-483-3p at the IGF2/483 locus. Cancer Res. *70*, 3140–3149. https://doi.org/10.1158/0008-5472.CAN-09-4456.

18. Liu, M., Roth, A., Yu, M., Morris, R., Bersani, F., Rivera, M.N., Lu, J., Shioda, T., Vasudevan, S., Ramaswamy, S., et al. (2013). The IGF2 intronic miR-483 selectively enhances transcription from IGF2 fetal promoters and enhances tumorigenesis. Genes Dev. *27*, 2543–2548. https://doi.org/10.1101/gad.224170.113.

19. Wang, L., Shi, M., Hou, S., Ding, B., Liu, L., Ji, X., Zhang, J., and Deng, Y. (2012). MiR-483-5p suppresses the proliferation of glioma cells via directly targeting ERK1. FEBS Lett. *586*, 1312–1317. https://doi.org/10.1016/j.febslet.2012.03.035.

20. Loo, J.M., Scherl, A., Nguyen, A., Man, F.Y., Weinberg, E., Zeng, Z., Saltz, L., Paty, P.B., and Tavazoie, S.F. (2015). Extracellular metabolic energetics can promote cancer progression. Cell *160*, 393–406. https://doi.org/10.1016/j.cell.2014.12.018.

21. Ferland-McCollough, D., Fernandez-Twinn, D.S., Cannell, I.G., David, H., Warner, M., Vaag, A.A., Bork-Jensen, J., Brøns, C., Gant, T.W., Willis, A.E., et al. (2012). Programming of adipose tissue miR-483-3p and GDF-3 expression by maternal diet in type 2 diabetes. Cell Death Differ. *19*, 1003–1012. https://doi.org/10.1038/cdd.2011.183.

22. Bertero, T., Gastaldi, C., Bourget-Ponzio, I., Imbert, V., Loubat, A., Selva, E., Busca, R., Mari, B., Hofman, P., Barbry, P., et al. (2011). miR-483-3p controls proliferation in wounded epithelial cells. Faseb. J. *25*, 3092–3105. https://doi.org/10.1096/fj.10-168401.

23. Qiao, Y., Zhao, Y., Liu, Y., Ma, N., Wang, C., Zou, J., Liu, Z., Zhou, Z., Han, D., He, J., et al. (2016). miR-483-3p regulates hyperglycaemia-induced cardiomyocyte apoptosis in transgenic mice. Biochem. Biophys. Res. Commun. *477*, 541–547. https://doi.org/10.1016/j.bbrc.2016.06.051.

24. Clokie, S.J.H., Lau, P., Kim, H.H., Coon, S.L., and Klein, D.C. (2012). MicroRNAs in the pineal gland: miR-483 regulates melatonin synthesis by targeting arylalkylamine N-acetyltransferase. J. Biol. Chem. *287*, 25312–25324. https://doi.org/10.1074/jbc.M112.356733.

25. Mohan, R., Mao, Y., Zhang, S., Zhang, Y.W., Xu, C.R., Gradwohl, G., and Tang, X. (2015). Differentially Expressed MicroRNA-483 Confers Distinct Functions in Pancreatic β- and α-Cells. J. Biol. Chem. *290*, 19955–19966. https://doi.org/10.1074/jbc.M115.650705.

26. Wang, Z., Mohan, R., Chen, X., Matson, K., Waugh, J., Mao, Y., Zhang, S., Li, W., Tang, X., Satin, L.S., and Tang, X. (2021). microRNA-483 Protects Pancreatic β-Cells by Targeting ALDH1A3. Endocrinology *162*, bqab031. https://doi.org/10.1210/endocr/bqab031.

27. Kuschnerus, K., Straessler, E.T., Müller, M.F., Lüscher, T.F., Landmesser, U., and Kränkel, N. (2019). Increased Expression of miR-483-3p Impairs the Vascular Response to Injury in Type 2 Diabetes. Diabetes *68*, 349–360. https://doi.org/10.2337/db18-0084.

28. Zhang, J., Zhang, F., Didelot, X., Bruce, K.D., Cagampang, F.R., Vatish, M., Hanson, M., Lehnert, H., Ceriello, A., and Byrne, C.D. (2009). Maternal high fat diet during pregnancy and lactation alters hepatic expression of insulin like growth factor-2 and key microRNAs in the adult offspring. BMC Genom. *10*, 478. https://doi.org/10.1186/1471-2164-10-478.

29. Emmerling, V.V., Fischer, S., Stiefel, F., Holzmann, K., Handrick, R., Hesse, F., Hörer, M., Kochanek, S., and Otte, K. (2016). Temperature-sensitive miR-483 is a conserved regulator of recombinant protein and viral vector production in mammalian cells. Biotechnol. Bioeng. *113*, 830–841. https://doi.org/10.1002/bit.25853.

30. Leighton, P.A., Ingram, R.S., Eggenschwiler, J., Efstratiadis, A., and Tilghman, S.M. (1995). Disruption of imprinting caused by deletion of the H19 gene region in mice. Nature *375*, 34–39. https://doi.org/10.1038/375034a0.

31. Veronese, A., Visone, R., Consiglio, J., Acunzo, M., Lupini, L., Kim, T., Ferracin, M., Lovat, F., Miotto, E., Balatti, V., et al. (2011). Mutated beta-catenin evades a microRNA-dependent regulatory loop. Proc. Natl. Acad. Sci. USA *108*, 4840–4845. https://doi.org/10.1073/pnas.1101734108.

32. Honecker, J., Ruschke, S., Seeliger, C., Laber, S., Strobel, S., Pröll, P., Nellaker, C., Lindgren, C.M., Kulozik, U., Ecker, J., et al. (2022). Transcriptome and fatty-acid signatures of adipocyte hypertrophy and its non-invasive MR-based characterization in human adipose tissue. EBioMedicine *79*, 104020. https://doi.org/10.1016/j.ebiom.2022.104020.

33. Soukas, A., Cohen, P., Socci, N.D., and Friedman, J.M. (2000). Leptin-specific patterns of gene expression in white adipose tissue. Genes Dev. *14*, 963–980. https://doi.org/10.1101/gad.14.8.963.

34. Hammerle, C.M., Sandovici, I., Brierley, G.V., Smith, N.M., Zimmer, W.E., Zvetkova, I., Prosser, H.M., Sekita, Y., Lam, B.Y.H., Ma, M., et al. (2020). Mesenchyme-derived IGF2 is a major paracrine regulator of pancreatic growth and function. PLoS Genet. *16*, e1009069. https://doi.org/10.1371/journal.pgen.1009069.

35. Murrell, A., Heeson, S., Bowden, L., Constância, M., Dean, W., Kelsey, G., and Reik, W. (2001). An intragenic methylated region in the imprinted Igf2 gene augments transcription. EMBO Rep. *2*, 1101–1106. https://doi.org/10.1093/embo-reports/kve248.

36. Rother, K.I., and Accili, D. (2000). Role of insulin receptors and IGF receptors in growth and development. Pediatr. Nephrol. *14*, 558–561. https://doi.org/10.1007/s004670000351.

37. LeRoith, D., Holly, J.M.P., and Forbes, B.E. (2021). Insulin-like growth factors: Ligands, binding proteins, and receptors. Mol. Metabol. *52*, 101245. https://doi.org/10.1016/j.molmet.2021.101245.

38. Sélénou, C., Brioude, F., Giabicani, E., Sobrier, M.L., and Netchine, I. (2022). *IGF2*: Development, Genetic and Epigenetic Abnormalities. Cells *11*, 1886. https://doi.org/10.3390/cells11121886.

39. Han, L., Luo, Q.Q., Peng, M.G., Zhang, Y., and Zhu, X.H. (2021). miR-483 is downregulated in pre-eclampsia via targeting insulin-like growth factor 1 (IGF1) and regulates the PI3K/Akt/mTOR pathway of endothelial progenitor cells. J. Obstet. Gynaecol. Res. *47*, 63–72. https://doi.org/10.1111/jog.14412.

40. Ni, F., Sun, R., Fu, B., Wang, F., Guo, C., Tian, Z., and Wei, H. (2013). IGF-1 promotes the development and cytotoxic activity of human NK cells. Nat. Commun. *4*, 1479. https://doi.org/10.1038/ncomms2484.

41. Song, C., Yang, Z., Dong, D., Xu, J., Wang, J., Li, H., Huang, Y., Lan, X., Lei, C., Ma, Y., and Chen, H. (2019). miR-483 inhibits bovine myoblast cell proliferation and differentiation via IGF1/PI3K/AKT signal pathway. J. Cell. Physiol. *234*, 9839–9848. https://doi.org/10.1002/jcp.27672.

42. Baker, J., Liu, J.P., Robertson, E.J., and Efstratiadis, A. (1993). Role of insulin-like growth factors in embryonic and postnatal growth. Cell *75*, 73–82. https://doi.org/10.1016/S0092-8674(05)80085-6.

43. Sferruzzi-Perri, A.N., Sandovici, I., Constância, M., and Fowden, A.L. (2017). Placental phenotype and the insulin-like growth factors: resource allocation to fetal growth. J. Physiol. *595*, 5057–5093. https://doi.org/10.1113/JP273330.

44. Haluzik, M., Yakar, S., Gavrilova, O., Setser, J., Boisclair, Y., and LeRoith, D. (2003). Insulin resistance in the liver-specific IGF-1 gene-deleted mouse is abrogated by deletion of the acid-labile subunit of the IGF-binding protein-3 complex: relative roles of growth hormone and IGF-1 in insulin resistance. Diabetes *52*, 2483–2489. https://doi.org/10.2337/diabetes.52.10.2483.

45. Musarò, A., McCullagh, K.J., Naya, F.J., Olson, E.N., and Rosenthal, N. (1999). IGF-1 induces skeletal myocyte hypertrophy through calcineurin in association with GATA-2 and NF-ATc1. Nature *400*, 581–585. https://doi.org/10.1038/23060.

46. Yoshida, T., and Delafontaine, P. (2020). Mechanisms of IGF-1-Mediated Regulation of Skeletal Muscle Hypertrophy and Atrophy. Cells *9*, 1970. https://doi.org/10.3390/cells9091970.

47. Shimizu, M., Torti, F., and Roth, R.A. (1986). Characterization of the insulin and insulin-like growth factor receptors and responsitivity of a fibroblast/adipocyte cell line before and after differentiation. Biochem. Biophys. Res. Commun. *137*, 552–558. https://doi.org/10.1016/0006-291x(86)91246-5.

48. Boucher, J., Softic, S., El Ouaamari, A., Krumpoch, M.T., Kleinridders, A., Kulkarni, R.N., O'Neill, B.T., and Kahn, C.R. (2016). Differential Roles of Insulin and IGF-1 Receptors in Adipose Tissue Development and Function. Diabetes *65*, 2201–2213. https://doi.org/10.2337/db16-0212.

49. Gallo, W., Ottosson, F., Kennbäck, C., Jujic, A., Esguerra, J.L.S., Eliasson, L., and Melander, O. (2021). Replication study reveals miR-483-5p as an important target in prevention of cardiometabolic disease. BMC Cardiovasc. Disord. *21*, 162. https://doi.org/10.1186/s12872-021-01964-0.

50. Matson, K., Macleod, A., Mehta, N., Sempek, E., and Tang, X. (2023). Impacts of MicroRNA-483 on Human Diseases. Noncoding. RNA *9*, 37. https://doi.org/10.3390/ncrna9040037.

51. Miska, E.A., Alvarez-Saavedra, E., Abbott, A.L., Lau, N.C., Hellman, A.B., McGonagle, S.M., Bartel, D.P., Ambros, V.R., and Horvitz, H.R. (2007). Most Caenorhabditis elegans microRNAs are individually not essential for development or viability. PLoS Genet. *3*, e215. https://doi.org/10.1371/journal.pgen.0030215.

52. Park, C.Y., Choi, Y.S., and McManus, M.T. (2010). Analysis of microRNA knockouts in mice. Hum. Mol. Genet. *19*, R169–R175. https://doi.org/10.1093/hmg/ddq367.

53. Park, C.Y., Jeker, L.T., Carver-Moore, K., Oh, A., Liu, H.J., Cameron, R., Richards, H., Li, Z., Adler, D., Yoshinaga, Y., et al. (2012). A resource for the conditional ablation of microRNAs in the mouse. Cell Rep. *1*, 385–391. https://doi.org/10.1016/j.celrep.2012.02.008.

54. Zhang, L., Yang, Z., Huang, W., and Wu, J. (2019). H19 potentiates let-7 family expression through reducing PTBP1 binding to their precursors in cholestasis. Cell Death Dis. *10*, 168. https://doi.org/10.1038/s41419-019-1423-6.

55. Naboulsi, R., Larsson, M., Andersson, L., and Younis, S. (2021). ZBED6 regulates Igf2 expression partially through its regulation of miR483 expression. Sci. Rep. *11*, 19484. https://doi.org/10.1038/s41598-021-98777-0.

56. Svoronos, A.A., Campbell, S.G., and Engelman, D.M. (2021). MicroRNA function can be reversed by altering target gene expression levels. iScience *24*, 103208. https://doi.org/10.1016/j.isci.2021.103208.

57. Iliev, D.I., Kannenberg, K., Weber, K., and Binder, G. (2014). IGF-I sensitivity in Silver-Russell syndrome with IGF2/H19 hypomethylation. Growth Hormone IGF Res. *24*, 187–191. https://doi.org/10.1016/j.ghir.2014.06.005.

58. Li, Y., Hagen, D.E., Ji, T., Bakhtiarizadeh, M.R., Frederic, W.M., Traxler, E.M., Kalish, J.M., and Rivera, R.M. (2019). Altered microRNA expression profiles in large offspring syndrome and Beckwith-Wiedemann syndrome. Epigenetics *14*, 850–876. https://doi.org/10.1080/15592294.2019.1615357.

59. Emmerling, V.V., Fischer, S., Kleemann, M., Handrick, R., Kochanek, S., and Otte, K. (2016). miR-483 is a self-regulating microRNA and can activate its own expression via USF1 in HeLa cells. Int. J. Biochem. Cell Biol. *80*, 81–86. https://doi.org/10.1016/j.biocel.2016.09.022.

60. Mucha, M., Skrzypiec, A.E., Kolenchery, J.B., Brambilla, V., Patel, S., Labrador-Ramos, A., Kudla, L., Murrall, K., Skene, N., Dymicka-Piekarska, V., et al. (2023). miR-483-5p offsets functional and behavioural effects of stress in male mice through synapse-targeted repression of Pgap2 in the basolateral amygdala. Nat. Commun. *14*, 2134. https://doi.org/10.1038/s41467-023-37688-2.

61. Li, F., Ma, N., Zhao, R., Wu, G., Zhang, Y., Qiao, Y., Han, D., Xu, Y., Xiang, Y., Yan, B., et al. (2014). Overexpression of miR-483-5p/3p cooperate to inhibit mouse liver fibrosis by suppressing the TGF-b stimulated HSCs in transgenic mice. J. Cell Mol. Med. *18*, 966–974. https://doi.org/10.1111/jcmm.12293.

62. Dong, J., He, M., Li, J., Pessentheiner, A., Wang, C., Zhang, J., Sun, Y., Wang, W.T., Zhang, Y., Liu, J., et al. (2020). microRNA-483 ameliorates hypercholesterolemia by inhibiting PCSK9 production. JCI Insight *5*, e143812. https://doi.org/10.1172/jci.insight.143812.

63. Hooper, M., Hardy, K., Handyside, A., Hunter, S., and Monk, M. (1987). HPRT-deficient (Lesch-Nyhan) mouse embryos derived from germline colonization by cultured cells. Nature *326*, 292-295. https://doi.org/10.1038/326292a0.

64. Prosser, H.M., Koike-Yusa, H., Cooper, J.D., Law, F.C., and Bradley, A. (2011). A resource of vectors and ES cells for targeted deletion of microRNAs in mice. Nat. Biotechnol. *29*, 840–845. https://doi.org/10.1038/nbt.1929.

65. Smith, A.G., and Hooper, M.L. (1987). Buffalo rat liver cells produce a diffusible activity which inhibits the differentiation of murine embryonal

carcinoma and embryonic stem cells. Dev. Biol. *121*, 1–9. https://doi.org/10.1016/0012-1606(87)90132-1.

66. Schwenk, F., Baron, U., and Rajewsky, K. (1995). A cre-transgenic mouse strain for the ubiquitous deletion of loxP-flanked gene segments including deletion in germ cells. Nucleic Acids Res. *23*, 5080–5081. https://doi.org/10.1093/nar/23.24.5080.

67. Matsuda, T., and Cepko, C.L. (2007). Controlled expression of transgenes introduced by in vivo electroporation. Proc. Natl. Acad. Sci. USA *104*, 1027–1032. https://doi.org/10.1073/pnas.0610155104.

68. Raymond, C.S., and Soriano, P. (2007). High-efficiency FLP and PhiC31 site-specific recombination in mammalian cells. PLoS One *2*, e162. https://doi.org/10.1371/journal.pone.0000162.

69. Mao, J., Barrow, J., McMahon, J., Vaughan, J., and McMahon, A.P. (2005). An ES cell system for rapid, spatial and temporal analysis of gene function in vitro and in vivo. Nucleic Acids Res. *33*, e155. https://doi.org/10.1093/nar/gni146.

70. Auyeung, V.C., Ulitsky, I., McGeary, S.E., and Bartel, D.P. (2013). Beyond secondary structure: primary-sequence determinants license pri-miRNA hairpins for processing. Cell *152*, 844–858. https://doi.org/10.1016/j.cell.2013.01.031.

71. Kim, B., Jeong, K., and Kim, V.N. (2017). Genome-wide Mapping of DROSHA Cleavage Sites on Primary MicroRNAs and Noncanonical Substrates. Mol. Cell. *66*, 258–269.e5. https://doi.org/10.1016/j.molcel.2017.03.013.

72. Livak, K.J., and Schmittgen, T.D. (2001). Analysis of relative gene expression data using real-time quantitative PCR and the 2(-Delta Delta C(T)) Method. Methods *25*, 402–408. https://doi.org/10.1006/meth.2001.1262.

73. Sherman, B.T., Hao, M., Qiu, J., Jiao, X., Baseler, M.W., Lane, H.C., Imamichi, T., and Chang, W. (2022). DAVID: a web server for functional enrichment analysis and functional annotation of gene lists (2021 update). Nucleic Acids Res. *50*, W216–W221. https://doi.org/10.1093/nar/gkac194.

74. Supek, F., Bošnjak, M., Škunca, N., and Šmuc, T. (2011). REVIGO summarizes and visualizes long lists of gene ontology terms. PLoS One *6*, e21800. https://doi.org/10.1371/journal.pone.0021800.

75. McGeary, S.E., Lin, K.S., Shi, C.Y., Pham, T.M., Bisaria, N., Kelley, G.M., and Bartel, D.P. (2019). The biochemical basis of microRNA targeting efficacy. Science *366*, eaav1741. https://doi.org/10.1126/science.aav1741.

76. Halldorsdottir, S., Carmody, J., Boozer, C.N., Leduc, C.A., and Leibel, R.L. (2009). Reproducibility and accuracy of body composition assessments in mice by dual energy x-ray absorptiometry and time domain nuclear magnetic resonance. Int. J. Body Compos. Res. *7*, 147–154.

77. Sandovici, I., Hammerle, C.M., Virtue, S., Vivas-Garcia, Y., Izquierdo-Lahuerta, A., Ozanne, S.E., Vidal-Puig, A., Medina-Gómez, G., and Constância, M. (2021). Autocrine IGF2 programmes β-cell plasticity under conditions of increased metabolic demand. Sci. Rep. *11*, 7717. https://doi.org/10.1038/s41598-021-87292-x.

78. Sandovici, I., Georgopoulou, A., Pérez-García, V., Hufnagel, A., López-Tello, J., Lam, B.Y.H., Schiefer, S.N., Gaudreau, C., Santos, F., Hoelle, K., et al. (2022). The imprinted Igf2-Igf2r axis is critical for matching placental microvasculature expansion to fetal growth. Dev. Cell *57*, 63–79.e8. https://doi.org/10.1016/j.devcel.2021.12.005.

79. Weninger, W.J., Geyer, S.H., Martineau, A., Galli, A., Adams, D.J., Wilson, R., and Mohun, T.J. (2014). Phenotyping structural abnormalities in mouse embryos using high-resolution episcopic microscopy. Dis. Model. Mech. *7*, 1143–1152. https://doi.org/10.1242/dmm.016337.

80. Mohun, T.J., and Weninger, W.J. (2012). Generation of volume data by episcopic three-dimensional imaging of embryos. Cold Spring Harb. Protoc. *2012*, 681–682. https://doi.org/10.1101/pdb.prot069591.

81. Napso, T., Zhao, X., Lligoña, M.I., Sandovici, I., Kay, R.G., George, A.L., Gribble, F.M., Reimann, F., Meek, C.L., Hamilton, R.S., and Sferruzzi-Perri, A.N. (2021). Placental secretome characterization identifies candidates for pregnancy complications. Commun. Biol. *4*, 701. https://doi.org/10.1038/s42003-021-02214-x.

## STAR★METHODS

### KEY RESOURCES TABLE

| REAGENT or RESOURCE | SOURCE | IDENTIFIER |
|---|---|---|
| Antibodies | | |
| Rabbit anti-phospho-AKT (Ser 473) | Cell Signaling Technology | RRID:AB_329825 |
| Polyclonal Goat anti-Mouse IGF-II | R&D Systems | RRID:AB_2122526 |
| Polyclonal Goat anti-Mouse IGF-I | R&D Systems | RRID:AB_2248752 |
| Rabbit polyclonal anti-SOD1 | Abcam | ab183881 |
| Goat anti-Rabbit IgG (HRP) | Abcam | RRID:AB_955447 |
| Rabbit anti-Goat IgG (HRP) | ThermoFisher Scientific | RRID:AB_228390 |
| Recombinant Anti-Argonaute-2 antibody | Abcam | RRID:AB_2713978 |
| Chemicals, peptides, and recombinant proteins | | |
| Human LR3-IGF1 | Preprotech | 100-11R3 |
| Protease inhibitors, set III, Calbiochem | Merck | 535140 |
| Laemmli buffer | Merck | S3401 |
| Laemmli lysis | Merck | 38733 |
| RIPA lysis | Merck | R0278 |
| Immobilon Forte | Merck | WBLUF |
| Coomasie-250 staining | Merck | 1154440025 |
| Critical commercial assays | | |
| In-Fusion HD Cloning Plus kit | Takara Bio | 638909 |
| RNeasy Plus Mini Kit | Qiagen | 74134 |
| mirVana kit | ThermoFisher Scientific | AM1560 |
| miRNeasy Mini kit | Qiagen | 217004 |
| RNase-Free DNase | ThermoFisher Scientific | EN0521 |
| RNA 6000 Pico kit | Agilent | 5067–1513 |
| RNA 6000 Nano kit | Agilent | 5067–1511 |
| DNA 12000 Kit | Agilent | 5067–1508 |
| TruSeq Stranded mRNA HT Sample Prep Kit | Illumina | 20040532 |
| TruSeq Stranded mRNA LT Sample Prep Kit | Illumina | RS-122-2101 |
| TruSeq Small RNA Library Preparation Kit | Illumina | RS-200-0012 |
| RevertAid RT Reverse Transcription Kit | ThermoFisher Scientific | K1622 |
| TaqMan MicroRNA Reverse Transcription Kit | ThermoFisher Scientific | 4366596 |
| SYBR Green JumpStart Taq Ready Mix | Sigma Aldrich (Merck) | S4438 |
| TaqPath ProAmp Master Mix | ThermoFisher Scientific | A30866 |
| TMTsixplex Isobaric Mass Tagging Kit | ThermoFisher Scientific | 90064 |
| BCA assay | ThermoFisher Scientific | 23221 |
| IGF-1 Quantikine ELISA kit | Biotechne | MG100 |
| Mouse/rat growth hormone ELISA kit | Millipore | EZRMGH-45K |
| Mouse IGF-II DuoSet ELISA kit | R&D Systems | DY792 |
| Mouse/Rat Insulin Assay Kit | Mercodia | 10-1247-01 |
| Roche's Free Fatty Acid Kit | Sigma Aldrich (Merck) | 11383175001 |
| QuickChange Site-Directed Mutagenesis kit | Agilent Technologies | 200518 |
| Dual-Luciferase Reporter Assay System | Promega | E1910 |
| Deposited data | | |
| mRNA-seq obtained in E18.5 livers of the *Mir483*$^{KO}$ mouse model | This paper | GEO: GSE256302 |

(*Continued on next page*)

CellPress
OPEN ACCESS

***Continued***

| REAGENT or RESOURCE | SOURCE | IDENTIFIER |
|---|---|---|
| mRNA-seq obtained in E10.5 embryos of the *Mir483*$^{5C}$ mouse model | This paper | GEO: GSE256306 |
| mRNA-seq obtained in livers of 15-week-old *Mir483*$^{KO}$ mutants and littermate controls | This paper | GEO: GSE256304 |
| miR-seq obtained in E18.5 livers of the *Mir483*$^{KO}$ mouse model | This paper | GEO: GSE256305 |
| TMT mass spectrometry data obtained in E10.5 embryos of the *Mir483*$^{5C}$ mouse model | This paper | PXD051516 |
| **Experimental models: Cell lines** | | |
| E14 129ola male ES cells | Hooper et al.[63] | RRID:CVCL_C320 |
| Targeted JM8.F6 ES cells, International Knockout Mouse Consortium Project Design ID: 49935 | Prosser et al.[64] | RRID:CVCL_J961 |
| E14Tg2A.4 ES cells | Smith and Hooper[65] | RRID:CVCL_Y481 |
| **Experimental models: Organisms/strains** | | |
| Mouse *Igf2*$^{\Delta(P1\text{-}P3)}$ | This paper | N/A |
| Mouse *H19*$^{\Delta 13}$ | Leighton et al.[30] | The Jackson Laboratory (Stock #036471) |
| Mouse *Igf2*$^{LacZ}$ | Murrell et al. 2001[35] | N/A |
| Mouse CMV$^{Cre}$ | Schwenk et al.[66] | The Jackson Laboratory (Stock #006054) |
| Mouse *Igf2*$^{fl/fl}$ | Hammerle et al.[34] | N/A |
| Mouse *Mir483*$^{KO}$ | This paper | N/A |
| Mouse *Mir483*$^{5C}$ | This paper | N/A |
| Mouse *iTg*$^{Mir483}$ | This paper | N/A |
| **Oligonucleotides** | | |
| Primers used for genotyping, see Table S7 | This paper | Sigma Aldrich (Merck) |
| TaqMan assays used for RT-qPCR, see Table S7 | This paper | Thermo Fisher Scientific |
| Primers used for RT-qPCR, see Table S7 | This paper | Sigma Aldrich (Merck) |
| **Recombinant DNA** | | |
| pCAG-Cre plasmid | Matsuda and Cepko[67] | RRID:Addgene_13775 |
| pMA_F3NeoLoxPPolyFRT vector | Prosser et al.[64] | N/A |
| pPGKFLPobpA plasmid | Raymond and Soriano[68] | RRID:Addgene_13793 |
| pTET-BigT plasmid | Mao et al.[69] | N/A |
| pROSA26PAS plasmid | Mao et al.[69] | N/A |
| pGL3-Basic | Promega (#E1751) | RRID:Addgene_40342 |
| **Software and algorithms** | | |
| DGE analysis of the effect of Mir-483 on mouse livers | This paper | https://doi.org/10.5281/zenodo.13367806 |
| Cumulative analyses of Mir-483 targets | This paper | https://doi.org/10.5281/zenodo.13369650 |
| Clustal Omega | https://www.ebi.ac.uk/Tools/msa/clustalo/ | RRID:SCR_001591 |
| HALO | Indica Labs | RRID:SCR_018350 |
| MASCOT | Matrix Science | RRID:SCR_014322 |
| Amira 5.4 software | Visage Imaging | RRID:SCR_007353 |
| GraphPad Prism 9 software | GraphPad | RRID:SCR_000306 |
| Image Lab Software | Bio-Rad | RRID:SCR_014210 |
| **Other** | | |
| Lipofectamine RNAiMAX Transfection Reagent | ThermoFisher Scientific | 13778100 |
| Transfection efficiency control | Applied Biosystems | T1003 |
| GloMax Discover Microplate Reader | Promega | GM3000 |

(*Continued on next page*)

*Continued*

| REAGENT or RESOURCE | SOURCE | IDENTIFIER |
|---|---|---|
| Osmotic minipumps | Alzet | 1004 |
| Nitrocellulose western blotting membrane | Merck | GE10600114 |
| PVDF western blotting membrane | Merck | 1154440025 |

## EXPERIMENTAL MODEL AND STUDY PARTICIPANT DETAILS

### Mice

This study was carried out in compliance with the ARRIVE guidelines. The research has been regulated under the Animals (Scientific Procedures) Act 1986 Amendment Regulations 2012 following ethical review and approval by the University of Cambridge Animal Welfare and Ethical Review Body (AWERB). All mouse experiments were approved and performed under PPL No. 70/7594 (study plan 7594/6/15), PPL No. 80/2347 (study plan 2347/2) and PPL No. PC6CEFE59 (study plan IS_AF_001_BF81).

### Generation of the *Igf2*$^{\Delta(P1\text{-}P3)}$ mouse model

The *Igf2* gene targeting vector carried a LoxP site and a FRT-flanked neomycin resistance cassette (Neo) inserted 5' of promoter P1, and a LoxP site inserted 3' of promoter P3 (Figure S3A). Details of the cloning procedures are available upon request. In brief, we used a 4.0-kb *Eco*RV-*Bci*VI genomic fragment as the 5' region of homology (5'-ROH), a 5.8-kb *Bci*VI-*Pci*I genomic fragment that includes the P1-P3 promoters as internal ROH, and a 3.9-kb *Pci*I-*Nde*I genomic fragment (intron 3 to exon 6 of *Igf2*) as 3'-ROH. The targeting vector was linearized at a unique *Sca*I site located at the 5' end of 5'-ROH, and 50 μg linearized vector were electroporated into passage 9, E14 129ola male ES cells, at 250V and 950 μF. Transfected cells were plated onto 10 gelatinized 100-mm dishes pre-seeded with fibroblast feeder cells. After 24 h in nonselective medium, cells were incubated for 8 days with G418 medium (200 μg/μl) to select for neomycin resistance. Resistant clones were picked at day 9 and expanded into 96-well plates pre-seeded with fibroblast feeder cells. We screened 384 G418-resistant clones by Southern blotting analysis of genomic DNA (gDNA) digested with *Spe*I and hybridized the blots with a unique 635 bp 5' probe (located external to 5'-ROH and obtained by PCR amplification using primers 5'Pr-F: 5'-CCTGCATAGACGCCTTCCTG-3' and 5'Pr-R: 5'-GACCCTAACTCTCCCAAGTCCC-3') (Figure S3A). Two correctly targeted clones at the 5' end were then verified by Southern blot (*Eco*RI digested DNA) using a 733 bp 3' probe (located external to 3'-ROH and obtained by PCR amplification using primers 3'Pr-F: 5'-GCCCAAGTAACCTGACCCCT-3' and 3'Pr-R: 5'-CGAG CACCTTCCTAACACCTG-3') and an additional check for multiple integrations elsewhere in the genome using a 628 bp internal probe (located in *Igf2* promoter P1 and obtained by PCR amplification with primers Int-F: 5'-CCACCACATTTAGACAGCATT-3' and Int-R: 5'-ACCGTAGGAGAAGTGACGAG-3'). Two clones with a single integration site and correctly targeted 5' and 3' LoxP sites were thus identified (Figures S3B, S3C and S3D), with the loxP sequences further verified by Sanger sequencing. The Neo cassette was excised by transiently transfecting the two ES cell clones with FLPe recombinase, followed by two rounds of subcloning. Four correctly excised clones that carried a single FRT site and lack the Neo cassette were identified by PCR screening (288 ES subclones) using primers F: 5'-ATGTCTCCAATCCTTGAACACTG-3' and R1: 5'-GCAGTGGGAGAAATCAGAACC-3' (Figure S3E). Two independent ES clones were then microinjected into C57BL/6J blastocysts and transferred into (C57BL/6J X CBA/Ca) F1 pseudo-pregnant females to generate chimeric mice. Four chimeras were born, two males and two females and germline transmission was achieved only through the females. Germline transmitting mice were backcrossed into the C57BL/6J genetic background for more than 10 generations before being used as experimental animals, and genotyping was performed by PCR using primers listed in Table S7 (Figure S3F). Efficient deletion of the floxed *Igf2* P1-P3 region upon paternal transmission of the targeted allele was verified by PCR (Figure S3G) and by Northern blotting (Figure S3H).

### Generation of a *Mir483* specific knockout mouse

Targeted JM8.F6 ES cells (from the C57BL/6N mouse strain) (International Knockout Mouse Consortium Project Design ID: 49935) were provided by Dr. Haydn Prosser[64]. Confirmation of targeting was performed by Southern blotting (Figure S4). The 5' probe (587 bp, generated by PCR amplification using primers 5'arm_F2: 5'-GGCTTACTGTGGGTCATCGT-3' and 5'arm_R2: 5'-CTGGA CACTGGACCTGGTTT-3') was hybridised to *Eco*RV-digested DNA to give expected bands of 12,984 bp (targeted locus, *PGKPuroΔtk* or Mut allele) and 18,532 bp (wild-type locus, WT). The 3' probe (685 bp, generated by PCR amplification using primers 3'arm_F: 5'-ATGTGTGACCAGGCTGCTAGTTC-3' 3'arm_R: 5'-GTGTTGATGGCTCTAGCTGGTGT-3') was hybridized to *Xba*I-digested DNA to give expected bands of 3,562 bp (Mut) and 10,447 bp (WT). A single insertion of the targeted vector was confirmed using the Puro probe (537 bp, generated by PCR amplification using primers Puro_F: 5'-GGTCACCGAGCTGCAAGAAC-3' and Puro_R: 5'-AGTTGCGTGGTGGTGGTTTT-3') hybridized to *Eco*RV-digested DNA, which gives the band of 12,984 bp (Mut), or to *Xba*I-digested DNA, which gives the expected band of 9,104 bp (Mut).

ES cells were cultured using standard conditions as recommended by the International Knockout Mouse Consortium. Selection marker was excised by transient *in vitro* transfection with pCAG-Cre (a gift from Dr. Connie Cepko – Addgene plasmid 13775[67])

encoding a Cre recombinase and ES cells were then genotyped by PCR using primers F1: 5'-TACCTGCCTGTGAACTGCTCTG-3', R1: 5'-ATCTGGTGCCTCCTGTCTGGTA-3' and R2: 5'-CTCTGAGCCCAGAAAGCGAAG-3' (expected product sizes: *PGKPuroΔTK* allele – 560 bp, WT allele – 440 bp, *Mir483*$^{KO}$ allele – 457 bp). *Mir483*$^{KO}$ ES cells from two independent clones (F4 and E12) were injected into C57BL/6J-Tyrc-2J (albino) blastocysts. Chimeric mice (identified by their coat colour) were then mated with albino B6 mice and germline transmission was validated by the appearance of black offspring. Subsequently, the colony was maintained by crossing with C57BL/6J wild-types. Mice were genotyped by PCR using primers F1 and R1 (Figure S4).

## Generation of *Mir483*$^{5C}$ mice with five copies of *Mir483* at the endogenous locus

To generate the plasmid for recombinase-mediated cassette exchange (RMCE), we performed the following steps (Figure S7A). (1) First, one copy of *Mir483* (each 373-bp unit contains the 73-bp *pre-miR-483* sequence and 150-bp flanking intronic sequences on each side, selected to include putative target sequences bound by the DROSHA/DGCR8 complex that processes pri-miRs into pre-miRs[70,71]) was amplified using primers 102_F: 5'-ccccccctcgaggtcgacggtatcgatTCTTCACTTCTGCCTACCTGCCTG-3' and 107_R: 5'-CGGGCTGCAGGAATTCagtggtttggaaaacagggaggag-3'. The PCR product thus obtained was cloned using the In-Fusion HD Cloning Plus kit (Takara Bio – 638909) into the pMA_F3NeoLoxPPolyFRT vector provided by Dr. H. Prosser[64], linearized with *Cla*I and *Eco*RI. The resulting one-copy plasmid was digested with *Eco*RI and *Bam*HI and two further copies of *Mir483* (generated by PCR using primers 103: 5'-AAGCTTAGTGGTTTGGAAAACAGGGAGGAG-3' plus 108: 5'-CCAAACCACTGAATTTCTTCACTTCT GCCTACCTGCCTG-3' and 104: 5'-CAAACCACTAAGCTTTCTTCACTTCTGCCTACCTGCCTG-3' plus 109: 5'-TAGCCCGGGCG GATCCAGTGGTTTGGAAAACAGGGAGGAG-3') were cloned in, using the In-Fusion HD Cloning Plus kit. The resulting three-copy construct was further digested with *Bam*HI and two additional copies of *Mir483* (generated by PCR using primers 112: 5'-CCAAAC CACTGGATCCTCTTCACTTCTGCCTACCTGCCTG-3' plus 105: 5'-AGGCAGAAGTGAAGAAGTGGTTTGGAAAACAGGGAGGAG-3' and 106: 5'-GTTTTCCAAACCACTTCTTCACTTCTGCCTACCTGCCTG-3' plus 109: 5'-TAGCCCGGGCGGATCCAGTGGTTTG GAAAACAGGGAGGAG-3') were additionally cloned-in to produce the five-copy plasmid. (2) To perform the RMCE and screening, the five-copy plasmid obtained above was mixed with the pPGKFLPobpA plasmid (a kind gift from Dr. Philippe Soriano – Addgene plasmid 13793[68]), encoding FLPo recombinase and the mix was co-electroporated into ES cell clones which had confirmed replacement of *Mir483* with PGKPuroΔtk allele (Figure S7A). Single-cell-derived colonies were picked and screened by PCR using primers F2: 5'-GTGCCACTCCCACTGTCCTT-3' and R1: 5'-ATCTGGTGCCTCCTGTCTGGTA-3', generation of a 2,430 bp product indicating that RMCE had occurred and five copies of *Mir483* were present (Figure S7B). The identity of the PCR product was further confirmed by digestion with *Bam*HI, *Hind*III or *Cla*I (Figure S7C). (3) The intermediate Neo cassette was deleted *in vitro* by transfection with pCAG-Cre (a gift from Dr. Connie Cepko – Addgene plasmid 13775[67]) encoding a *Cre*-recombinase. ES cells were genotyped using primers 142: 5'-CACGCTTCAGTTTGTCTGTTCG-3', 144: 5'-CGTGCTACTTCCATTTGTCACG-3' and 145: 5'-CTGGAGTGGTTTG GAAAACAGG-3', which generate products of 1,014 bp (Neo$^+$ allele) and 925 bp (WT allele), and also using primers 142, 145 and 143: 5'-AAGAATCGATACCGTCGACCTC-3', which generate products of 740 bp (Neo$^-$ allele) and 925 bp (WT allele). This latter set of three primers was also used for genotyping *Mir483*$^{5C}$ mice (Figure S7D).

## Generation of *iTg*$^{Mir483}$ mice with an additional copy of *Mir483* inserted at the *Rosa26* locus

To generate the targeting vector containing one-copy of *Mir483* with Tet regulation (*Mir483*$^{1C}$), *Mir483* was amplified from the one-copy plasmid made for RMCE, using primers 135: 5'-TGCAGCCCAAGCTAGCCCCTCGAGGTCGACGGTATCGAT-3' and 136: 5'-GCGGGGGCCCCTCGAGCTCCACCGCGGTGGCGGCCGCTC-3' and the resulting 485 bp product was cloned between *Nhe*I and *Xho*I restriction sites of the pTET-BigT plasmid (a kind gift from Dr. Andrew P. McMahon[69]). To enable ES cell targeting at the *Rosa26* locus, the pTET-BigT-*Mir483*$^{1C}$ construct was digested with *Pac*I and *Asc*I and subcloned into pROSA26PAS (a kind gift from Dr. Andrew P. McMahon[69]). pROSA26PAS-*Mir483*$^{1C}$ was linearised with *Afe*I and electroporated into BayGenomics E14Tg2A.4 ES cells and JM8.F6 ES cells.

ES cell clones were screened by PCR. Homologous recombination of the 5' arm was assayed using primers 146: 5'-CGCCTAAA GAAGAGGCTGTG-3' and 148: 5'-GAAAGACCGCGAAGAGTTTG-3' (expected product size of 1,316 bp). Homologous recombination of the 3' arm was assayed using primers 152: 5'-GGGAGGATTGGGAAGACAAT-3' and 153: 5'-CGAAGACCTGTTGCTGCTCA -3' (expected product size of 4,779 bp) (Figure S8B).

Targeted ES clones and the subsequent transgenic mice were also genotyped using primers 147, 148, 156, which generate 326 bp product from *Tg* (Neo$^+$) allele and 436 bp from the WT allele (Table S7). *In vivo Cre*-mediated deletion of the Neo cassette was determined using primers 151 and 143, which generate a 266 bp product from the deleted allele (*iTg*$^{Mir483}$), or a 2,931 bp product if deletion does not occur (Table S7). Presence of *Cre*-recombinase was confirmed using primers Cre-F and Cre-R and Ctrl-F and Ctrl-R, which generate a 390 bp product from the *Cre* transgene and a control 254 bp product (Figure S8D).

## Additional mouse strains and mouse husbandry

*H19*$^{\Delta 13}$ mutant mice[30], *Igf2*$^{LacZ}$ mice[35] and *CMV*-Cre mice[66] were generated previously and were obtained from the Babraham Institute, Cambridge. The *Igf2*$^{fl/fl}$ mice were generated in our laboratory, as previously described[34]. The *CMV*-Cre recombinase is expressed soon after fertilization and allows ubiquitous deletion of floxed alleles in all tissues, including the germline[66]. C57BL/6J mice used as wild-type (WT) controls were purchased from Charles River (Strain Code: 632).

All mouse work was performed unto a C57BL/6J genetic background. Mice were maintained and mated under pathogen-free conditions at the University of Cambridge Phenomics Unit (West Forvie). They were fed a standard chow diet with 9% of kcal from fat (SDS, Essex, UK), and housed with a 12-h light/dark cycle in a temperature-controlled room (22°C). Food and water were available *ad libitum*, except for periods of fasting when food was withdrawn. For timed mating, the day of detection of a vaginal plug was noted as embryonic day 0.5 (E0.5) and the day of birth was noted as post-natal day 0 (P0). Mice were weaned at 3 weeks of age and ear notches were used for visual identification and genotyping, which was performed using standard PCR, with primers listed in Table S7, followed by separation of PCR amplicons by agarose gel electrophoresis. With the exception of RNA-seq and miR-Seq analyses and IGF1 infusion via minipumps that were performed in only one sex, as indicated in the methods and figure legends, all other analyses included both sexes.

## METHOD DETAILS

### Nomenclature

Throughout the paper, we are using the current nomenclature for microRNAs, i.e. *Mir483* and *MIR483* when referring to the mouse and human microRNA gene, respectively, *miR-483-3p* and *miR-483-5p* when alluding to the mature miR, and *mir-483* when specifying the stem-loop[14].

### Southern blotting

Southern blotting analyses of genomic DNA extracted from ES clones was performed as previously described[34]. Briefly, genomic DNA were digested with appropriate restriction enzymes, then DNA fragments were separated by electrophoresis on 0.8% agarose gels in 1×TBE buffer, alkaline blotted onto Hybond N+ membranes (Amersham), and UV cross-linked (Stratalinker, Stratagene). Probes were obtained by PCR as described above and radiolabelled (α-32P-CTP). After hybridisation and washing, the membranes were exposed overnight to MS film (Kodak).

### Northern blotting

Northern blotting analysis of *Igf2* expression in E18.5 placenta and liver samples was performed as previously described[34]. Briefly, total RNA (10 μg) was separated in low-percentage formaldehyde-treated agarose gels, blotted onto Nytran-plus membrane (Schleicher and Schuell), and UV cross-linked (Stratalinker, Stratagene). The RNA blots were hybridized with radiolabelled (α-32P-UTP) *Igf2* and *Gapdh* (internal control) cDNA probes. After hybridization and washing, transcript levels were quantified by PhosphorImager analysis (Molecular analyst software, Biorad).

### Sequence alignment

Sequences of eutherian mammals and marsupials corresponding to *Mir483*, its promoter and target sequences at the *Igf2* 5'UTR and *Igf1* 3'UTR were retrieved from NCBI (National Center for Biotechnology Information). These sequences were then aligned using Clustal Omega (https://www.ebi.ac.uk/Tools/msa/clustalo/), using ClustalW as output format.

### RNA extraction and RT-qPCR

Total RNA was extracted from tissues or cells using RNeasy Plus Mini Kits (Qiagen – 74134). Small RNAs were extracted using mirVana kits (ThermoFisher Scientific – AM1560) or miRNeasy Mini kits (Qiagen – 217004). Total RNA was treated with an RNase-Free DNase Set (ThermoFisher Scientific – EN0521). RNA concentrations were measured by NanoDrop (Thermo Scientific) and quality was assessed in 1.2% agarose gels, or using the RNA 6000 Pico or Nano Kits (Agilent – 5067-1513 and 5067-1511) and an Agilent 2100 Bioanalyzer. Total RNA (200 ng) was reverse transcribed into cDNA using the RevertAid RT Reverse Transcription Kit (ThermoFisher Scientific – K1622). For microRNA, reverse transcription was performed using the TaqMan MicroRNA Reverse Transcription Kit (ThermoFisher Scientific – 4366596) from 4.8 ng total small RNA.

RT-qPCR was performed with the SYBR Green JumpStart Taq Ready Mix (Sigma – S4438) and custom-made primers (Table S7), or with TaqPath ProAmp Master Mix (ThermoFisher Scientific – A30866) and TaqMan probes (Table S7) in an ABI Prism 7900 system or QuantStudio6 Real-time PCR machine (Applied Biosystems). Gene expression normalisation was performed against combinations of the housekeeping genes: *Ppia* (peptidylpropyl isomerase A or cyclophilin-A), *Gapdh* (glyceraldehyde 3-phosphate dehydrogenase), *Pmm1* (phosphomannomutase 1), *Hprt* (hypoxanthine phosphoribosyltransferase), *Actb* (actin beta), *Tbp* (TATA box binding protein) and *Sdha* (succinate dehydrogenase complex flavoprotein subunit A), as appropriate. For small RNAs, expression levels were normalized against *Snord70/snoRNA234* and/or *Snord68/snoRNA202*, used as internal controls. Relative levels of expression were calculated using the $2^{-\Delta\Delta Ct}$ method[72].

### RNA sequencing and bioinformatic analyses

For RNA-seq analyses, total RNA was isolated using miRNeasy Mini Kits (Qiagen – 217004), according to the manufacturer's instructions. RNA was isolated from livers of E18.5 *Mir483*$^{Pat\text{-}KO}$ (n=5) and WT littermate (n=5) female fetuses; lysates of whole E10.5 male *Mir483*$^{5C}$ (n=3 pools of 2 embryos each) and male WT littermates (n=3 pools of 2 embryos each); livers of 15-week-old *iTg*$^{Mir483}$ (n=6) and control (n=6) female mice. Total RNA was quality checked (RIN score > 8.5) using the Agilent Bioanalyzer 2100 system, using the

Agilent RNA 6000 Nano Kit. For the *Mir483*$^{Pat\text{-}KO}$ and *iTg*$^{Mir483}$ models 500 ng total RNA/sample was used to construct barcoded sequencing libraries with the TruSeq Stranded mRNA HT Sample Prep Kit (Illumina – 20040532) following the supplier's instruction. For the *Mir483*$^{5C}$ model, 800 ng total RNA/sample was used to construct barcoded sequencing libraries with the TruSeq Stranded mRNA LT Sample Prep Kit (Illumina – RS-122-2101) following the supplier's instruction. All the libraries were validated using the DNA 12000 Kit (Agilent – 5067-1508) and Agilent Bioanalyzer 2100, then multiplexed and sequenced. For the *Mir483*$^{Pat\text{-}KO}$ and *iTg*$^{Mir483}$ models, sequencing was performed on an Illumina NovaSeq6000 at PE50 and for the *Mir483*$^{5C}$ model, sequencing was achieved on an Illumina HiSeq at SE40, both platforms hosted at CRUK Cambridge Institute Genomics Core.

For miR-seq analysis in livers of E18.5 *Mir483*$^{Pat\text{-}KO}$ (n=7) and WT littermate (n=5) female fetuses, 1 μg total RNA isolated using miRNeasy Mini Kit was used to construct barcoded sequencing libraries with Illumina's TruSeq Small RNA Library Preparation Kit (Illumina – RS-200-0012), according to manufacturer's protocol. All the libraries were validated using the DNA 12000 Kit (Agilent – 5067-1508) and Agilent Bioanalyzer 2100, then multiplexed and sequenced. Sequencing was performed on an Illumina NovaSeq6000 at PE50 hosted at CRUK Cambridge Institute Genomics Core.

For mRNA sequencing analysis, STAR 2.5.0a was used to align sequence reads to the mouse genome (built GRCm38, Ensembl Version 100) and to determine gene-level counts. For miRNA analysis, the alignment and counts were performed using miRge (3.0). The count tables were then imported into edgeR (3.42.4) and limma (3.56.2) for differential gene expression using quasi-likelihood GLM F-test.

Functional analyses were performed using DAVID[73]. Enriched gene ontology (GO) terms with FDR < 5% were considered significant. These terms were then clustered semantically using REViGO[74], which removes redundancy, and ordered according to the $\log_{10}$ P values. For cumulative fraction analyses, predicted targets of miRNAs were determined by adopting a cut-off of -0.3 to the cumulative weighted context++ score of TargetScan 8.0 datasets[75]. Cumulative distributions of the fold changes of mRNA levels of predicted miRNA targets and non-targets were calculated using R v.4.3.2. To determine if differences in distributions were significant, two-sided Kolmogorov-Smirnov tests were employed.

## Food intake

Food intake was measured in the *iTg*$^{Mir483}$ model between W3 (week 3) and W4. Briefly, two mice of same sex and genotype were placed in a new cage and the food pellets were weighted at 4 pm at the start and the end of the seven-day interval. The average food consumed (g/day/mouse) was calculated by dividing the food consumed per cage in a week by 14.

## Body composition

For the *Mir483*$^{KO}$ model, body composition was analysed by DEXA scanning (Lunar PIXImus densitometer, General Electric) immediately after killing by cervical dislocation between 8 am and 10 am. For fat mass and lean mass, values were expressed as a proportion of total body weight, and the bone mass density (BMD) was calculated related to the body length (naso-anal distance) and presented as g/cm$^2$. For the *iTg*$^{Mir483}$ model, body composition analysis was performed at W4 and W8 on live and conscious mice, using time-domain nuclear magnetic resonance spectroscopy (TD-NMR) with the Minispec Live Mice Analyser (Bruker Minispec Live Mice Analyser LF50) that measures total body fat mass and lean mass[76]. For *iTg*$^{Mir483}$ mice that underwent surgery (minipump insertion), the TD-NMR at W8 was performed post-mortem, immediately after the removal of the empty minipump.

## Histology and stereology analyses

Immediately after dissection, tissues (gonadal fat, *vastus lateralis* skeletal muscle and liver) were fixed in 10% buffered formalin for 48 hours, then were dehydrated and embedded in paraffin. Paraffin blocks were cut at 5 μm thickness, sections were then deparaffinised, rehydrated, stained (using a standard haematoxylin-eosin staining protocol) and mounted with coverslips. For all stereological analyses, in order to obtain accurate morphometric estimations, at least two sections/block, spaced at 200 μm, were used. The stained slides were imaged using the Zeiss Axioscan Z1 Slidescanner (Carl Zeiss). Whole-slide scans of stained sections were analysed using HALO or HALO AI (Indica Labs) to measure the area of individual adipocytes, skeletal muscle fibres or the percentage occupied by vacuoles in the liver.

## Glucose tolerance tests

Glucose tolerance tests were performed as previously described[77]. Briefly, for the *Mir483*$^{KO}$ model, glucose was administered at 1 mg/g body weight by intra-peritoneal injection (ipGTT) after 16 h fasting (5 pm to 9 am the following day) at the age of 12 weeks. For the *iTg*$^{Mir483}$ model, glucose was administered at 2 mg/g body weight by oral gavage (OGTT) after 6 h fasting (8 am – 2 pm) at the age of 13 weeks. The areas under the curve (AUCs) following ipGTTs and OGTTs were calculated by the trapezoidal rule, after normalization to basal glucose levels.

## Tandem Mass Tag (TMT) analysis

This analysis was performed using the TMTsixplex Isobaric Mass Tagging Kit (ThermoFisher Scientific – 90064) that allows multiplexing up to six samples. Three WT and three 5C embryos at E10.5 were first suspended in RIPA buffer (ThermoFisher Scientific – 89901) and dissociated using Dounce homogenizers. Protein concentrations were determined using a BCA assay (ThermoFisher Scientific – 23221). Once quantified, 100 μg protein per condition were transferred, to a final volume of 100 μL with

100 mM TEAB (triethylammonium bicarbonate buffer). Then, 5 μL of the 200 mM TCEP (tris(2-carboxyethyl)phosphine) was added to every sample, which were then incubated at 55°C for 1 hour. The samples were incubated for additional 30 minutes and protected from light once 5 μL of the 375 mM iodoacetamide were mixed in. The proteins were then precipitated with excess pre-chilled (-20°C) acetone and incubated overnight at -20°C. Samples were centrifuged (8000 × *g* for 10 minutes at 4°C), the supernatant removed and the pellet air-dried. Subsequently, the pellet was re-suspended with 100 μL of 100 mM TEAB. In addition, 2.5 μL of 1 μg/μLtrypsin solution (*i.e.* 2.5 μg) were added to each protein sample, followed by overnight digestion at 37°C. 41 μL of anhydrous acetonitrile were added to each of the mass tag (with reporter ions from m/z = 126.1 to 131.1) and each merged with one protein sample, in no particular order. The reaction was incubated for 1 hour at room temperature and quenched with 8 μL of 5% hydroxylamine for further 15 minutes. Finally, the samples were pooled at equal concentrations and stored at -80°C until mass spectrometry analysis.

Digested, labelled sample was fractionated using off-line tip-based SAX fractionation. Briefly, 30–50 μg tryptic peptide was loaded at pH 11 on a tip-based anion exchanger constructed using six layers of Empore anion exchange disk (3M, Bracknell, UK). The column was equilibrated and fractions eluted using Britton & Robinson buffer (20 mM acetic acid, 20 mM phosphoric acid, 20 mM boric acid titrated with NaOH to the desired pH). Fractions were eluted subsequently with buffer solutions of pH 11, 8, 6, 5, 4, and 3 onto StageTips containing three layers of C18 membrane. All data was acquired on a Q Exactive coupled to an RSLC3000 nanoLC via an EASYspray source. MSMS was acquired from 400 to 1650 m/z at 70,000 fwhm. Peptides were fragmented at 32 NCE with fragments scanned at 35,000 fwhm with a fixed first mass of 100 m/z. RSLC3000 was operated using solvent A (0.1% formic acid) and solvent B (80% MeCN, 0.1% formic acid) with peptides fractionated using a 50cm EASYspray column at 250nl/min flow rate.

Data was processed in Maxquant v1.4.1.2. using a Uniprot Mus musculus database (downloaded 14/8/2012). Carbamidomethyl (C) was set as a fixed modification and oxidation (M), deamidation (NQ) and acetylation (protein N-terminus) were set as variable modifications.

### Protein extraction and western blotting

Protein was extracted in lysis buffer (50 mmol/l HEPES [pH 8], 150 mmol/l NaCl, 1% (wt/vol.) Triton X-100, 1 mmol/l $Na_3VO_4$, 30 mmol/l NaFl, 10 mmol/l $Na_4P_2O_7$, 10 mmol/l EDTA (all Sigma-Aldrich, Merck) with a cocktail of protease inhibitors (set III, Calbiochem, Merck – 535140). Total protein concentration of lysates was determined using a bicinchoninic acid kit (Merck – BCA1) and samples diluted in Laemmli buffer (Merck – S3401). Total protein from tissue extracts or plasma were prepared in Laemmli lysis (Merck – 38733) or RIPA (Merck – R0278) buffers, separated by polyacrylamide gel electrophoresis and then transferred to either a nitrocellulose (Merck – GE10600114) or a PVDF (Merck – 03010040001) western blotting membranes. Membranes were then processed for Western blotting using the antibodies listed in Table S8. Protein bands were visualised using a chemiluminescence substrate (Immobilon Forte, Merck – WBLUF) on the ChemiDoc Imaging system (Bio-Rad). Protein abundance was quantified by band densitometry, using Image Lab 6.1 software (Bio-Rad), and normalised to levels of SOD1 or the total protein transferred, assessed by Coomasie-250 staining (Merck – 1154440025).

### IGF1, IGF2 and GH measurements by ELISA

All measurements were performed at CBAL (Core Biochemical Assay Laboratory, Addenbrooke's hospital). IGF1 was measured using the mouse/rat IGF-1 Quantikine ELISA kit (Biotechne – MG100), which employs the quantitative sandwich enzyme immunoassay technique. Briefly, a monoclonal antibody specific for mouse/rat IGF-1 was pre-coated onto a microplate. Standards, controls and samples were pipetted, in duplicate, into the wells and any IGF-1 present became bound by the immobilized antibody. After washing, an enzyme-linked polyclonal antibody specific for mouse/rat IGF-1 was added to the wells. Following a wash to remove any unbound antibody-enzyme reagent, a substrate solution was added to the wells and colour developed in proportion to the amount of IGF-1 bound in the initial step. The colour development was stopped and the intensity of the colour was measured on the Perkin Elmer Victor3 plate reader. Samples were assayed in duplicate on a 1:500 dilution.

GH was measured using a mouse/rat growth hormone ELISA kit (Millipore – EZRMGH-45K), based on quantitative sandwich enzyme immunoassay technique. Briefly, a polyclonal antibody specific for mouse/rat growth hormone was pre-coated onto a microplate. Standards, controls and samples were pipetted, in duplicate, into the wells and any growth hormone present became bound by the immobilized antibody. After washing, a biotinylated polyclonal antibody specific for mouse/rat growth hormone was added to the wells. After further washing, a streptavidin-horseradish peroxidase conjugate was added to the wells. Following a wash to remove any unbound conjugate, a substrate solution was added to the wells and colour developed in proportion to the amount of growth hormone bound in the initial step. The colour development was stopped and the intensity of the colour was measured at 450nm on the Perkin Elmer Victor3 plate reader. Samples were assayed in duplicate, using 10 μl undiluted plasma.

IGF2 measurements in E13.5 whole-embryo lysates were performed with the Mouse IGF-II DuoSet ELISA kit (R&D Systems – DY792), using an assay adapted for the MesoScale Discovery electrochemiluminescence immunoassay platform (MSD), as previously described[78].

### Plasma insulin and total-pancreas insulin measurements

Blood samples for plasma insulin measurements were collected from the tail vein in heparinised capillary tubes during the OGTT experiments at 0 and 20 min, from W13 $iTg^{Mir483}$ mouse model. The tubes were kept on ice and spun at 4,000 RPM (rotations per minute) for 5 min. Plasma samples were flash-frozen in liquid N2 and stored at –80°C until analysis. For total-pancreas insulin measurements,

the entire pancreases collected from W8 *iTg*$^{Mir-483}$ mouse model were collected into cold acid-ethanol (0.18M hydrochloric acid in 70% (vol/vol) ethanol and flash frozen in liquid N2, then pulverised and sonicated, stored at 4°C overnight before storage at -70°C until analysed. Insulin levels in plasma were measured using ELISA kits (Meso Scale Discovery Mouse/Rat Insulin Assay Kit) at CBAL (Core Biochemical Assay Laboratory, Addenbrooke's hospital). Insulin levels in acid–ethanol supernatants were measured using ELISA kits (Mercodia – 10-1247-01). Total pancreas insulin content (pmol/L) was normalised to the total pancreas wet weight (g), measured at collection.

## Blood biochemistry

Serum cholesterol, triglycerides and free fatty acids concentrations were measured using enzymatic assay kits, as previously described[77]. Briefly, total cholesterol was measured using an enzymatic assay kit (Siemens Healthcare – DF27) that combines activities of cholesterol esterase and cholesterol oxidase. Triglycerides were measured using an enzymatic assay kit (Siemens Healthcare – DF69A) that combines activities of lipoprotein lipase, glycerol kinase and glycerol-3-phosphate oxidase. The assays for total cholesterol and triglycerides were automated on the Siemens Dimension EXL analyser. Free (non-esterified) fatty acids were measured using Roche's Free Fatty Acid Kit (half-micro test) (Sigma Aldrich – 11383175001), which is based on the enzymatic conversion of free fatty acids to acyl CoA by acyl-Co A synthetase.

## High-resolution episcopic microscopy (HREM)

E14.5 fetuses were fixed, dehydrated, infiltrated and embedded as previously described[79]. Embedded fetuses were analysed by HREM, using 3-μm sections, green fluorescent protein filters and a Hamamatsu Orca HR CCD camera to obtain the high-resolution images, as previously shown[80], and datasets were analysed with the Amira 5.4 software (Visage Imaging). For illustration, volume rendering was combined with arbitrary section plane erosion in Amira, to obtain the fetus models shown in Figure 4. Individual aortae, pulmonary arteries and esophagus were also digitally segmented from image stacks in Amira, and used to generate surface-rendered pseudo-coloured 3D organ models, which were superimposed in the appropriate location upon semi-transparent volume rendering of the fetus.

## Ago2 immunoprecipitation

Undifferentiated 3T3-L1 cells were transfected with 100 nM of mouse *miR-483*-3p 2′-O-Methyl antagonist, or a scrambled 2′-O-Methyl RNA sequence (both custom-made, Sigma). The cells were harvested in PBS, and then fixed in the presence of 1% formaldehyde for 1 hour, to establish protein-RNA reversible crosslinks. Cells were lysed and sonicated, then the protein lysate was immunoprecipitated using a specific Ago2 mouse monoclonal antibody (Abcam – ab186733) and bound by A/G agarose beads (Santa Cruz Biotechnology – SC-2003). Beads bound to the antibody were resuspended in water after precipitation and heated at 75°C for 45 min to disrupt the RNA-protein interactions. The RNA was then extracted and purified using Trizol. *Igf1* and *Actb* were quantified by RT-qPCR (primer sequences: *Actb*_F: 5'-CGACAACGGCTCCGGCATGT-3', *Actb*_R 5'-TCACACCCTGGTGCCTAGGGC-3', *Igf1*_F: 5'-AGCATACCTGCCTGGGTGTCCA-3', *Igf1*_R: 5'-TGTGTATCTTTATTGCAGGTGCGGT-3').

## *In vitro* luciferase assays

Luciferase reporter constructs were generated by PCR amplification of approximately 600 bp of *Igf1* 3′UTR encompassing the *miR-483-3p* seed regions. These PCR products were subcloned downstream of the luciferase gene contained in the pGL3-Basic commercial vector (Promega – E1751). Mutation of the highly cross-species conserved *miR-483*-3p seed target sequence from AGGAGUG to AGGAACG (mouse) was performed using the QuickChange Site-Directed Mutagenesis kit (Agilent Technologies – 200518). The HEK-293 cells were used for *miR-483*-3p expression studies, and HepG2 cells for *miR-483*-3p knockdown studies, because of their low and high levels of endogenous *miR-483*-3p expression, respectively. Cells were transfected with 100 ng of reporter construct, 50 ng of pLacZ-Control Vector (a transfection control, Clontech – 631709) and increasing concentrations of mouse *miR-483*-3p mimic (0, 10 and 50 nM in HEK-293) or *miR-483*-3p 2′-O-Methyl antagonist (anti-human; 0, 50 and 100 nM in HepG2) using the Lipofectamine RNAiMAX Transfection Reagent (ThermoFisher Scientific – 13778100). Cells were harvested and the luciferase assay was performed as previously described[21] using the Dual-Luciferase Reporter Assay System (Promega – E1910), with a transfection efficiency control (Applied Biosystems – T1003). Luminescence was detected using the GloMax Discover Microplate Reader (Promega – GM3000).

## Continuous IGF1 administration via minipumps

Human LR3-IGF1 (Preprotech – 100-11R3) was dissolved in 100 mM acetic acid in pH 7.4 sterile PBS, at a concentration of 9 μg/μl. Osmotic minipumps (Alzet – model 1004), which are designed to deliver a constant volume of 0.13 μl/hour, were pre-filled with 110 μl LR3-IGF1 or vehicle under sterile conditions. The concentration was calculated to deliver an average dose of 1.5 μg LR3-IGF1/g body weight/day. Prior to surgery, the minipumps were primed overnight in sterile PBS at 37°C. Surgery was performed on W4 *iTg*$^{Mir483}$ mutant males, a time-point when they were above 10 g body weight. The mini pumps were implanted subcutaneously under general anaesthesia, in the subcutaneous scapular area, below the shoulder blade, just off midline. The mice were given post-operative analgesia for one day, and were monitored daily for one week. Each week, a small blood sample was collected from the tail vein and 5 μl plasma was used for mouse IGF1 measurement by ELISA and 5 μl plasma for human LR3-IGF1 measurements by liquid

chromatography and mass-spectrometry (LC-MS). Plasma LR3-IGF1 concentrations (ng/mL) were calculated by analysis of the human LR3-IGF1-specific tryptic peptide GFYFNKPTGYGSSSR against a standard curve prepared with serial dilutions of the stock LR3-IGF1 solution in mouse plasma (50-1000 ng/mL). Plasma proteins were extracted as previously described[81] and analysed on a Waters M Class nano LC system (Milford, MA, USA), coupled to a Xevo TQ-XS triple quadrupole (Waters, MA, USA). The LR3-IGF1 specific peptide was monitored using the *m/z* transitions 556.8 / 177.0, and peptide peak area ratios were generated against a spiked internal standard with bovine insulin (Sigma – 11070-73-8).

## QUANTIFICATION AND STATISTICAL ANALYSIS

Statistical analyses were performed using GraphPad Prism 9 software. For two groups, statistical analyses were performed using Mann–Whitney tests or un-paired Student's *t*-tests with Welch's correction (depending on the outcome of Shapiro–Wilk tests for normal distribution). Where more than two groups were analysed, we used one-way ANOVA, followed by Tukey's multiple comparisons tests or two-way ANOVA followed by Sidak's corrections for multiple testing, as appropriate. For growth kinetics analyses, we used mixed-effects model (REML) tests. For all tests, $P$ values $< 0.05$ were considered significant.

Cell Reports, Volume *43*

## Supplemental information

# Overexpression of *Igf2*-derived *Mir483* inhibits *Igf1* expression and leads to developmental growth restriction and metabolic dysfunction in mice

**Ionel Sandovici, Denise S. Fernandez-Twinn, Niamh Campbell, Wendy N. Cooper, Yoichi Sekita, Ilona Zvetkova, David Ferland-McCollough, Haydn M. Prosser, Lila M. Oyama, Lucas C. Pantaleão, Danilo Cimadomo, Karina Barbosa de Queiroz, Cecilia S.K. Cheuk, Nicola M. Smith, Richard G. Kay, Robin Antrobus, Katharina Hoelle, Marcella K.L. Ma, Noel H. Smith, Stefan H. Geyer, Lukas F. Reissig, Wolfgang J. Weninger, Kenneth Siddle, Anne E. Willis, Brian Y.H. Lam, Martin Bushell, Susan E. Ozanne, and Miguel Constância**

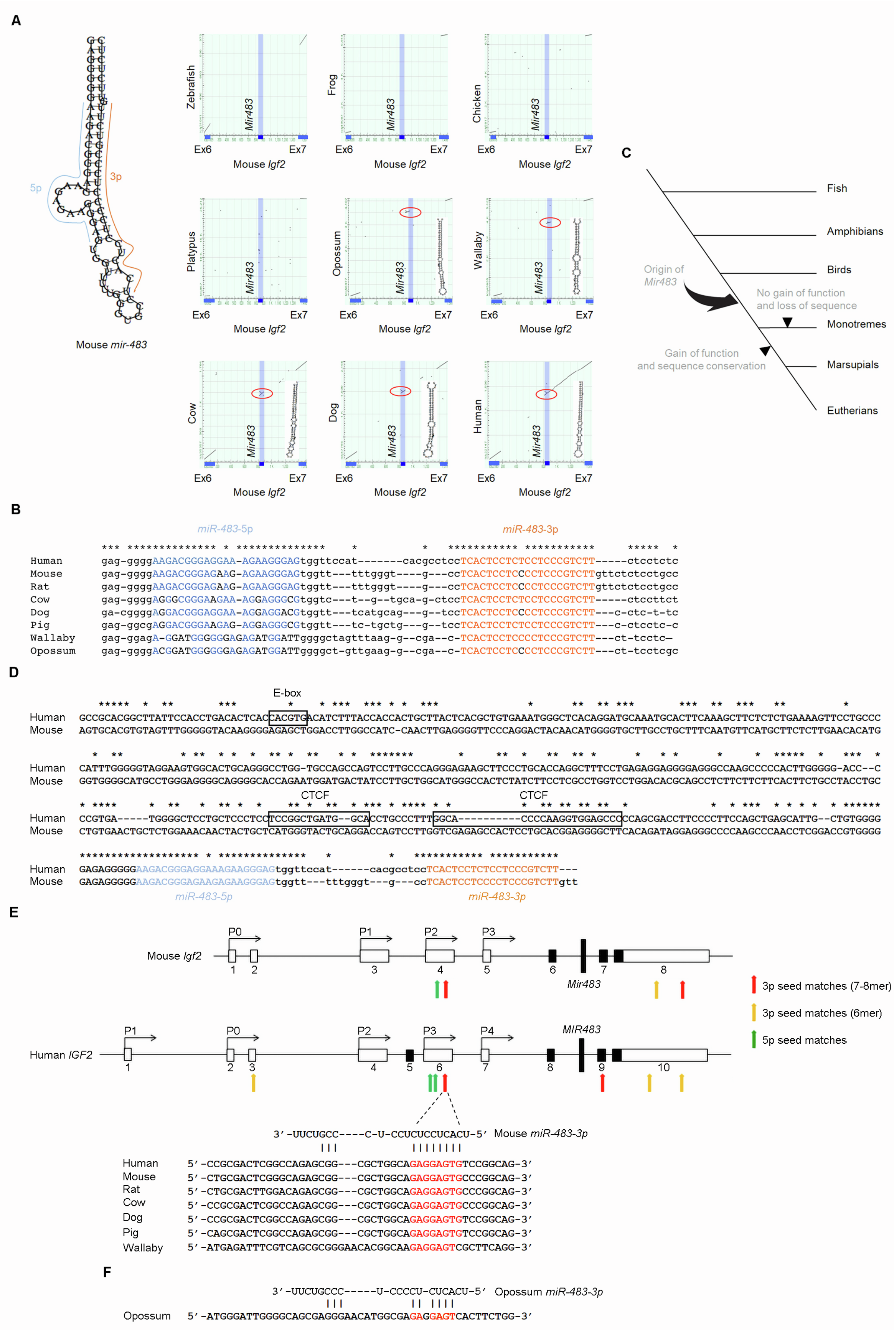
A
Mouse mir-483
5p
3p
Zebrafish
Frog
Chicken
Platypus
Opossum
Wallaby
Cow
Dog
Human
Mir483
Ex6
Ex7
Mouse Igf2
C
Fish
Amphibians
Birds
Origin of Mir483
No gain of function and loss of sequence
Monotremes
Gain of function and sequence conservation
Marsupials
Eutherians
B
miR-483-5p
miR-483-3p
Human gag-ggggAAGACGGGAGGAA-AGAAGGGAGtggttccat-------cacgcctccTCACTCCTCTCCTCCCGTCTT-----ctcctctc
Mouse gag-ggggAAGACGGGAGAAG-AGAAGGGAGtggtt---tttgggt----g---ccTCACTCCTCCCCTCCCGTCTTgttctctcctcgcc
Rat gag-ggggAAGACGGGAGAAG-AGAAGGGAGtggtt---tttgggt----g---ccTCACTCCTCCCCTCCCGTCTTgttctctcctcgcc
Cow gag-ggggAGGGCGGGAAGAA-AGGAGGGCGtggtc---t---g--tgca-g-ctccTCACTCCTCTCCTCCCGTCTT-----ctccttct
Dog ga-cggggAGGACGGGAGGAA-AGGAGGACGtggtt---tcatgcag---g--tccTCACTCCTCCCCTCCCGTCTT---c-ctc-t-tc
Pig gag-ggcgAGGACGGGAAGAG-AGGAGGGCGtggtt---tc-tgctg---g--tccTCACTCCTCTCCTCCCGTCTT---c-ctcctc-c
Wallaby gag-ggagA-GGATGGGGGAGAGATGGATTggggctagtttaag-g--cga--c-TCACTCCTCCCCTCCCGTCTT---ct-tcctc–
Opossum gag-ggggACGGATGGGGGAGAGATGGATTggggct-gttgaag-g--cga--c-TCACTCCTCCCCTCCCGTCTT---ct-tcctcgc
D
E-box
CTCF
CTCF
Human GCCGCACGGCTTATTCCACCTGACACTCACCACGTGACATCTTTACCACCACTGCTTACTCACGCTGTGAAATGGGCTCACAGGATGCAAATGCACTTCAAAGCTTCTCTCTGAAAAGTTCCTGCCC
Mouse AGTGCACGTGTAGTTTGGGGGTACAAGGGGAGAGCTGGACCTTGGCCATC-CAACTTGAGGGGTTCCCAGGACTACAACATGGGGTGCTTGCCTGCTTTCAATGTTCATGCTTCTCTTGAACACATG
Human CATTTGGGGGTAGGAAGTGGCACTGCAGGGCCTGG-TGCCAGCCAGTCCTTGCCCAGGGAGAAGCTTCCCTGCACCAGGCTTTCCTGAGAGGAGGGGGAGGGCCAAGCCCCCACTTGGGGG-ACC--C
Mouse GGTGGGGCATGCCTGGGAGGGGCAGGGGCACCAGAATGGATGACTATCCTTGCTGGCATGGGCCACTCTATCTTCCTCGCCTGGTCCTGGACACGCAGCCTCTTCTTCTTCACTTCTGCCTACCTGC
Human CCGTGA-----TGGGGCTCCTGCTCCCTCCTCCGGCTGATG--GCACCTGCCCTTTGGCA----------CCCCAAGGTGGAGCCCCCAGCGACCTTCCCCTTCCAGCTGAGCATTG--CTGTGGGG
Mouse CTGTGAACTGCTCTGGAAACAACTACTGCTCATGGGTACTGCAGGACCAGTCCTTGGTCGAGAGCCACTCCTGCACGGAGGGGCTTCACAGATAGGAGGGCCCCAAGCCCAACCTCGGACCGTGGGG
Human GAGAGGGGGAAGACGGGAGGAAAGAAGGGAGtggttccat------cacgcctccTCACTCCTCTCCTCCCGTCTT---
Mouse GAGAGGGGGAAGACGGGAGAAGAGAAGGGAGtggtt---tttgggt---g---ccTCACTCCTCCCCTCCCGTCTTgtt
miR-483-5p
miR-483-3p
E
Mouse Igf2
P0
P1
P2
P3
1
2
3
4
5
6
7
8
Mir483
3p seed matches (7-8mer)
3p seed matches (6mer)
5p seed matches
Human IGF2
P1
P0
P2
P3
P4
MIR483
1
2
3
4
5
6
7
8
9
10
3′-UUCUGCC----C-U-CCUCUCCUCACU-5′ Mouse miR-483-3p
Human 5′-CCGCGACTCGGCCAGAGCGG---CGCTGGCAGAGGAGTGTCCGGCAG-3′
Mouse 5′-CTGCGACTCGGGCAGAGCGG---CGCTGGCAGAGGAGTGCCCGGCAG-3′
Rat 5′-CTGCGACTTGGACAGAGCGG---CGCTGGCAGAGGAGTGCCCGGCAG-3′
Cow 5′-CCGCGACTCGGCCAGAGCGG---CGCTGGCAGAGGAGTGCCCGGCAG-3′
Dog 5′-CCGCGACTCGGCCAGAGCGG---CGCTGGCAGAGGAGTGTCCGGCAG-3′
Pig 5′-CAGCGACTCGGCCAGAGCGG---CGCTGGCAGAGGAGTGTCCGGCAG-3′
Wallaby 5′-ATGAGATTTCGTCAGCGCGGGAACACGGCAAGAGGAGTCGCTTCAGG-3′
F
3′-UUCUGCCC-----U-CCCCU-CUCACU-5′ Opossum miR-483-3p
Opossum 5′-ATGGGATTGGGGCAGCGAGGGAACATGGCGAGAGGAGTCACTTCTGG-3′

**Figure S1. Conservation between species of *Mir483* sequence and putative *Mir483* regulatory elements and target sequences at the *Igf2* locus. Related to Figure 1.**

(A) *Mir483* is conserved among eutherian mammals and marsupials. The Blast2 program (http://www.ncbi.nlm.nih.gov/) was used to investigate the conservation of *Mir483* sequence among vertebrates. Genomic sequences including *Igf2*'s exons 6 and 7, and intron 6 of zebrafish, frog, chicken, platypus, opossum, wallaby, cow, dog, human, and mouse were extracted from public data bases. The mouse genome was aligned with other genomes. *Igf2* exons 6 and 7 of the mouse genome and *Mir483* are highlighted by dark blue on the X axes. Grey dots indicate sequence conservation and those corresponding to *Mir483* sequence are emphasized by red circles. The predicted secondary structures of *mir-483* in eutherian mammals and marsupials were drawn using ViennaRNA (http://www.tbi.univie.ac.at/~ivo/RNA/), with that of mouse being shown in the top left corner of this panel. (B) Sequences of eutherian mammals and marsupials corresponding to *Mir483* were aligned. *miR-483-5p* and *miR-483-3p* are highlighted in blue and orange, respectively, and nucleotides conserved between human and mouse are indicated with stars. In some of the species the annotations for the two arms of *miR-483* are based on *in silico* predictions rather than on experimental evidence. (C) Schematic illustration of the evolution of *Mir483* in vertebrates. *Mir483* emerged in the *Igf2* intron 6 region before the divergence of the therians and monotremes. In the therian lineage, *Mir483* acquired function and became conserved in the course of evolution, whereas in the monotreme lineage, *Mir483* did not gain any function or lost it, as its sequence was not conserved. (D) Sequence alignment of the human *MIR483* promoter with the equivalent sequence in the mouse shows poor conservation. The E-box and CTCF binding sites were previously identified as regulatory elements within the human *MIR483* promoter. (E) Top: schematic representation of conserved *Igf2* 5'UTR and 3'UTR sequences between mouse and human containing *miR-483*-3p and *miR-483*-5p seed sites. Genomic features are not drawn to scale and are for representation purposes only. Bottom: the sequence for a *miR-483*-3p seed site mapping to the untranslated mouse exon 4, driven by the *Igf2*-P2 promoter (equivalent to the untranslated human exon 6 driven by *IGF2*-P3 promoter) is conserved in the eutherian mammals and the marsupial wallaby. (F) In opossum, the putative *miR-483*-3p seed site within *Igf2* is interrupted by a mistmatch and for that reason it is unlikely to be functional.

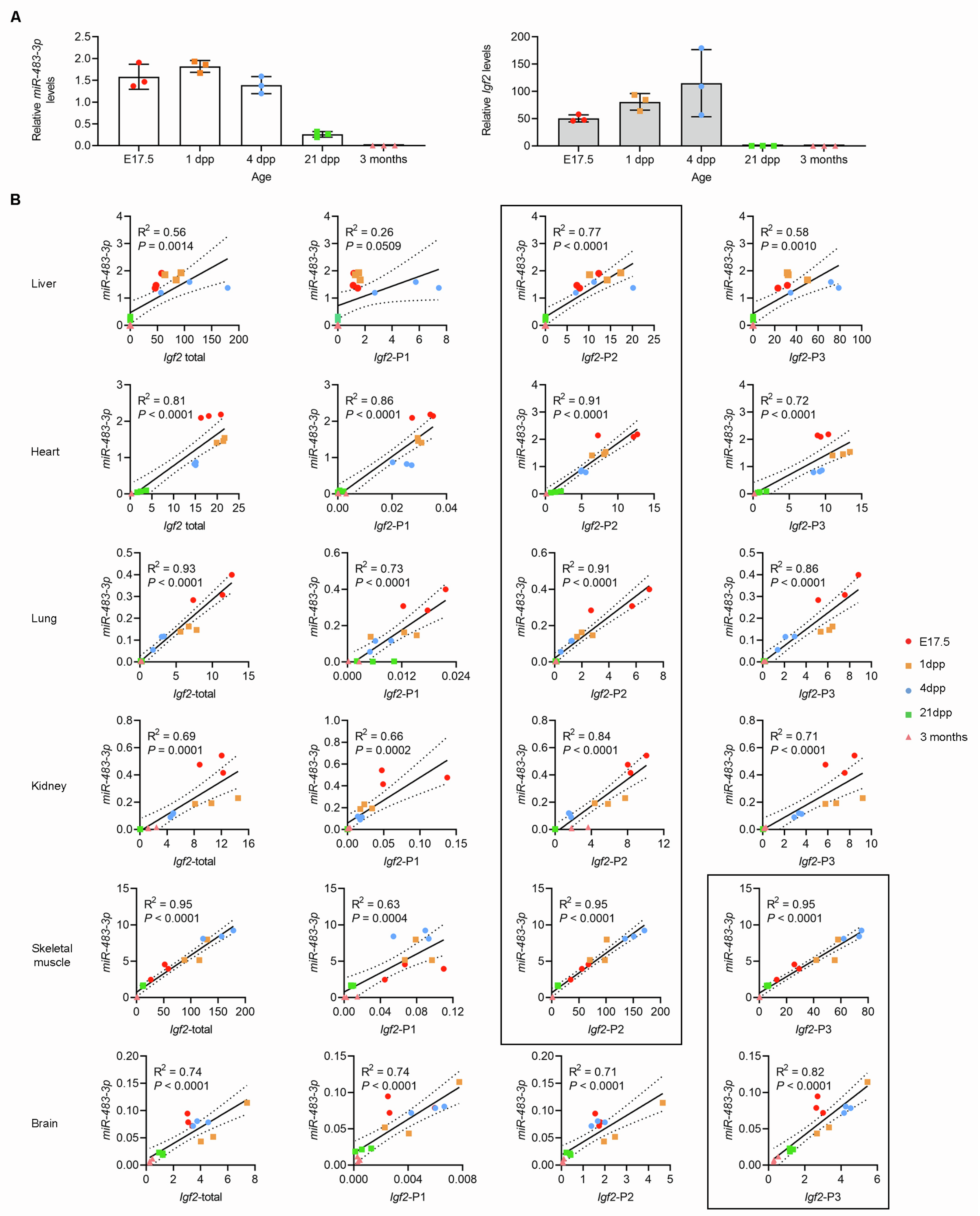


**Figure S2. Developmental expression of *miR-483-3p* compared to *Igf2* in prenatal and postnatal organs. Related to Figure 1.**

(A) Levels of both *miR-483-3p* and *Igf2* in the liver reach a peak in perinatal life and decrease rapidly around weaning, with very low expression levels found in adult life. (B) Linear correlation coefficients ($R^2$) between *miR-483-3p* and *Igf2* transcripts are strongest for the *Igf2*-P2 isoform in

liver, heart, lung and kidney, for *Igf2*-P2 and *Igf2*-P3 in the skeletal muscle and for *Igf2*-P3 in the brain (all highlighted by black contour). For all graphs, expression of *miR-483-3p* was normalized against the geometrical mean of *Snord70*/*snoRNA234*, *Snord68*/*snoRNA202*, and *miR-26b*, and expression of *Igf2* transcript isoforms was normalized against the geometrical means of *Ppia*, *Pmm1*, *Hprt*, *Sdha*, *Tbp* and *Gapdh*. Data are presented as individual values, with averages ± SD in (A) and individual values in (B) (n=3 samples for each developmental time point).

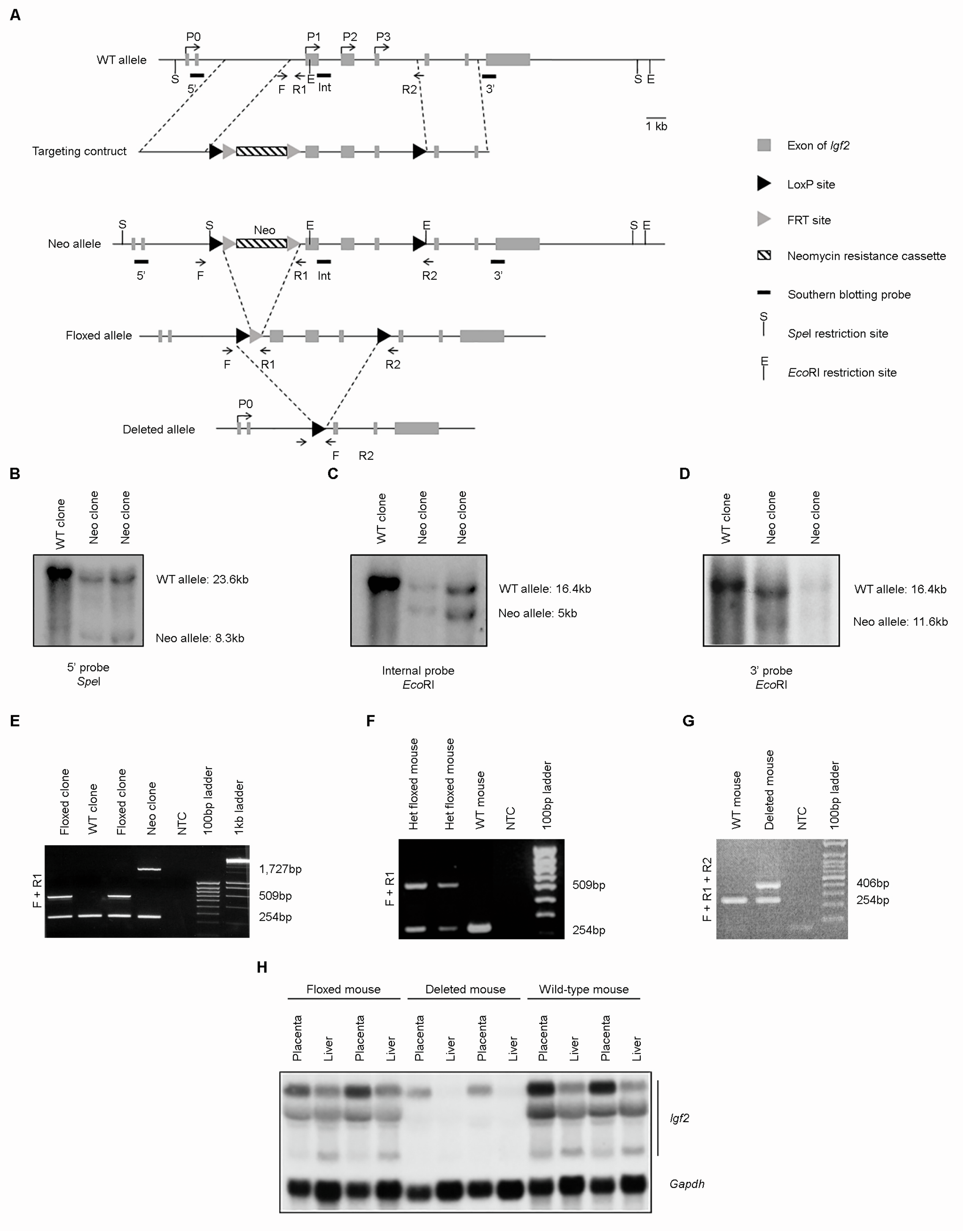


**Figure S3. Generation of the *Igf2*$^{\Delta(P1\text{-}P3)}$ model. Related to Figure 1.**

(A) Diagram showing the wild-type (WT) locus and the targeting construct, followed by the allele configurations after homologous recombination in ES cells, *i.e.* Neo allele, the floxed allele (after removal of the neo gene by Flpe recombinase) and the deleted allele after *Cre*-mediated recombination between LoxP sites. Probes used for Southern blot analysis (5', Int – internal, and 3')

are shown as short horizontal black lines, selected restriction sites are indicated by E (*Eco*RI) or S (*Spe*I) and genotyping primers (F, R1 and R2) are shown by arrows. Genomic features are not drawn to scale and are for representation purposes only. (B) Southern blotting of DNA extracted from ES cell clones and digested with *Spe*I was used to confirm correct 5' targeting. (C) Southern blotting of DNA extracted from ES cell clones and digested with *Eco*RI was used to confirm correct Int targeting. (D) Southern blotting of DNA extracted from ES cell clones and digested with *Eco*RI was used to confirm correct 3' targeting. (E) PCR confirmation of efficient removal of the neomycin cassette by FLPe-FRT recombination using primers F+R1 (NTC – no template control). (F) PCR genotyping of heterozygous floxed mice using primers F+R1. (G) PCR confirmation of efficient deletion of the floxed P1-P3 promoters, using primers F+R1+R2. (H) Northern blot analysis of *Igf2* transcripts and *Gapdh* internal control in placenta and liver at E18.5 (top band observed in placentae of deleted mice corresponds to *Igf2*-P0 transcript that remains expressed upon deletion of the P1-P3 fetal *Igf2* promoters).

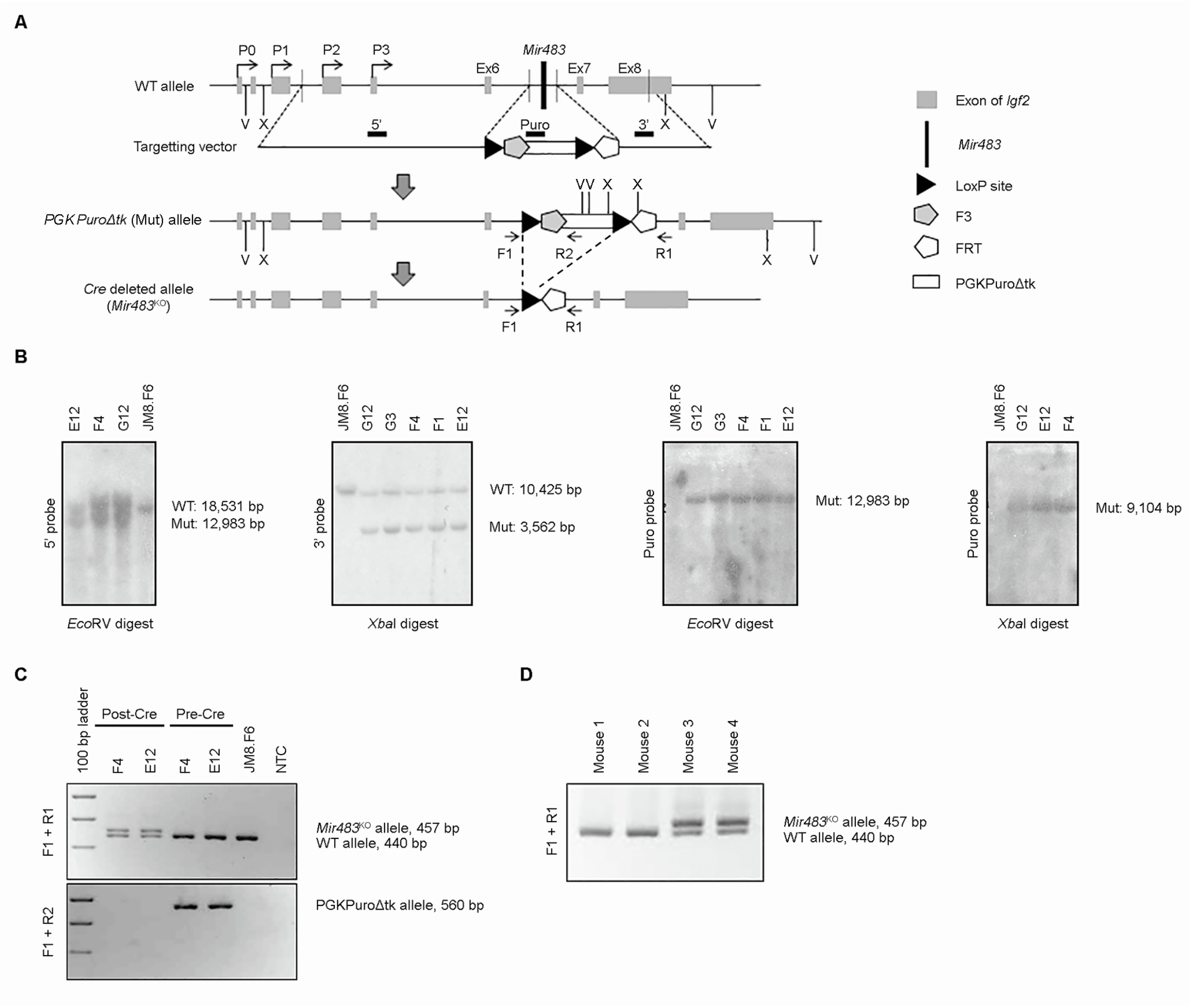


**Figure S4. Generation of the *Mir483* specific knockout. Related to Figure 2.**

(A) Diagram showing the wild-type (WT) locus and the targeting vector, the *PuroΔtk* allele after homologous recombination and the allele after *in vitro Cre* deletion (*Mir483*$^{KO}$). Probes used for Southern blot analysis (5’, Puro and 3’) are shown as short black lines above the targeting vector, selected restriction sites are indicated by V (*Eco*RV) or X (*Xba*I) and genotyping primers (F1, R1 and R2) are shown by arrows. Genomic features are not drawn to scale and are for representation purposes only. (B) Southern blotting of ES cell clones was used to confirm correct targeting. (C) *In vitro Cre* deletion in ES cell clones was confirmed by PCR (using the primers indicated in the figure) before (Pre-*Cre*) and after (Post-*Cre*) transfection with a plasmid encoding a *Cre* recombinase. Parental JM8.F6 ES cell DNA was also amplified, NTC – no template negative control. (D) PCR genotyping in tail DNA using primers F1 and R1.

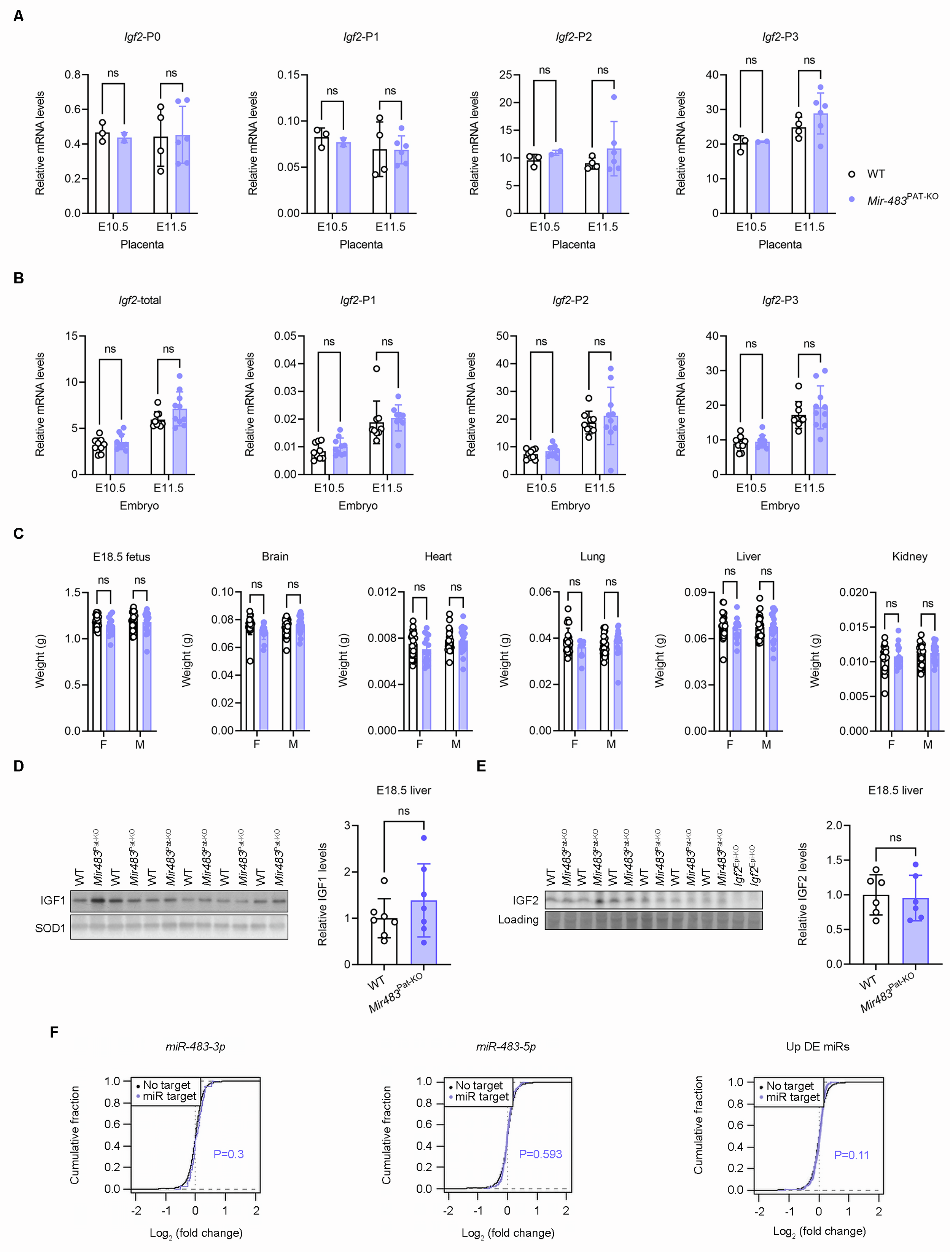


**Figure S5. Expression of *Igf2* isoforms and organ weights and analyses of cumulative fractions mRNA fold changes for predicted targets of DEMs in the *Mir483*$^{\text{Pat-KO}}$ knockout. Related to Figure 2.** (A) Relative mRNA expression of *Igf2* isoforms at E10.5 and E11.5 measured by RT-qPCR levels in placentae (n=2-6 samples per group). Levels of *Igf2* transcripts were normalized against the

geometrical mean of *Gapdh*, *Pmm1* and *Ppia*. (B) Relative expression of total *Igf2* and its isoforms at E10.5 and E11.5 measured by RT-qPCR levels in whole embryos (n=8-10 samples per group). Levels of *Igf2* transcripts were normalized against the geometrical mean of *Gapdh*, *Pmm1* and *Ppia*. (C) Fetus and organ weights at E18.5 (n=15-26 per group; F – females, M – males). (D) IGF1 levels normalized to SOD1 in E18.5 *Mir483*$^{Pat\text{-}KO}$ livers compared to WT littermate controls (left – western blotting, right – quantification; n=6 per group). (E) IGF2 levels normalized to protein loading (as assessed following Coomasie R-250 dye staining) in E18.5 *Mir483*$^{Pat\text{-}KO}$ livers compared to WT littermate controls (left – western blotting, right – quantification; n=6 per group). The E18.5 *Igf2*$^{EpiKO}$ liver samples were collected from fetuses with epiblast-specific conditional deletion of *Igf2*[76]. (F) Cumulative fractions mRNA fold changes between E18.5 livers of *Mir483*$^{Pat\text{-}KO}$ mutants and WT littermates for conserved putative targets of *miR-483*-3p (left), *miR-483*-5p (middle) or the two up-regulated DEMs *miR-370*-3p and *miR-136*-5p (right). For panels (A) – (E) data are presented as individual values, with averages ± SD; ns – non-significant by two-way ANOVA followed by Šídák's multiple comparisons tests in (A) and (B), by Mann-Whitney tests in (D) and (E) and using two-sided Kolmogorov-Smirnov tests in (F).

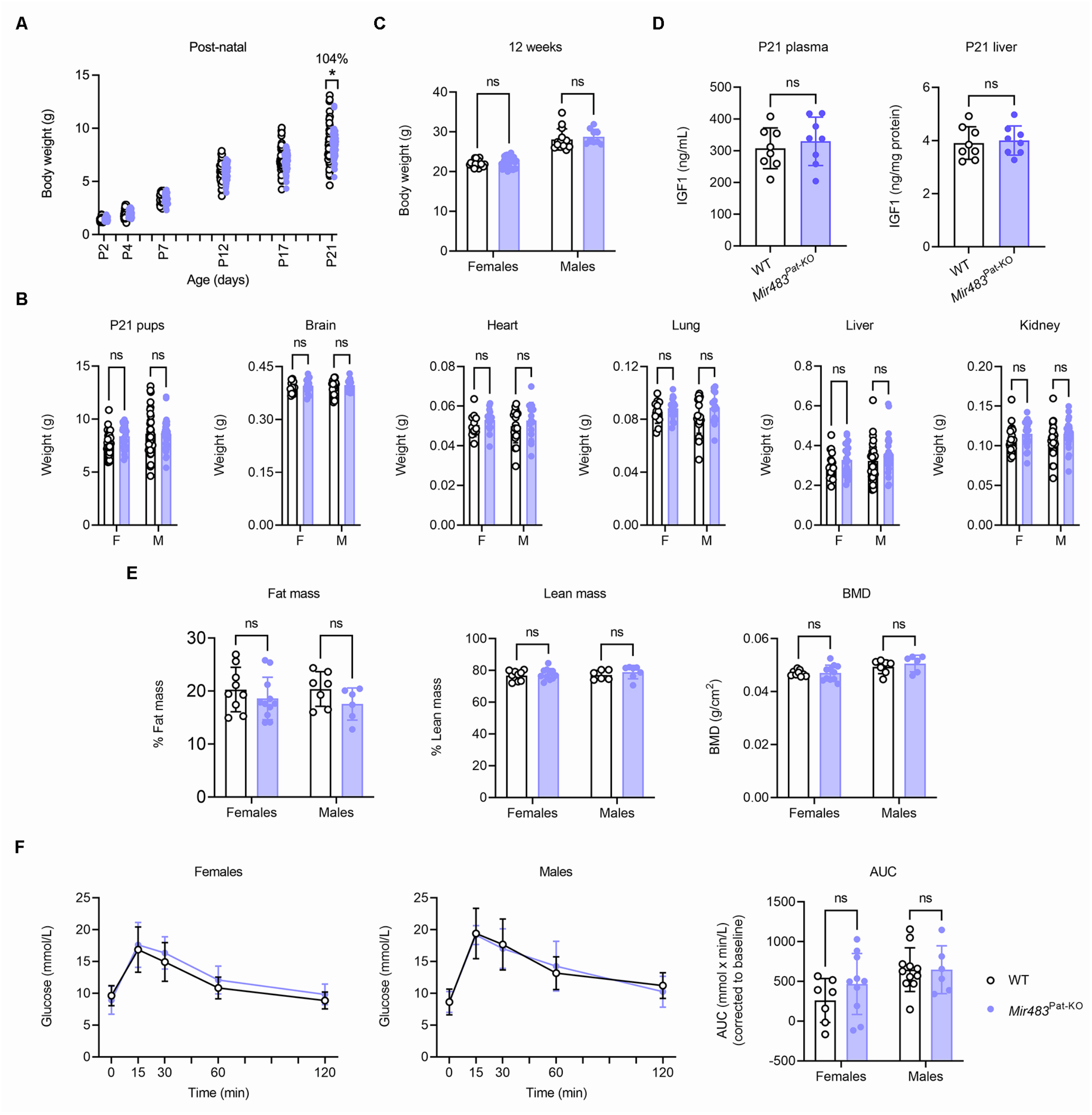


**Figure S6. Postnatal phenotyping in the *Mir483*$^{\text{Pat-KO}}$ knockout model. Related to Figure 2.**

(A) Post-natal growth kinetics from post-partum day 2 (P2), until weaning (P21) (n=11-20 litters at each developmental stage). (B) Total body (n=33-40 per group) and organ weights (n=12-29 per group) at post-natal day 21 (P21). (C) Body weights in 12 weeks-old knockouts and wild-type littermates (n=9-31 per group). (D) IGF1 protein levels measured at P21 by ELISA in plasma (left) or liver (right) of *Mir483*$^{\text{Pat-KO}}$ mutants and WT littermate controls (n=8 per group). For liver, IGF1 levels were normalized against the total protein content measured by a BCA protein assay. (E) Body composition (% fat mass, % lean mass and bone mineral density – BMD) measured by dual energy x-ray absorptiometry (DEXA) at the age of 12 weeks (n=6-11 per group). (F) Glucose tolerance tests with glucose administered by intra-peritoneal injections (ipGTTs) after overnight fasting in females

(n=7-10/genotype) and males (n=6-11/genotype). First two panels show changes in blood glucose concentrations (y-axis), from basal pre-treatment values, with time (x-axis), after glucose administration. The graph on the far right shows area under curve (AUC) calculated during ipGTTs using the trapezoid rule and normalised to basal glucose levels. Data are presented as individual values, with averages ± SD in (A – E) and (F) (far-right graph), and averages ± SD in (F) (first two graphs on the left) and % values indicate ratios *Mir483*$^{Pat\text{-}KO}$/WT; ns – non-significant, * P<0.05 by a mixed effects model in (A), two-way ANOVA followed by Šídák's multiple comparisons tests in (B), (C), (E) and (F) (far-right graph), and unpaired *t*-tests with Welch's correction in (D).

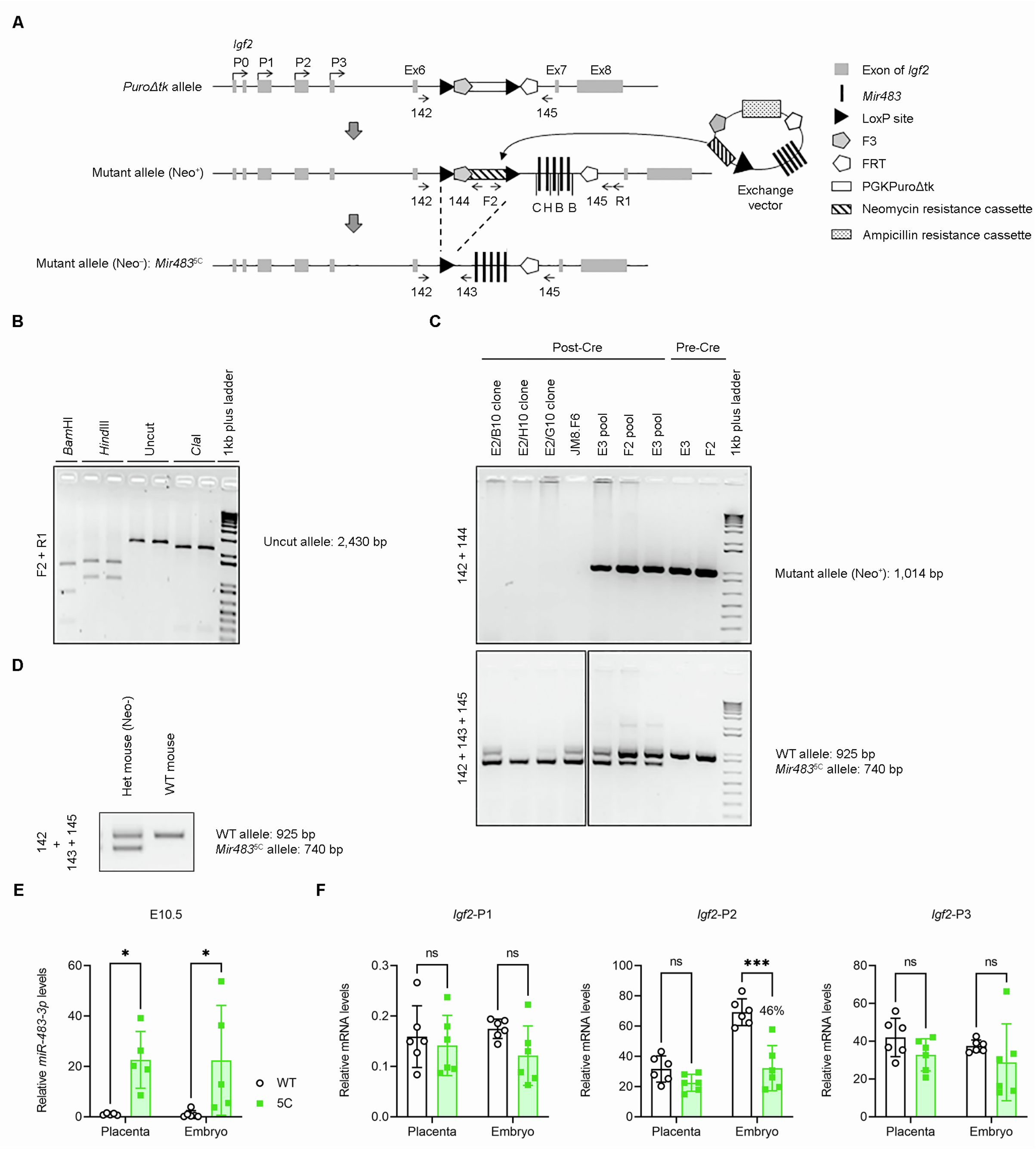


**Figure S7. Generation of *Mir483*$^{5C}$ (5C) mice and down-regulation of *Igf2*-P2 isoform in *Mir483*$^{5C}$ embryos, but not placentae. Related to Figure 3.**

(A) Mice expressing 5 copies of *Mir483* from the endogenous locus were generated by replacement of the PGKPuroΔtk allele of the *Mir483*$^{KO}$ ES cell line (verified for correct and unique integration by Southern blotting as shown in Figure S6) with a neomycin resistance cassette and 5 copies of *Mir483* using RMCE (recombinase-mediated cassette exchange). The diagram shows the PuroΔtk allele and exchange vector, the (Neo$^{+}$) allele after RMCE, and the allele after *in vitro Cre* deletion (Neo$^{-}$). Genotyping primers are depicted by arrows. Selected restriction sites are indicated by C (*Cla*I), E

(*Eco*RI), H (*Hind*III) or B (*Bam*HI). Genomic features are not drawn to scale and are for representation purposes only. (B) PCR using primers F2 and R1 generated a 2,430 bp product indicating that RMCE had occurred and the five copies of *Mir483* were present. The identity of the PCR product was confirmed by digestion with *Bam*HI, *Hind*III or *Cla*I. (C) *In vitro Cre* deletion in ES cell clones was determined by PCR (using the primers indicated in the figure) before (Pre-Cre) and after (Post-Cre) transfection with a plasmid encoding a *Cre* recombinase. DNA pools of ESC clones and parental JM8.F6 ESC DNA were also amplified. (D) Mice were then routinely genotyped by PCR, using primers 142, 143 and 145. (E) Relative expression of *miR-483-3p* measured by RT-qPCR in whole embryo lysates at E10.5 (n=5-6 samples/group). Levels of *miR-483-3p* were normalized against the geometrical mean of *Snord70*/*snoRNA234*, *Snord68*/*snoRNA202*, and are presented relative to the wild-type (WT) levels, arbitrarily set to 1. (F) Relative expression of *Igf2* isoforms at E11.5 measured by RT-qPCR levels in placenta and embryo (n=6 samples per group). For panels (E) and (F) levels of *Igf2* transcripts were normalized against the geometrical mean of *Gapdh*, Sdha and *Pmm1*. Data are presented as individual values, with averages ± SD and % indicate ratios 5C/WT; ns – non-significant, * $P<0.05$ and *** $P<0.001$ by two-way ANOVA followed by Šídák's multiple comparisons tests.

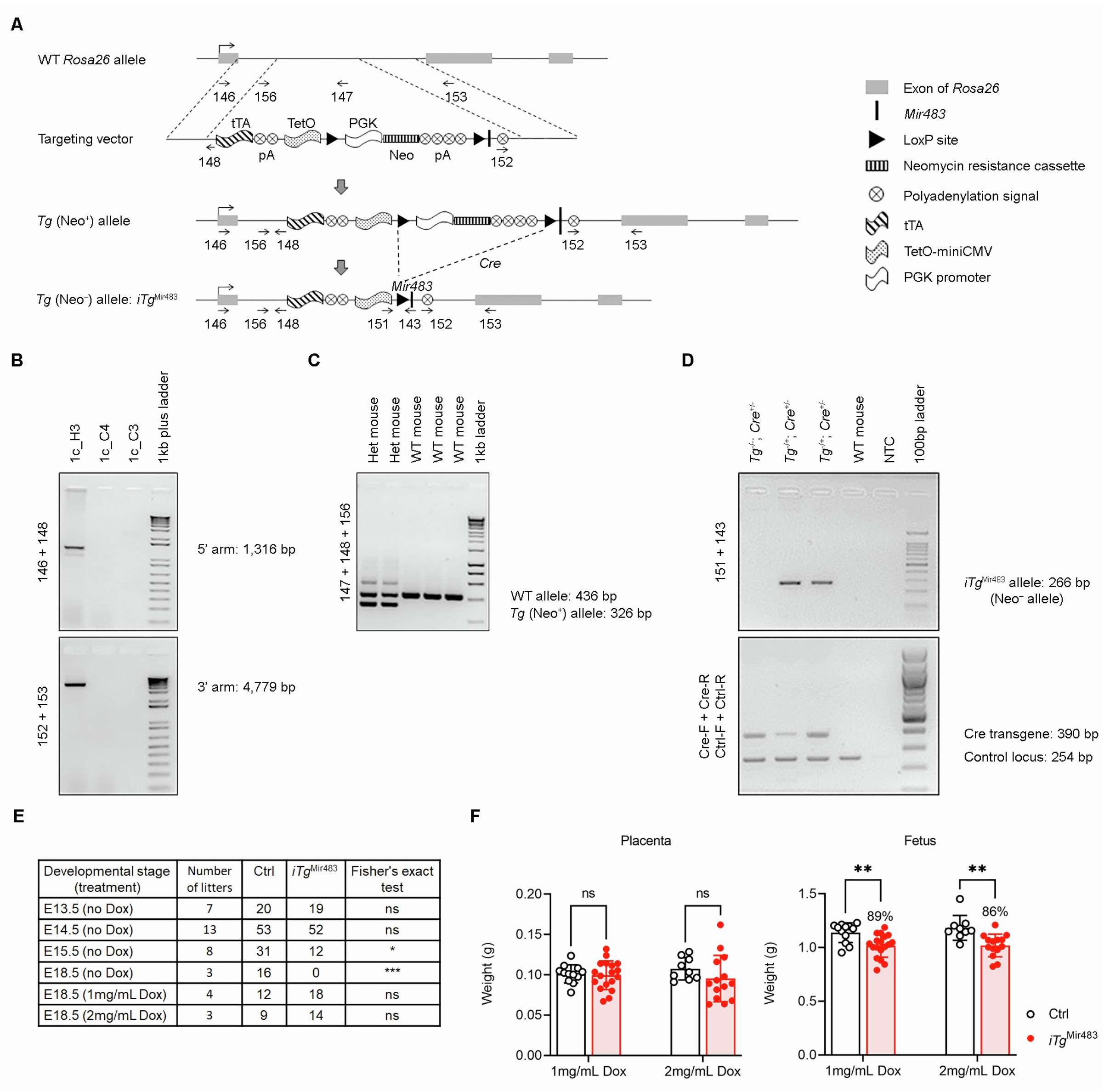


| Developmental stage (treatment) | Number of litters | Ctrl | iTgMir483 | Fisher's exact test |
|---|---|---|---|---|
| E13.5 (no Dox) | 7 | 20 | 19 | ns |
| E14.5 (no Dox) | 13 | 53 | 52 | ns |
| E15.5 (no Dox) | 8 | 31 | 12 | * |
| E18.5 (no Dox) | 3 | 16 | 0 | *** |
| E18.5 (1mg/mL Dox) | 4 | 12 | 18 | ns |
| E18.5 (2mg/mL Dox) | 3 | 9 | 14 | ns |

**Figure S8. Generation of TET-OFF inducible transgenic mice with an additional copy of *Mir483* inserted at the *Rosa26* locus (*iTg*$^{Mir483}$), with total rescue of *iTg*$^{Mir483}$ lethality and partial rescue of fetal growth restriction by doxycycline administration. Related to Figure 4.**

(A) Diagram showing the wild-type *Rosa26* locus, the targeting vector containing a tetracycline-controlled transactivator (tTA), transgenic (Tg) (Neo$^{+}$) allele after homologous recombination in ES cells and the Tg allele after *Cre* deletion (Neo$^{-}$). Genotyping primers are shown by arrows. Genomic features are not drawn to scale and are for representation purposes only. (B) ES cell clones were screened by PCR; homologous recombination of the 5' arm and 3' arm was assayed using primers 146+148 and 152+153, respectively. The gel image depicts one targeted clone (1c_H3) and two non-targeted clones. (C) Mice were then routinely genotyped using primers 147, 148 and 156. (D) Males heterozygous for the Neo$^{+}$ transgene were mated with female mice homozygous for a *CMV-Cre*

transgene, to generate Neo$^{-}$ progeny (*iTg*$^{Mir483}$), which were genotyped using primers 151 and 143 (across the deleted region) and Cre-F, Cre-R, Ctrl-F and Ctrl-R (to detect the presence of *Cre*), NTC – no template negative control. (E) Maternal doxycycline administration in drinking water (Dox, 1mg/mL or 2 mg/mL) throughout gestation rescues the post-mid-gestation lethality of *iTg*$^{Mir483}$ conceptuses. The table shows the distribution of live conceptuses per genotype identified at various developmental time-points during gestation. (F) Placenta and fetal weights at E18.5 upon maternal administration of Dox (n=9-18/group). Data in panel (F) are presented as individual values, with averages ± SD in and % indicate ratios *iTg*$^{Mir483}$/Ctrl; ns – non-significant, * $P$<0.05, ** $P$<0.01 and *** $P$<0.001 by Fisher's exact tests in (E) or two-way ANOVA followed by Šídák's multiple comparisons tests in (F).

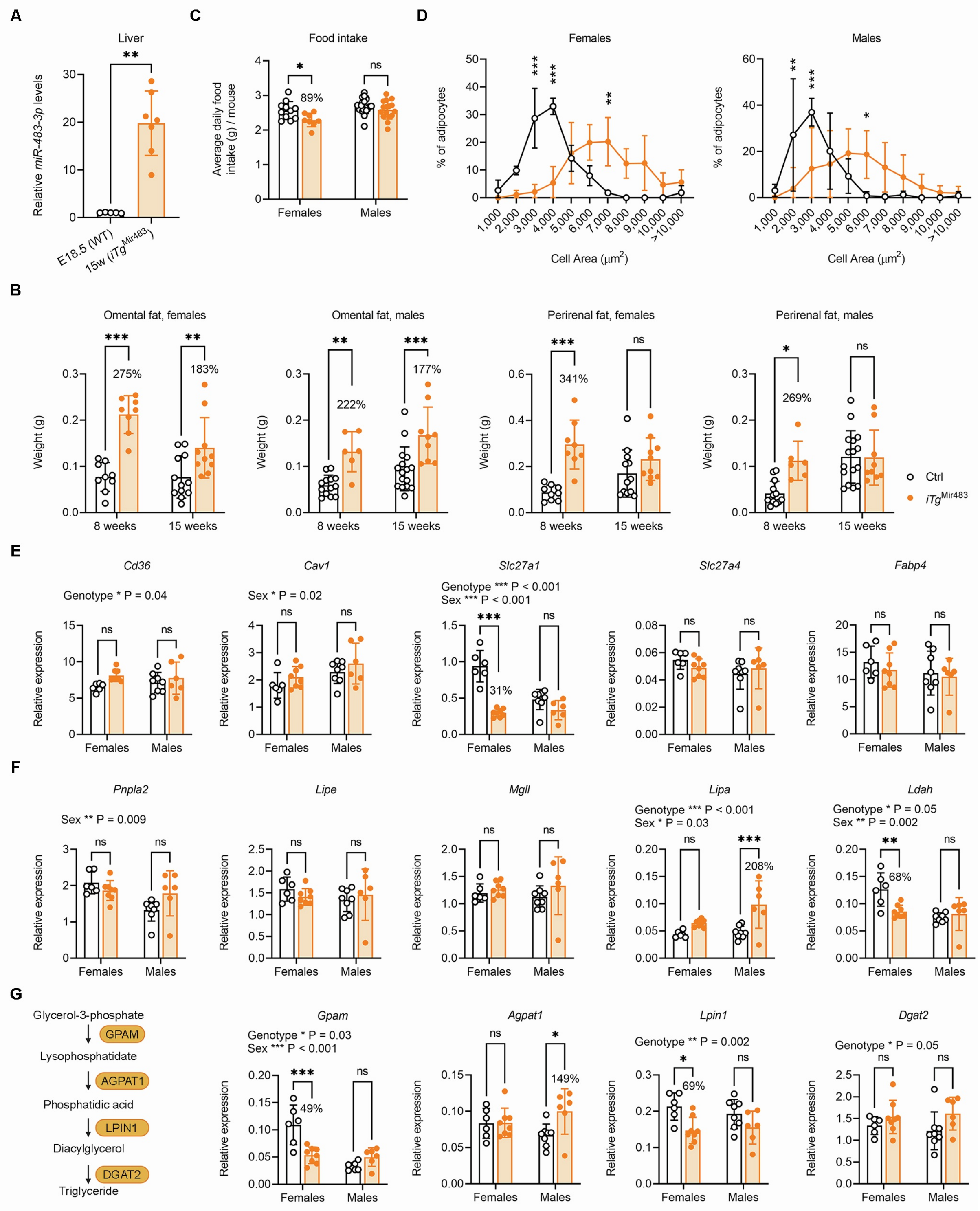


**Figure S9. Postnatal phenotyping the *iTg*^Mir483 mouse model. Related to Figure 5.**

(A) Relative levels of *miR-483*-3p measured by RT-qPCR in liver of 15 week-old *iTG*^Mir483 mutants compared to E18.5 wild-type controls. Levels of *miR-483-3p* were normalized against the geometrical mean of *Snord70*/*snoRNA234* and *Snord68*/*snoRNA202* (n=5-7 samples/group). (B) Omental fat pads are significantly heavier in week 8 (W8) and W15 in *iTG*^Mir483 adults compared to

age-matched controls, while peri-renal fat pads are significantly heavier only at W8 (n=6-16 per group). (C) Average daily food intake, measured between W3 and W4 is significantly lower in *iTG*$^{Mir483}$ females compared to age-matched controls, with no significant differences between the two genotypes in males (n=8-20 per group). (D) Distribution of adipocyte cell area isolated from the gonadal fat pad of W8 *iTG*$^{Mir483}$ and age-matched controls indicates larger adipocytes in mutants of both sexes (n=2-10 per group). (E) Expression patterns of genes encoding lipid transporters are largely unchanged in adipocytes isolated from gonadal fat of W8 *iTG*$^{Mir483}$ and age-matched controls, with the notable expression of *Slc27a1* (also known as *Fatp1*) that is significantly down-regulated in mutant females only (n=6-8 per group). (F) Expression patterns of genes encoding three major lipases (*Pnlpa2*, also known as *Atgl*; *Lipe*, also known as *Hsl*; *Mgll*, also known as *Mgl*) are unchanged in adipocytes isolated from gonadal fat of W8 *iTG*$^{Mir483}$ and age-matched controls. Significant differences were observed for two minor lipases: *Lipa*, upregulated in males, and *Ldah*, down-regulated in females (n=6-8 per group). (G) Left: diagram depicting the steps involved in the conversion of glycerol-3-phosphate (G3P) into triglycerides (TG). Right: expression patterns of genes encoding enzymes implicated in the synthesis of TG in adipocytes isolated from gonadal fat of W8 *iTG*$^{Mir483}$ and age-matched controls (n=6-8 per group). Data are presented as individual values, with averages ± SD in (A), (B), (C), (E) and (G), or averages ± SD in (D) and % indicate ratios *iTg*$^{Mir483}$/Ctrl; ns – non-significant, * *P*<0.05, ** *P*<0.01 and *** *P*<0.001 by a Mann-Whitney test in (A), two-way ANOVA followed by Šídák's multiple comparisons tests in panels (B) – (G). For panels (E), (F) and (G) the effects of genotype and sex identified by two-way ANOVA tests are indicated above the graphs. None of the genes shown in these panels are putative direct targets for *Mir483* regulation, with exception of *Lpin1.*

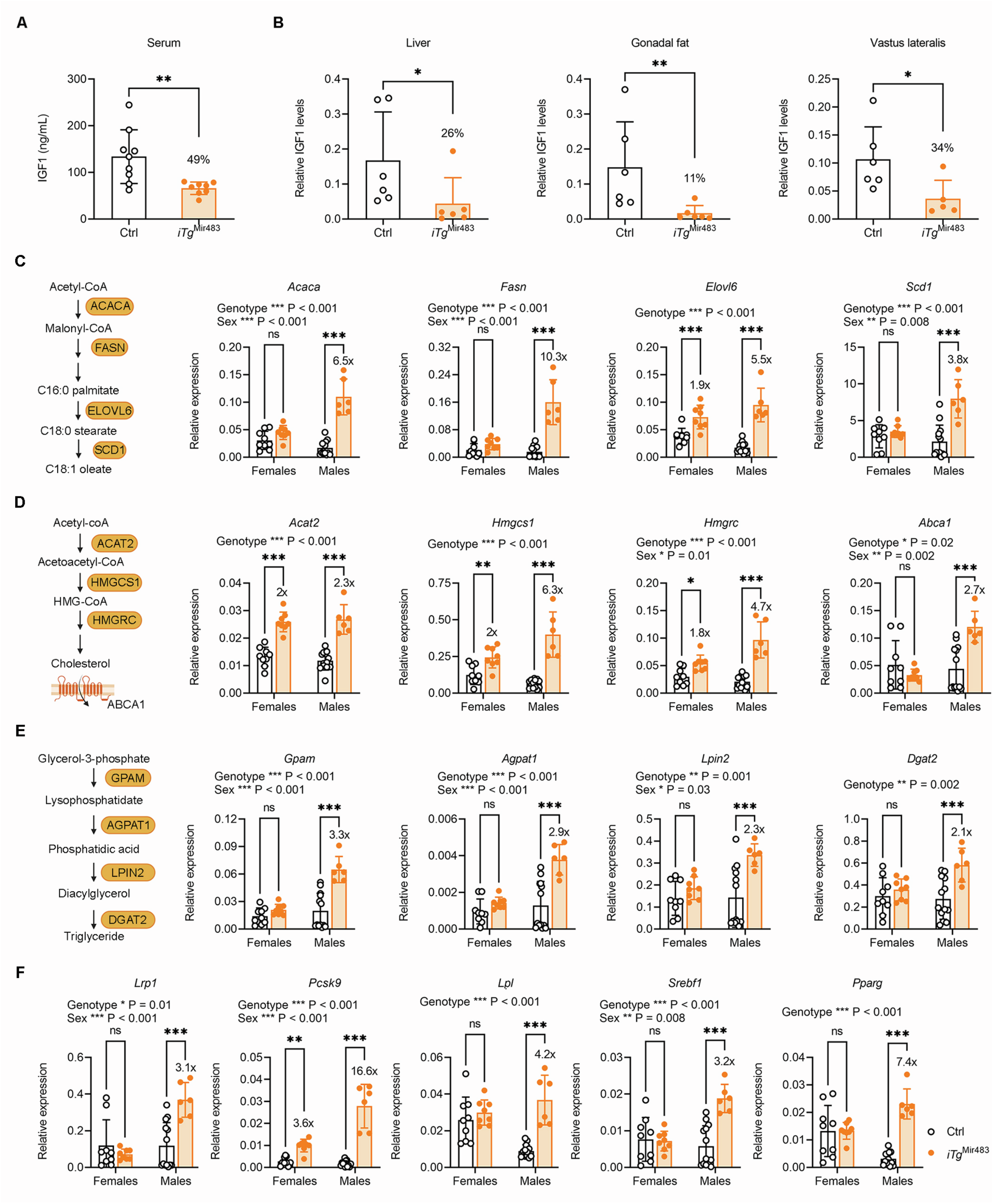


**Figure S10. IGF1 protein levels and mRNA levels of genes that regulate lipid production or trafficking in the liver of the *iTg*$^{\mathrm{Mir483}}$ mouse model. Related to Figure 6.**

(A) IGF1 protein levels measured by ELISA in serum of W3 *iTG*$^{\mathrm{Mir483}}$ and age-matched controls (n=8-9 per group). (B) Relative protein levels of mature IGF1 (7.5 kDa) measured by western blot analyses in protein lysates of three organs collected from W15 males of *iTG*$^{\mathrm{Mir483}}$ and age-matched controls (n=5-6 per group). Levels of IGF1 were normalized against Coomassie staining in the liver and

gonadal fat, and SOD1 in the *vastus lateralis*, used as internal controls for protein loading. (C) mRNA levels of genes encoding key enzymes involved in free fatty acid (FFA) synthesis in the livers of W8 *iTG*$^{Mir483}$ adults compared to age-matched controls (n=6-13 per group). (D) Relative mRNA levels of genes encoding key enzymes involved in cholesterol synthesis in the livers of W8 *iTG*$^{Mir483}$ adults compared to age-matched controls (n=6-13 per group). (E) Relative mRNA levels of genes encoding key enzymes involved in triglyceride synthesis in the livers of W8 *iTG*$^{Mir483}$ adults compared to age-matched controls (n=6-13 per group). (F) Expression patterns of genes encoding proteins implicated in lipoprotein turnover (*Lrp1*, *Pcsk9* and *Lpl*) and key transcriptional factors that regulate lipid synthesis (*Sreb1*, *Pparg*) in livers of W8 *iTG*$^{Mir483}$ and age-matched controls (n=6-13 per group). Data are presented as individual values, with averages ± SD and % or "x" indicate ratios/fold changes *iTg*$^{Mir483}$/Ctrl; ns – non-significant, * $P<0.05$, ** $P<0.01$ and *** $P<0.001$ by an unpaired *t*-test with Welch's correction in (A) Mann-Whitney tests in (B) and two-way ANOVA followed by Šídák's multiple comparisons tests in panel (C) – (F). For panels (C) – (F), the effects of genotype and sex identified by two-way ANOVA tests are indicated above the graphs. None of the genes shown in these panels are putative direct targets for *Mir483* regulation.

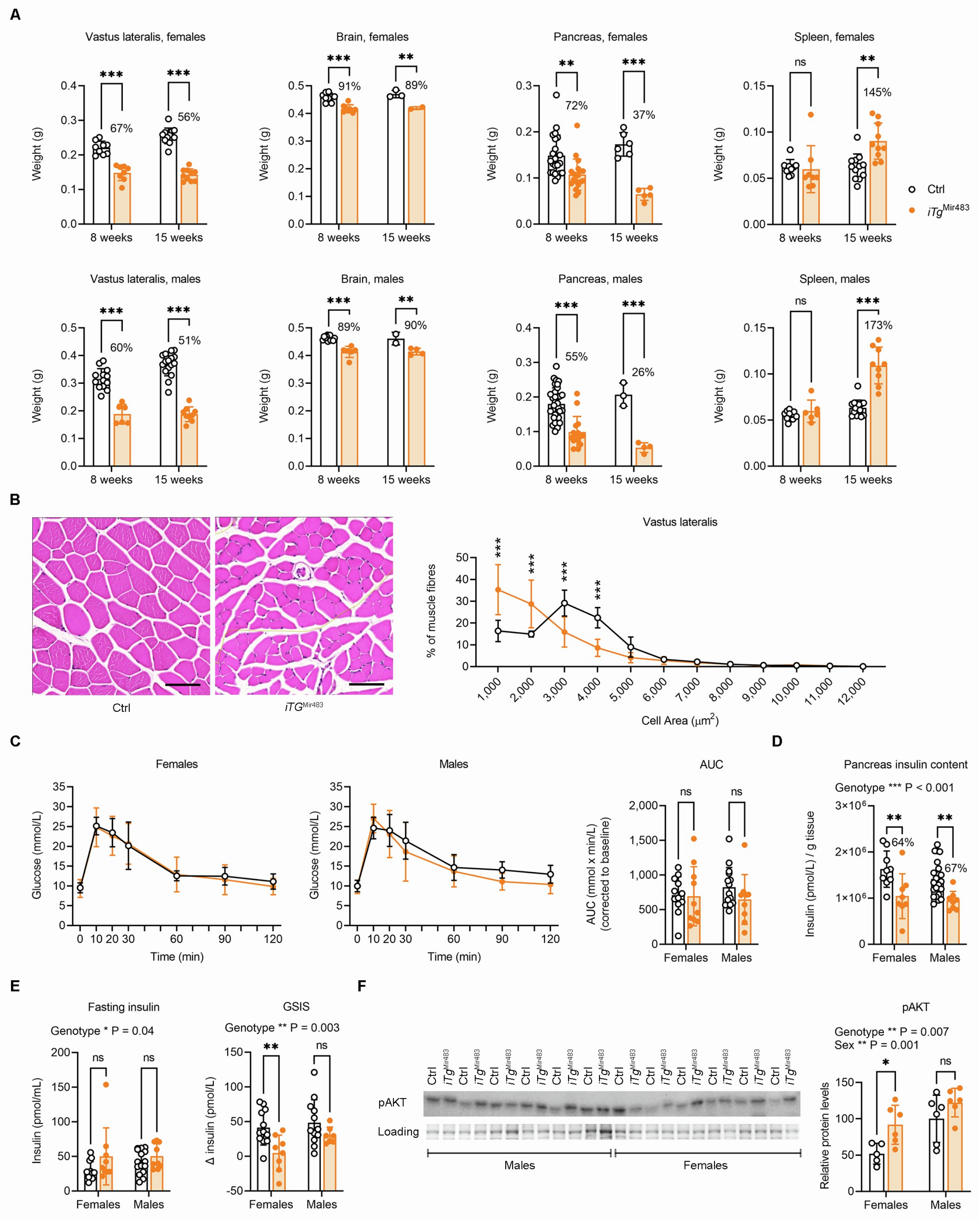


**Figure S11. Organ size, muscle morphology and glucose metabolism assessment in the *iTg*$^{Mir483}$ mouse model. Related to Figure 6.**

(A) Organ weights in W8 and W15 *iTG*$^{Mir483}$ adults compared to age-matched controls (n=2-16 per group). The *vastus lateralis* and pancreas are disproportionally smaller; the brain is proportionally smaller and the spleen is disproportionally bigger, but only at W15. (B) The skeletal muscle fibres are

smaller in the *vastus lateralis* of W15 *iTg*$^{Mir483}$ mutant males compared to age-matched controls (representative H&E stained sections – left, and distribution of muscle fibre area – right; n=5 samples per group, scale bars are 100 µm). (C) Glucose tolerance tests in W13 *iTg*$^{Mir483}$ mutants and age-matched controls, with glucose administered by oral gavage (OGTTs) after six hours fasting performed in females (n=10-12/genotype) and males (n=9-15/genotype). First two panels show changes in blood glucose concentrations (y-axis), from basal pre-treatment values, with time (x-axis), after glucose administration. The graph on the far right shows area under curve (AUC) calculated during OGTTs using the trapezoid rule and normalised to basal glucose levels. (D) Total pancreas insulin content in W18 *iTg*$^{Mir483}$ mutants and age-matched controls after overnight fasting (n=8-22 per group). (E) Left: insulin levels measured in plasma in W13 *iTg*$^{Mir483}$ mutants and age-matched controls, after six hours fasting and prior to the start of the OGTT (n=9-16 samples per group). Right: glucose-stimulated insulin secretion (GSIS) measured in plasma at minute 20 during OGTT (n=7-12 samples per group). (F) pAKT levels normalized to protein loading (as assessed following Coomasie R-250 dye staining) in the gonadal fat of W8 *iTG*$^{Mir483}$ adults compared to age-matched controls (left – western blotting, right – quantification; n=6 per group). Data are presented as individual values, with averages ± SD in (A), (C) (far right), (D), (E) and (F) (right) or as averages ± SD in (B) and (C) (left side), and % indicate ratios *iTg*$^{Mir483}$/Ctrl; ns – non-significant, ** $P<0.01$ and *** $P<0.001$ by two-way ANOVA followed by Šídák's multiple comparisons tests in (A)-(F). For panels (D)-(F), the effects of genotype or sex identified by two-way ANOVA tests are indicated above the graphs.

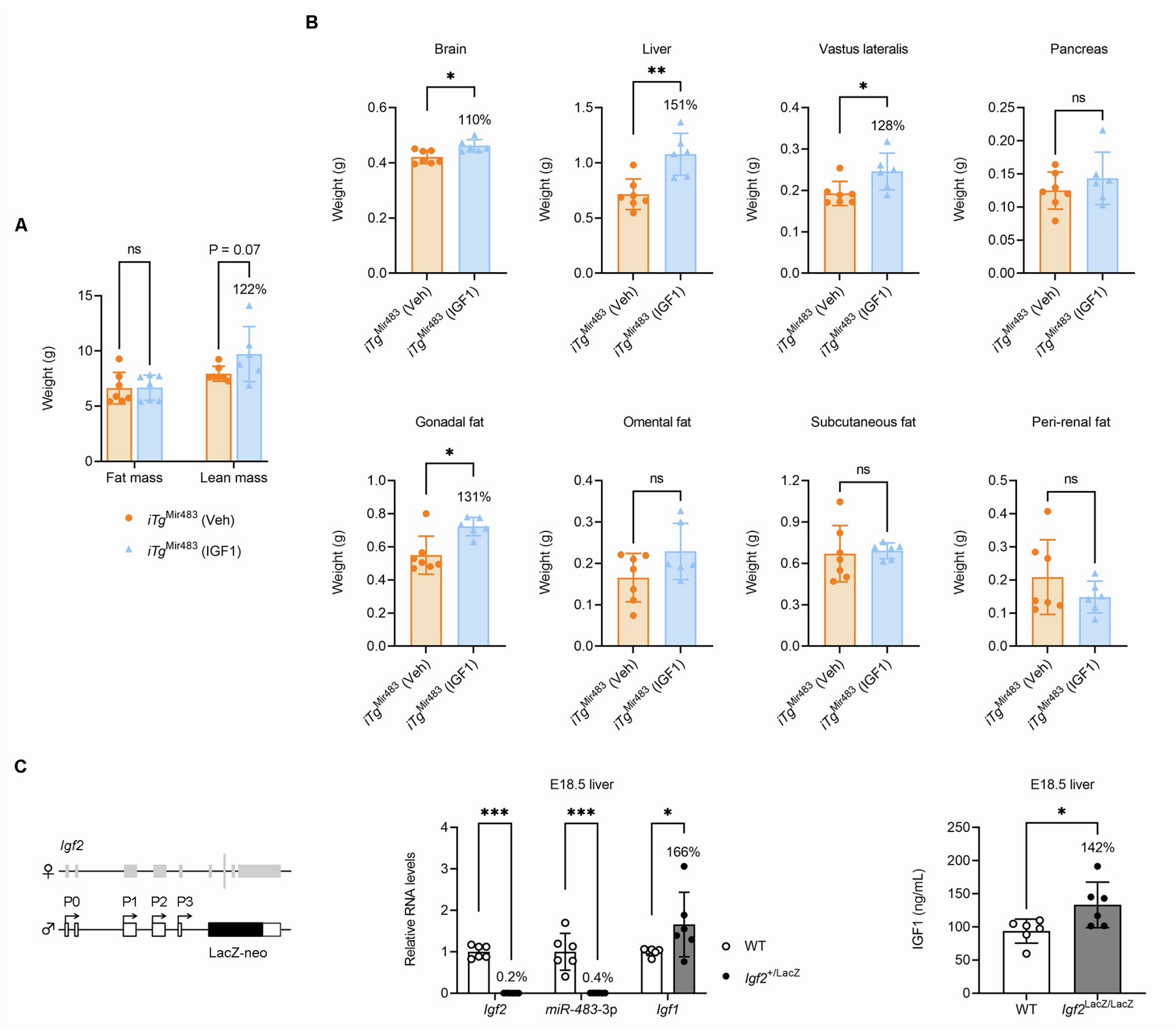


**Figure S12. Impact of IGF1 infusion on body composition in the *iTg*$^{\mathrm{Mir483}}$ mouse model and additional *in vivo* evidence for *Igf1* as target of *miR-483*. Related to Figure 7.**

(A) Body composition measured by TD-NMR in W8 *iTg*$^{\mathrm{Mir483}}$ male mice exposed to vehicle or IGF1 via minipumps (n=6-7 per group). (B) Organ weights in W8 *iTG*$^{\mathrm{Mir483}}$ male mice exposed to vehicle or IGF1 via minipumps (n=6-7 per group). (C) Left: schematic representation of the *Igf2*$^{+/\mathrm{LacZ}}$ model in which the coding exons 4-6 of *Igf2* and *Mir483* are replaced by a LacZ cassette. Genomic features are not drawn to scale and are for representation purposes only. Middle: relative RNA levels for *Igf2*, *miR-483-3p* and *Igf1* in the liver of E18.5 *Igf2*$^{+/\mathrm{LacZ}}$ mutants and WT littermate controls (data was normalized against the geometrical means of *Ppia* and *Gapdh* for *Igf2* and *Igf1*, and against *Snord70/snoRNA234* for *miR-483*-3p; n=6 samples per group). Right: measurement of IGF1 protein by ELISA in the liver of E18.5 *Igf2*$^{\mathrm{LacZ/LacZ}}$ mutants and WT littermate controls (n=6 per group). Data are presented as individual values with averages ± SD and % values indicate ratios *iTG*$^{\mathrm{Mir483}}$ (IGF1)/ *iTG*$^{\mathrm{Mir483}}$ (Veh) in (A) and (B) or *Igf2*$^{+/\mathrm{LacZ}}$/WT or *Igf2*$^{\mathrm{LacZ/LacZ}}$/WT in c; ns – non-significant, * $P$<0.05, **

*P*<0.01 and *** *P*<0.001 by two-way ANOVA followed by Šídák's multiple comparisons tests in (A) and (C) (middle), or Mann-Whitney tests in (B) and (C) (right).

**Table S5. Overview of developmental defects observed by HREM (High-Resolution Episcopic Microscopy) in $iTg^{Mir483}$ embryos at E14.5 (n=6). Related to Figure 4.**

| Organ/system | Specific defect | Frequency |
|---|---|---|
| Cardio-vascular | Atrium and AV junction defects | 100% |
| | Double outlet right ventricle (DORV) with associated ventricle septum defect | |
| | Malformations of intrathoracic arteries (interruption of aortic arch, aortic coarctation, right sided aortic arch, left sided lusoria artery, connections between subclavian artery and pulmonary trunk) | |
| | Abnormalities of head arteries | |
| | Abnormalities of the ductus venosus and portal vein | |
| Urogenital tract | Abnormal remodeling of the metanephrotic tissue and its ducts | 83% |
| | Abnormalities of kidneys or ureters (abnormal pelvis renalis to absent ureter and additional ureteral buds) | |
| | Malformations of the Wolff and Müller ducts | |
| Skeleton | Absent or abnormal acromion with abnormal acromioclavicular joints | 83% |
| | Abnormal otic vesicles | |
| | Thoracoschisis | |
| | Abnormal nasal cavities and associated head bones | |
| | Abnormal tail morphology | |
| Thymus | Abnormal topology of the thymus | 67% |
| Liver/bile ducts | Enlarged sinusoidal spaces | 67% |
| | Additional ducts branching from the cystic duct to enter the liver | |
| Eye and eye muscle | Eyes with missing lenses | 67% |
| | Eye muscle abnormalities | |
| | Retro-lental blood | |
| Nervous system | Brain defects | 50% |
| | Holoprosencephaly | |
| | Smaller superior cervical ganglion | |
| Thyroid | Unilateral absence of the thyroid gland lobe | 33% |

**Table S7. Primers and TaqMan probes used for genotyping mouse strains by PCR and RT-qPCR**

| Primers used for genotyping mouse strains by PCR | | | | | |
|---|---|---|---|---|---|
| Mouse strain | Primer | Sequence (5'-3') | Primer | Sequence (5'-3') | Amplicon (bp) |
| *Igf2*$^{\Delta(P1\text{-}P3)}$ | F | ATGTCTCCAATCCTTGAACACTG | R1<br>R2 | GCAGTGGGAGAAATCAGAACC<br>GCTTTTTTAGTGGTGGGAGGC | WT – 254<br>Floxed – 509<br>Del – 406 |
| *H19*$^{\Delta13}$ | F | TGCCACAGAGGAA-GAAACCAG | R1<br>R2 | AGTCATAGCCGAATAGCC<br>TTCAGTCACTTCCCTCAGCCTC | Δ13 – 895<br>WT – 494 |
| *Mir483*$^{KO}$ | F1 | TACCTGCCTGTGAACTGCTCTG | R1 | ATCTGGTGCCTCCTGTCTGGTA | WT – 440<br>KO – 457 |
| *Mir483*$^{5C}$ | 142 | CACGCTTCAGTTTGTCTGTTCG | 143<br>145 | AAGAATCGATACCGTCGACCTC<br>CTGGAGTGGTTTGGAAAACAGG | WT – 925<br>5C – 740 |
| *iTg*$^{Mir483}$ | 156<br><br>151 | TCCCAAAGTCGCTCTGAGTT<br><br>AGGGAGTGGTAAACTCGACC | 147<br>148<br>143 | GGCGGATCACAAGCAATAAT<br>GAAAGACCGCGAAGAGTTTG<br>AAGAATCGATACCGTCGACCTC | WT – 436<br>Neo$^{+}$ – 326<br>Neo$^{-}$ – 266 |
| *CMV*-Cre | Cre-F<br>Ctrl-F | CGAGTGATGAGGTTCGCAAG<br>ATGTCTCCAATCCTTGAACACTG | Cre-R<br>Ctrl-R | TGAGTGAACGAACCTGGTCG<br>GCAGTGGGAGAAATCAGAACC | Cre – 340<br>WT – 254 |
| *Igf2*$^{KO}$ | F | TTACAGTTCAAAGCCACCACG | R1<br>R2 | GCCAAAGAGATGAGAAGCACC<br>GCCAAACACAGTAAAAAGAAAT<br>GC | WT – 324<br>Floxed – 449<br>Del – 384 |
| *Igf2*$^{LacZ}$ | F | TCCTCAAGGGTTTCTTACAGTTC | R1<br>R2 | CCTCGACTAAACACATGTAAAGC<br>GACAAACTGAAGCGTGTCAAC | WT – 420<br>LacZ – 807 |
| Primers and TaqMan probes used for RT-qPCR | | | | | |
| Gene | Primer | Sequence (5'-3') | Primer | Sequence (5'-3') | Amplicon (bp) |
| *miR-483-3p* | mmu481853_mir (ThermoFisher Scientific #A25576) | | | | |
| *miR-483-5p* | mmu481180_mir (ThermoFisher Scientific #A25576) | | | | |
| *Snord70/ snoRNA234* | 001234 (ThermoFisher Scientific #4427975) | | | | |
| *Snord68/ snoRNA202* | 001232 (ThermoFisher Scientific #4427975) | | | | |
| *miR-26b* | mmu481662_mir (ThermoFisher Scientific # A25576) | | | | |
| *Igf2* | F (Ex7) | AGTCCGAGAGGGACGTGTCTA | R (Ex8) | CGGACTGTCTCCAGGTGTCAT | 102 |

| | | | | | |
|---|---|---|---|---|---|
| *Igf2*-P0 | F (Ex2) | GAGGAAGCTCTGCTGTTTGG | R (Ex6) | CAAAGAGATGAGAAGCACCAAC | 92 |
| *Igf2*-P1 | F (Ex3) | GACAAGGGTCTGACTTGGGA | R (Ex3) | CGTAGGAGAAGTGACGAGGC | 113 |
| *Igf2*-P2 | F (Ex4) | GTCGACCCTAACCGAGCTG | R (Ex4) | AAGCAGAGGAGAGGATGCAA | 103 |
| *Igf2*-P3 | F (Ex5) | TGGACATTAGCTTCTCCTGTGA | R (Ex5) | GCTGGAAGAGGATGAAGACAG | 61 |
| *Igf1* | F | GCTGGTGGATGCTCTTCAGTT | R | CTCATCCACAATGCCTGTCTG | 112 |
| *Ppia* | F | AAGGGTTCCTCCTTTCACAGAA | R | GATGCCAGGACCTGTATGCTT | 146 |
| *Pmm1* | F | ATCCGGGAGAAGTTTGTGGAA | R | GCTGTCTTCATCCAGGCTGTC | 144 |
| *Hprt* | F | CATTATGCCGAGGATTTGGAA | R | CCTTCATGACATCTCGAGCAA | 88 |
| *Tbp* | F | AACAACAGCCTTCCACCTTATG | R | TGTTCTGAATAGGCTGTGGAGT | 127 |
| *Gapdh* | F | ACAACTCACTCAAGATTGTCAGC A | R | ATGGCATGGACTGTGGTCAT | 121 |
| *Actb* | F | GATCAAGATCATTGCTCCTCCTG | R | AGGGTGTAAAACGCAGCTCA | 183 |
| *Acaca* | F | ACGTGCAATCCGATTTGTTGT | R | CCAGCCCACACTGCTTGTA | 180 |
| *Fasn* | F | TGCACCTCACAGGCATCAAT | R | GTCCCACTTGATGTGAGGGG | 104 |
| *Elovl6* | F | ACCCGAACTAGGTGACACGA | R | AGTCATGAACCAACCACCCC | 142 |
| *Scd1* | F | TGGTGAACAGTGCCGCGCAT | R | CGGCACCCAGGGAAACCAGG | 90 |
| *Acat2* | F | TACCTCAGTCGCAGACAGGA | R | TGAAGGAGCCTATAGCGGTG | 119 |
| *Hmgcs1* | F | CTGCTATTCTGTCTACCGCAAA | R | GGAACATCCGAGCTAGAGATTT C | 146 |
| *Hmgrc* | F | GGAATGCCTTGTGATTGGAGTT | R | CTCTAGGACCAGCGACACAC | 142 |
| *Abca1* | F | AGGGCATGTGGGAAGAACTC | R | TGTTCCCAAAACTGGTCATTGC | 115 |
| *Gpam* | F | TGCAACACTGAAATGGAAGGAG | R | ATAACATTCCGCAAACCCAGAG | 138 |
| *Agpat1* | F | AGCTCCAGTGCCAAGTATTTCT | R | GTATTTGACGTGGAGCAGCAG | 150 |
| *Lpin1* | F | GGAGACAACGGAGAAGCATTTT | R | GTTCCTCTTCAGCTGGCTTTC | 129 |
| *Lpin2* | F | CCTGAGGTCCAAGGAGAAAGT | R | GCTTCCCCATTATCACCCAATT | 87 |
| *Dgat2* | F | GCTGCAGGTCATCTCAGTACTA | R | TGCAGAAGGTGTACATGAGGAT | 89 |
| *Cd36* | F | TGCTGGAGCTGTTATTGGTG | R | GGTGCCTGTTTTAACCCAGTT | 148 |
| *Cav1* | F | ATACGTAGACTCCGAGGGACA | R | ACGTCGTCGTTGAGATGCTT | 174 |
| *Slc27a1* | F | TTCTCGTGGGCCAGATCAAC | R | AGCACGTCACCTGAGAGGTA | 136 |
| *Slc27a4* | F | CCAAAGCTGCCATTGTGGTG | R | ATCCCCACGATGTTTCCTGC | 136 |
| *Fabp4* | F | AGCTGGTGGTGGAATGTGTTAT | R | CCTCTTCCTTTGGCTCATGC | 76 |
| *Pnpla2* | F | GGAGGAATGGCCTACTGAACC | R | ATCCTCTTCCTGGGGGACAA | 71 |
| *Lipe* | F | CAAGCCCCAAAAGACCACATC | R | CTTCTTCAAGGTATCTGTGCCC | 127 |
| *Mgll* | F | AGACGGACAGTACCTCTTTTGT | R | ATGTCCAGCCCCTTCAACATAT | 135 |
| *Lipa* | F | AGATAATCATGCGCTGGGGATA | R | AAGATACACAACTGGTCTGGGA | 137 |
| *Ldah* | F | GTAGGCACCTATATGACCCTTC | R | CGATAGTTGGGAAGAGCAGAAA | 85 |
| *Lrp1* | F | GCCCACGTGCTACTGTAACA | R | ATGTGAAGGAGCCATCTGTGTT | 128 |

| | | | | | |
|---|---|---|---|---|---|
| *Pcsk9* | F | CTTGGTGAAGATGAGCAGTGAC | R | GGATAATTCGCTCCAGGTTCC | 122 |
| *Lpl* | F | TGTGAAATGCCATGACAAGTCT | R | CACTTTCAAACACCCAAACAAGG | 150 |
| *Srebf1* | F | ACTTTTCCTTAACGTGGGCCT | R | AGCTGGAGCATGTCTTCGAT | 153 |
| *Pparg* | F | TTTAAAAACAAGACTACCCTTTA CTGAAATT | R | AGAGGTCCACAGAGCTGATTCC | 95 |
| *Ttc36* | F | CCTTTGGAGATGTTGTTGGATT | R | CAACTGTGCTTGAGGGAAAACT | 80 |
| *Arhgdig* | F | AGATTGTCAGTGGCCTCAAATG | R | AATTCATACTCCTGGGCTCTGG | 112 |
| *Lep* | F | CCCAAAATGTGCTGCAGATAG | R | CCAGCAGATGGAGGAGGTC | 63 |

**Table S8. Primary and secondary antibodies used in Western blotting experiments**

| Antibody | Dilution | Supplier |
|---|---|---|
| Rabbit anti-phospho-AKT (Ser 473) | 1:1,000 | Cell Signaling Technology (#9271) |
| Polyclonal Goat anti-Mouse IGF-II | 1:2,000 | R&D Systems (#AF792) |
| Polyclonal Goat anti-Mouse IGF-I | 1:2,000 | R&D Systems (#AF791) |
| Rabbit polyclonal anti-SOD1 | 1:1,000 | Abcam (#ab183881) |
| Goat anti-Rabbit IgG (HRP) | 1:20,000 | Abcam (#ab6721) |
| Rabbit anti-Goat IgG (HRP) | 1:10,000 | ThermoFisher Scientific (#31433) |
